# A Systematic Review and Evidence Gap Map Evaluation of Rhythmic and/or Complex Movement Interventions and Child Cognitive Outcomes

**DOI:** 10.1007/s10567-025-00547-1

**Published:** 2025-10-11

**Authors:** Bronwyn M. Theroux, Elizabeth Eggins, Jessica Paynter, Sharon Dawe, Kate E. Williams

**Affiliations:** 1 School of Applied Psychology, Griffith University, Brisbane, QLD Australia; 2 Centre for Mental Health, Griffith University, Brisbane, QLD Australia; 3 Griffith Institute for Educational Research, Griffith University, Brisbane, QLD Australia; 4 School of Education and Tertiary Access, University of the Sunshine Coast, Sippy Downs, QLD Australia; 5 School of Education, Queensland University of Technology, Brisbane, QLD Australia

**Keywords:** Music, Rhythm, Movement, Children, Pre-adolescent, Intervention, Evidence and gap map, EGM

## Abstract

Optimal child cognitive developmental outcomes occur when the environment provides opportunities for challenge, development, and structure. For some children, additional support is required, with many interventions including a rhythmic element (e.g. instrumental music or group singing), or complex coordinated physical movement (e.g. structured sport or physical activity classroom breaks), to support cognitive development. While many studies examine the impact of these interventions, and some existing reviews examine effectiveness in discrete topic areas, the field requires a comprehensive overview and consolidation of the extant evaluation literature to guide future meta-analyses and primary studies (including replications). This systematic review and Evidence Gap Map identifies and synthesises studies that evaluate interventions where rhythmic and/or complex coordinated physical components are explicitly included. A systematic search of 17 databases yielded 402 studies that evaluate the impact of these interventions on cognition (including executive function and memory) using a randomised, quasi-experimental, or single group design in children aged birth to 12 years. Findings suggest that there is high saturation in the literature for interventions that include complex coordinated movement (62.70% of total sample) and moderate representation of rhythmic interventions (21.80% of total sample). Interventions that included both complex coordinated movement and rhythmic elements, such as martial arts, dance, or gymnastics, were sparse (15.50% of total sample). Children in the early primary school years (ages six to ten years) are the most common target population, and executive function is the most commonly measured outcome. Findings identified gaps in the literature for further research and evaluation to include interventions that incorporate both a complex coordinated movement element and a rhythmic element; incorporate the use of more rigorous randomised controlled study designs when evaluating these interventions; and use of a broader range of measures to capture different aspects of cognitive functioning (e.g. memory). These findings should inform the development and focus of future intervention studies, which in due course will lead to systematic reviews and network meta-analysis.

**PROSPERO Registration Number:** CRD42021248436.

**Supplementary Information:**

The online version contains supplementary material available at 10.1007/s10567-025-00547-1.

## Introduction

Cognitive development during childhood is influenced by a range of biological, social, familial, and psychological factors (Aranbarri et al., 2023). For some children, less than optimal development occurs in the context of social and economic disadvantage (Larson et al., 2015; Letourneau et al., 2013; Najman et al., 2004) and exposure to a range of childhood adversities including parental mental health and substance use problems, and child maltreatment (Fitzpatrick et al., 2015; McLaughlin et al., 2013). Early intervention is widely supported (Cattan et al., 2024; Diamond, 2016; Jeong et al., 2021) with extensive evidence underscoring the importance of delivery of programs within school settings (Barnett, 2011; Camilli et al., 2010; Diamond, 2010; Grande et al., 2022; Pickerell et al., 2023).

Numerous systematic reviews have synthesised the evidence of a range of interventions designed to improve children’s cognitive outcomes. For the purposes of the present review, ‘cognition’ is a broad conceptual term that includes a range of cognitive processes, including attention, memory, executive functioning, and problem-solving (Álvarez-Bueno, 2017; Diamond & Lee, 2011; Tomporowski & Pesce, 2019; van der Fels, 2015). Existing reviews typically focus on key components of the intervention such as mindfulness-based interventions (Mak et al., 2018; Maynard et al., 2017; Zenner et al., 2014), nutrition and diet (Roberts et al., 2022), sport (Contreras-Osorio et al., 2021), exercise (Xue et al., 2019), physical activity (Alvarez-Bueno et al., 2017), music training (Román-Caballero et al., 2022), exergaming/virtual reality/immersive gaming (Costa et al., 2019), and social or peer competence (Sancassiani et al., 2015). Overall, past reviews indicate a degree of promise in supporting the improvement of cognitive outcomes.

Notably, one investigated approach includes interventions with a rhythmic element (i.e. movement to beat or copying rhythmic patterns) or that had as a core feature, complex coordinated movement that incorporated coordinated body movement and a cognitively complex component (i.e. rule memorisation or increased cognitive load) (Thaut, 2013; Thaut et al., 2014). Those in which rhythm is a core element involve a clear, regular, repeated pattern of sound, with beat synchronisation requiring the coordination of an individual’s purposeful, rhythmic movement to an externally provided beat, such as music, clapping, or tapping (Frischen et al., 2019; Williams & Berthelsen, 2019; Williams et al., 2023). Interventions that focus on complex coordinated movement that include active cognitive engagement—compared to incorporating simply aerobic exercise—appear to confer greater cognitive benefit (Diamond, 2015; Diamond & Lee, 2011). There are also investigations of programs or interventions that combine both rhythmic elements and complex coordinated movement. For example, a program that requires children to move in time with music, requiring processing of the beat, memorisation of an action sequence, and synchronisation of movement to rhythm, aimed to target self-regulation in pre-primary aged children (Williams & Berthelsen, 2019). A further example is martial arts programs, where the performance of memorised complex movement sequences requires cognitive processing of sounds that provide a cue and inhibition of movement when similar but non-cueing sounds are heard (Lakes et al., 2013). The use of music or rhythm as an ‘action cuing’ mechanism combines complex movement with attentive music processing and synchronisation and is supported in research evaluating dance (Lobo & Winsler, 2006; Shen et al., 2020; Sousa Junior et al., 2023), drumming (Willemin et al., 2018; Yoo & Kim, 2018), and martial arts interventions (Greco & De Ronzi, 2020; Lakes, 2013; Lakes & Hoyt, 2004; Lakes et al., 2013).

### The Evidence for Music-Based Interventions

We identified 12 reviews that examined the impact of music-based interventions for improving cognitive outcomes for children and adolescents. Early reviews investigated music interventions in paediatric healthcare settings on health and clinical outcomes in children aged 18 or less, finding significant improvements in health outcomes for children with developmental or learning disorders, and overall improvement with cognitive functioning and recall (Treurnicht Naylor et al., 2011). Outside of health settings, other reviews have examined music education interventions for children aged 4–13 years across outcomes of intelligence, reading, spatial reasoning, writing, and mathematics (Jaschke et al., 2013). Findings indicate that poor study quality and methodology produced a lack of overall significant effects (Jaschke et al., 2013), a conclusion echoed in a narrative review of the music training literature (Miendlarzewska & Trost, 2013). An early meta-analysis of music training interventions in relation to academic and cognitive outcomes for children aged 3–16 years found a small overall effect, with the strongest effects for intelligence and memory outcomes (Sala & Gobet, 2017). Music training has had a great interest in reviews more recently in this age range, with small-to-moderate effects in overall cognitive outcomes (Cooper, 2019) and small significant effects in music training averaging 17 months duration for academic and cognitive outcomes (Román-Caballero et al., 2022). When focused on executive functioning outcomes in early and middle childhood (0–10 years), interventions appear to have the greatest effect on inhibitory control, along with being the most common executive function measured (Degé & Frischen, 2022), with moderate-to-large effects on inhibitory control in early to middle childhood (Jamey et al., 2023).

Some interest has been directed towards specific clinical groups regarding the impact of music interventions. Reviews have found positive effects of educational music therapy with Autistic children, particularly with regard to speech production, and a positive effect on social functioning across neurodevelopmental disorders (Mayer-Benarous et al., 2021). Further scoping reviews focused specifically on therapeutic drumming with Autistic individuals for mental health and wellbeing outcomes, finding consistency in intervention implementation but inconsistency in outcome measures, thereby limiting efficacy analyses (Friedman et al., 2023). Active music making, colloquially termed ‘musicking’, with adolescents has been utilised to promote mental health and has demonstrated improvements in social–emotional wellbeing outcomes and a reduction of internalising symptoms for adolescents (Rodwin et al., 2022), with moderate-sized effects found across the child and adult lifespan on emotion regulation (Peters et al., 2024).

### The Evidence for Movement and Physical Activity Interventions

We identified 23 reviews of studies with children and adolescents relating to movement or physical activity interventions. One umbrella review from Biddle and colleagues (2019) focused on measures of physical activity and mental health outcomes and investigated the correlative associations between these outcomes in school-aged children, rather than focusing on specific interventions. Findings supported a strong association between physical activity and cognitive functioning, with partial support for associations with depression outcomes, and no support for associations with self-esteem outcomes (Biddle et al., 2019). In their own literature review, Vazou and colleagues (2019) conducted an important search within previous reviews relating to physical activity and movement published from 2003 onwards (i.e. (Barenberg et al., 2011; Best, 2010; Diamond & Lee, 2011; Diamond & Ling, 2016; dos Santos et al., 2013; Fedewa & Ahn, 2011; Keeley & Fox, 2009; Pesce et al., 2009; Pesce et al., 2013; Sibley & Etnier, 2003; Tomporowski et al., 2015; van der Fels et al., 2015). The review concluded that physical activity interventions, including complex physical activity with a cognitive component, had overall significant small-to-moderate effects on cognition in children aged 0–18 years (Vazou et al., 2019).

Meta-analyses have primarily focused on school-aged children and physical activity, finding medium effects for nonexecutive functions, executive functions, and meta cognitive functions (Alvarez-Bueno et al., 2016), overall improvement in executive function across longer term exercise interventions (Xue et al., 2019), improvement in self-regulation (Pandey et al., 2018), and improved classroom behaviour (Masini et al., 2020) and academic and cognitive outcomes (Laurent et al., 2021) in school-based interventions. A systematic meta-review of long-term physical activity interventions for cognition across the lifespan found that motor enrichment is a specific key condition that relates to mechanisms of change, and in turn results in greater physical activity effects on cognition (Pesce et al., 2023). Furthermore, Pesce et al. (2023) concluded that studies with ‘mindful movement’, or a spatial engagement mechanism underlying executive function improvement, were the only interventions that provided consistent evidence of entirely (100%) positive outcomes. More specific reviews regarding intervention genres include sport interventions, which tend to find large effects for executive functions (working memory, inhibitory control, cognitive flexibility) for children aged 6–17 years (Contreras-Osorio et al., 2021). Further, exergaming interventions tend to have high impact for cognitive and physical rehabilitation in children and young adults (Costa et al., 2019).

School-based interventions that include classroom-based physical activity interventions were reviewed in relation to cognitive outcomes and found improved attention and on-task behaviour in school-aged children aged 4–18 years, noting overall benefit, but with high variability in intervention methodology and implementation (Ruhland, 2021). Furthermore, physically active school academic lessons improve academic performance in children aged 3–11 years, as well as motor skill and physical activity (Petrigna et al., 2022). These findings were also consistent with a movement intervention review by Mavilidi and colleagues, with a meta-analysis finding children aged 2–18 years showed improved academic, memory, and behavioural outcomes. Yet, they did not show consistent improvements in cognitive processing. Specifically, long-term academically integrated interventions have large effects on memory and short-term non-academic interventions have large effects on behaviour control (Mavilidi et al., 2022). Finally, a focused review and meta-analysis examining the impact of cognitively engaging physical activity intervention on executive functions in children aged 4–12 years found few studies (11 articles total), and findings revealed positive effects on executive functions, such as small-to-moderate effects sizes for updating and shifting (Song et al., 2022).

### Evidence and Gap Map Rationale

Although a number of reviews have been conducted on the impact of rhythmic, musical, and movement interventions for child cognitive outcomes, the primary focus has been on evaluating discrete therapies or supports and utilising a range of different outcomes. Further, these reviews have not consistently used rigorous review methodology (Page et al., 2021), encapsulate narrow search dates, or have used a narrow focus to the extent that few studies are included. Therefore, there is a need for a comprehensive and methodologically rigorous systematic review to synthesise the diverse evaluation literature across music and movement intervention modalities and identify the gaps and areas for future research interests. Further, while a suite of existing reviews has focused specifically on rhythmic *or* movement interventions, an emerging area of research indicates that a focus on the integration of *both* modalities within interventions may hold promise. The shared neural networks for rhythm processing, movement, and cognitive development suggest that when targeted together in a single intervention, superior benefits may occur (Diamond, 2012; Diamond & Lee, 2011; Diamond & Ling, 2016). It is important to consider if these intervention modalities, that hold great promise, have specific intervention methodology, such as implementer and setting or session number, frequency, and duration, that should be considered and may inform future research.

Only one theoretical umbrella review has taken a common intervention element approach, reviewing across the literature of exercise, sport, and performance arts focusing on the causal relationship of physical movement and cognition (Tomporowski & Pesce, 2019). The umbrella review concluded that essential commonalities such as the process of skill acquisition and engaging in complex cognitive engagement simultaneously with physical movement are underlying mechanisms that impact cognitive benefits (Tomporowski & Pesce, 2019).

In this review, we conduct the first evidence and gap map (EGM) in the area of rhythmic and/or complex coordinated movement interventions for cognitive outcomes in children. EGMs are one form of synthesis that can be used within an overarching systematic review methodology. They differ from systematic reviews of intervention effectiveness in that they do not aim to synthesise intervention effectiveness via meta-analyses. Rather, EGMs use systematic review methods to rigorously identify evaluation studies in a broad area of research and then summarise the *nature* of the research in visual format (White et al., 2020). This enables the investigation of gaps in research, with EGMs providing a unique and efficient tool to represent a large body of research evidence, including the breadth of research methodology and inclusion of different populations and outcomes (Snilstveit et al., 2016). The EGM approach is also often used when there is emerging evidence in a research field, and maps areas of disparity or ‘gaps’ in research (Snilstveit et al., 2016). There is also increasing use and funding of the EGM approach to consolidate large bodies of evidence to inform future meta-analyses or primary intervention research, which are then used to inform policy, practice, and investment in future research (e.g. Eggins et al., 2021; Finch et al., 2021; Pundir et al., 2020; Sydes et al., 2023; White et al., 2021). An EGM offers an optimal interactive experience for readers to explore study features, as well as use filters to descriptively understand a topic based on their specific needs or interests (Snilstveit et al., 2016). We argue that the extant evidence relating to rhythmic and/or complex coordinated movement interventions for cognitive outcomes in children requires the integration and consolidation that an EGM can provide.

Thus, the aim of our systematic review and EGM is to (1) provide the first synthesis of two key areas of intervention literature, complex coordinated movement and rhythm; (2) map the intersection of these two areas of literature with interventions that feature both elements; and (3) synthesise the breadth of the literature to date by visualising areas of saturation and disparity regarding study design, participant characteristics, intervention methodology, and the broad general outcomes utilised.

### Objectives of the Review and EGM

We aimed to systematically gather and map the extent of randomised control trial (RCT), quasi-experimental and single group pre–post-design evaluation studies of rhythmic and/or complex coordinated movement interventions in children aged birth to 12 years for cognitive outcomes (cognition, executive function, memory). We focus on this age range to capture early developmental stages when children undergo rapid formative changes across cognitive, emotional, social, and behavioural domains at a stage that may be most responsive to intervention. Limiting to this age range avoids potentially confounding impacts of adolescence and high school where marked changes in educational settings and the influence of peer and social relationships are separate, albeit important, considerations that need to be considered when developing intervention for this older age group. By presenting an EGM of the current literature, we synthesise its breadth, mapping the nature of interventions across participant age and characteristics (age, gender, clinical groups), nature of the intervention (including implementers, setting, modality, dosage, frequency, duration) and outcomes measured. This is vital to advance the field by summarising the scope, quantity, and characteristics of the diverse body of literature. This analysis will identify under-researched areas of populations, highlight imbalances in study designs, inform future research priorities (by clarifying where there is a high amount of research and where research is needed), and support the avoidance of duplication in future to advise more strategic research in areas of gaps.

## Methods

This systematic review and EGM adheres to the Preferred Reporting Items for Systematic Review and Meta-Analyses (PRISMA) (Page et al., 2021) and the Campbell Collaboration Evidence and Gap Map Conduct and Reporting Checklists (White et al., 2020). The protocol for this review was registered in April 2021 with the Prospective Register of Systematic Reviews (PROSPERO Number: CRD42021248436). There was one deviation from protocol, with the inclusion of single group quasi-experimental studies.

### Search Strategy

A systematic search for studies was conducted in September 2021 and then updated in April 2025 (see Supplementary Material for the full search record). The search encompassed 17 electronic databases of published peer-reviewed and unpublished literature: *Campbell Systematic Reviews*, Cochrane Collaboration (Cochrane Database of Systematic Reviews; Cochrane Central Register of Controlled Trials (CENTRAL); Database of Abstracts of Reviews of Effectiveness), EBSCO (CINAHL, Education Source, ERIC, SPORT Discussion), Elsevier (Embase), OVID (MEDLINE, PsycARTICLES, PsycEXTRA, PsycINFO), ProQuest (Dissertation and Theses Global, International Bibliography of the Social Sciences, Psychology Journals, Research Library, Social Science Database, Music Periodicals Database), and Web of Science (Web of Science Core Collection, Arts & Humanities Citation Index, Social Science Citation Index, Conference Proceedings Citation Index). Groups of search terms were developed and tested across four concepts aligned with the review inclusion criteria: population (children), outcomes (cognition, executive function, and/or memory), intervention (rhythmic and/or movement), and study design. Search terms within each concept were combined with Boolean OR and proximity terms, and the search concepts were then combined with Boolean AND. Wherever possible, searches were run across the title, abstract, author supplied keywords, and subject index fields.

### Inclusion Criteria

Eligibility and study selection followed a PICOS (participant, intervention, comparison, outcome, study design) framework (Higgins et al., 2019). Studies were included if authors (1) utilised a population of participants (children or children-carer dyads) aged 0 to 12 years (or mean age of < 12.5 years) to (2) evaluate an intervention that included elements defined as rhythmic and/or complex coordinated movement that lasted a minimum of two sessions, using (3) a standardised direct and/or indirect measure of cognition, memory, and/or executive function. Eligible study designs included randomised and quasi-experimental designs of any kind, and eligible comparators were waitlist control, treatment-as-usual, active control, or baseline pre-treatment measures (i.e. single group pre–post-studies).

For the purposes of this review, we considered a range of outcomes captured under the overarching cognition umbrella, including memory, and executive function. To determine which category a measure belonged to, we either used study authors’ definition or consulted with neuropsychological texts and previous reviews to confirm the outcome classification (e.g. Betts et al., 2022). The broad use of the term ‘cognition’ has been previously used in reviews to capture a wide range of valid outcome assessments for broad-scale reviews (i.e. Álvarez-Bueno et al., 2017; Tomporowski & Pesce, 2019; van der Fels, 2015). In order to be eligible, outcomes needed to be measured using a standardised, valid assessment by either direct assessment of the child or by indirect assessment via reports from others (e.g. parents/caregivers, educators). Examples of outcomes that were coded for executive functioning include, but are not limited to working memory, inhibition, cognitive flexibility, and attentional control. Examples of acceptable valid executive function assessments include the following: NIH Toolbox, Early Years Toolbox, Stroop Task, and Go/No-Go Task. Examples of outcomes that were coded for memory include, but are not limited to verbal memory tasks, non-verbal memory tasks, spatial memory tasks, auditory memory, delayed-recall, and cued recall. Examples of acceptable valid memory assessments include the following: Rey’s Auditory Verbal Learning Test, McCarthy Scales of Children’s Abilities: Pictorial Memory. Examples of outcomes that were coded for general cognition include, but are not limited to verbal and non-verbal reasoning, information processing, spatial processing, comprehension, and problem-solving. Examples of acceptable valid cognitive assessments include the following: WISC-IV (i.e. FSIQ, Matrix Reasoning, Coding, Comprehension), Stanford-Binet Fifth Edition (i.e. Nonverbal Visual-Spatial Processing, Verbal Visual-Spatial Processing), and the Kaufman Brief Intelligence Test (i.e. Matrices).

Complex coordinated movement interventions were broadly defined as those that included movement components that involved a cognitive challenge and extended beyond fundamental movement skills (Diamond, 2015; Diamond & Lee, 2011). The term ‘complex coordinated movement’ was defined using inclusion criteria from multiple sources in the literature where various complex movement criterion were outlined (Tomporowski & Pesce, 2019). To be included under this category, the intervention needed to include at least one of the following components while the participant engaged in complex movement: bilateral coordination; multi-system learning (simultaneously engaging physical and mental systems); proprioception (awareness of the body’s positioning/movements in space, mirroring of two or three dimensional instructive demonstration); synchronising movement with others (moving with a partner, coordinating actions with partner sequentially, movement with an instructor); object control (sport-related ball/equipment use, non-sport-related coordinated object use); cognitively engaging activity (requiring two or more cognitive demands or two or more memorisation components), or memorised action sequences (practice and repetition of memorised action with or without added components of difficulty). At least one of these elements needed to be present for the majority of the intervention, defined as 75% or greater of the overall intervention dosage captured through total intervention session time. Excluded interventions under this category included fundamental movement skills with repetitive action without change (e.g. cycling-only, running-only, walking-only) or interventions with expert-level populations (e.g. child sport prodigies).

The term ‘rhythmic’ activity was defined using inclusion criteria or intervention descriptions from multiple sources in the literature where various rhythmic intervention criterion were outlined (Román-Caballero et al., 2022). Rhythmic interventions needed to include at least one of the following elements as part of the intervention: rhythmic auditory cuing (call and response); rhythmic inhibition (inhibiting impulsive sounds/music/actions, swapping roles or musical cues); rhythmic synchrony (syncing the production of sound or movement to a rhythmic stimulus); music learning; music reading or other forms of repetitive practice (site reading, pre-composed musical pieces, orchestral music composition); and/or beat synchronisation (defined as either musical instrument engagement (i.e. drumming), or basic movement engagement (i.e. clapping)). Musical instrumental instruction interventions were eligible, provided the participant was actively engaging with the instrument. At least one of these elements is needed to be present for the majority of the intervention, defined as 75% or greater of the overall intervention dosage captured through total intervention session time. Exclusion criteria were passive music engagement studies (e.g. music listening, musical instrument observation), unstructured exploratory music interventions (play-based or exploratory-based music engagement without instruction, structure, or standardised program implementation), and therapeutic interventions where music was used as a means of expression only (e.g. selecting a song to listen to, based on an expressed emotion without further engagement with the stimulus and no musical production).

Interventions that were focused on mindfulness, such as yoga, were excluded from the review due to the wide and unique evidence base that exists for these interventions (i.e. Birdee et al., 2009; Ferreira-Vorkapic et al., 2015; Filipe et al., 2021; Khalsa & Butzer, 2016; Maynard et al., 2017; Weaver & Darragh, 2015; Zenner et al., 2014), and the proposed mechanism for change for mindfulness which may be theoretically unique to these interventions (Schuman-Olivier et al., 2020; Tudor et al., 2022). For the same rationale, animal therapies, including hippotherapy, were excluded from the intervention eligibility due to the unique and recent evidence base (Maresca et al., 2022; Xiao et al., 2023).

No restrictions were placed on population parameters (e.g. clinical or neurodivergent versus neurotypical, typically developing, or non-clinical groups), intervention setting, or publication date. Studies needed to be published in English to be included, though any non-English studies were screened on their abstract for preliminary eligibility and a full list of potentially eligible non-English studies can be provided on request.

### Study Selection and Data Extraction

The systematic search was de-duplicated using EndNote X9 citation manager software and imported into *DistillerSR* review software (*DistillerSR*, Evidence Partners, Ottawa CA) for study section and data extraction. Initially, identified studies were screened for eligibility based on their title and abstract. Studies then progressed to full-text screening if they were unique (i.e. not a duplicate), an eligible document (i.e. not a book review, news item, etc.), and reported on what appeared to be an eligible intervention conducted with children aged < 12.5 years. To reduce workload at title/abstract stage (search yield: 46,120), *DistillerSR*’s continuous reprioritisation feature was utilised, where artificial intelligence is employed to reorder unscreened title/abstract studies based on their predicted relevance, and paired with the software’s auditing and simulation tools helped to identify potentially ineligible articles based on the reviewers pattern of decision making (Hamel et al., 2020). When *DistillerSR* identified that 95% of potentially eligible documents had been identified, screening was ceased if a consecutive set of 50 documents did not meet eligibility criteria (see Eggins et al., 2024; Sarma et al., 2022; Sydes et al., 2023).

The full texts for potentially eligible studies from the title/abstract screening stage were then appraised for final eligibility by two authors (BT, EE). Full-text articles were screened for final eligibility according to the inclusion criteria outlined above. Once deemed eligible for inclusion, studies were categorised by research design, intervention category (complex coordinated movement, rhythmic, both), and outcome category (cognition, executive function, memory). Two authors (BT, EE) screened documents at both the title/abstract and full-text screening stages, with a third author used to resolve any discrepancies or ambiguities regarding inclusion threshold ‘edge’ cases (SD). Trial registries or protocols were included in a list of ongoing studies (if not linked to a completed included study). Where authors were unable to access full texts of the documents screened eligible at title-abstract Level 2 screening, or unable to screen beyond the abstract due to the body of the full text being available in a language other than English, a range of actions were taken to obtain an eligible full-text copy of the document. First, library orders were made via institutional libraries to obtain copies of the full-text document. Second, document authors were contacted via email or other platforms (e.g. ResearchGate) to gain access to the document. If these actions failed to retrieve the full-text document, the study was categorised as ‘awaiting classification’, with the list of these references provided in supplementary materials.

Data were then extracted by two authors (BT and EE) for all eligible studies using standardised coding forms (see Supplementary Material) and included setting details (country, publication type), population details (recruitment, attrition, age, sex, ethnicity, and clinical diagnosis), intervention (program name, intervention category, delivery modality, implementor, duration, dosage, and frequency), outcome (measure construct, method, and measure name), and research design characteristics (study design and comparator details). Secondary reports of the same included study were grouped under a ‘parent’ study so that each study represented one data-point in the coding framework and map.

### Data Synthesis and Mapping

Each study was categorised into an overall framework to enable visualisation in an Evidence and Gap Map (EGM). The term ‘Evidence and Gap Map’ is now a standard term used in the evidence synthesis community (e.g. Snilstveit et al., 2016; White et al., 2020) and reflects that the synthesis approach (i.e. mapping) visualises the nature of evaluation evidence (i.e. studies). Use of the word ‘evidence’ in this review refers to the evidence of evaluation studies, not evidence in the context of effectiveness. Different map dimensions and filters were used to visualise the study corpus in different ways, explained in further detail below, using EPPI-Reviewer (Thomas et al., 2023). Four broad categories were produced in EPPI reviewer: participant, intervention, outcome, and research study. These broad categories included multiple discrete categories (between three and five) that were coded and collapsed for different visualisations.

## Results

### Systematic Search

**Fig. 1:**
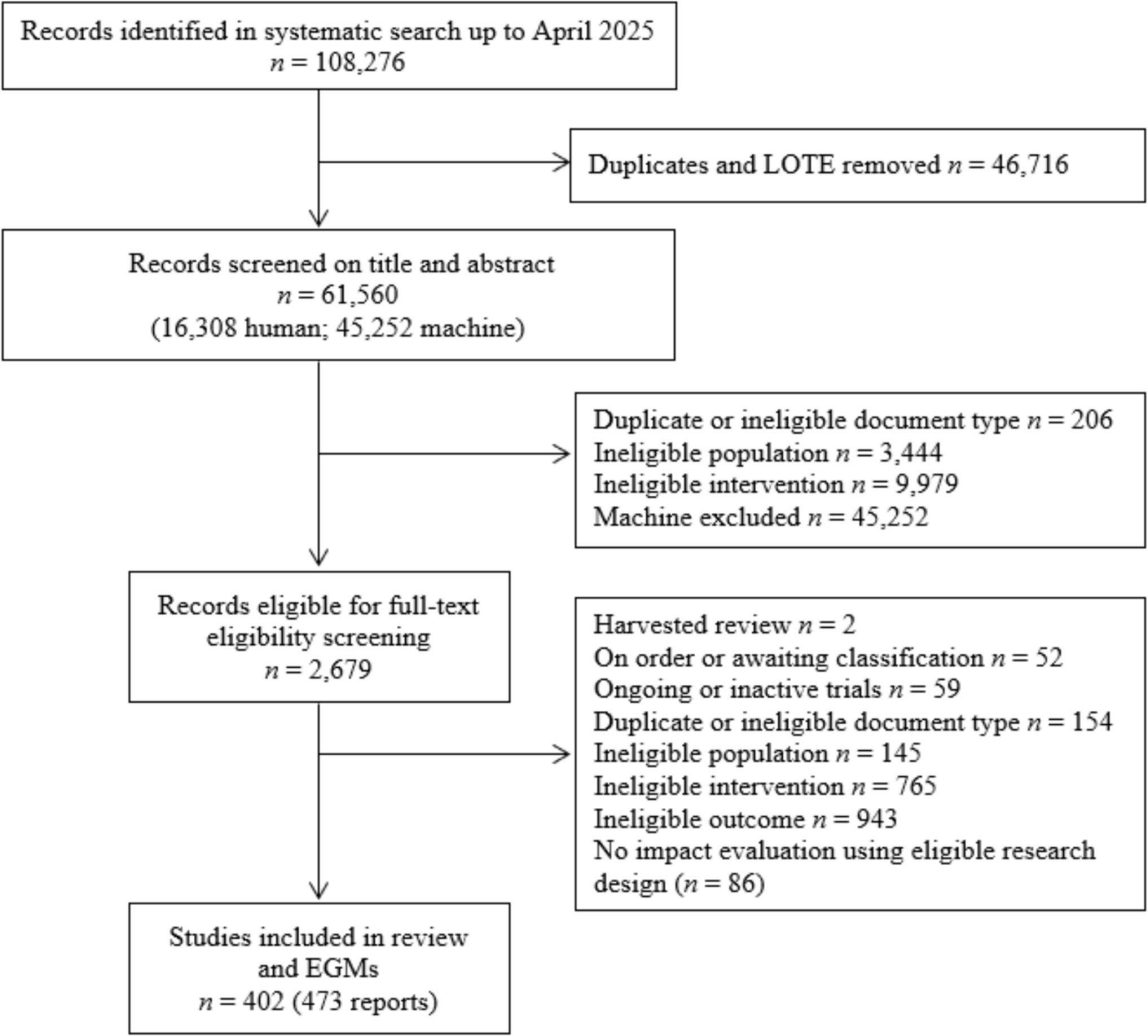
PRISMA flow diagram

The systematic search identified 61,560 unique records with 58,881 excluded at the title/abstract screening stage. Of the 2679 records proceeding to full-text eligibility screening, 52 could not be located with existing resources or interlibrary loans. The remaining 2627 documents were screened, with 2154 excluded for the following reasons: (1) previous unidentified review for harvesting (*n* = 2); (2) duplicate or ineligible document type (*n* = 154); (3) ongoing or inactive trials, either protocols or trial registries (*n* = 59); (4) ineligible population (mean > 12.5 years) (*n* = 145); (4) ineligible intervention (*n* = 765); (5) ineligible outcome measure (*n* = 943); or (6) no quantitative impact evaluation using eligible participants, intervention, or outcome (*n* = 86). A total of 402 studies, reported in 473 documents were eligible for inclusion in the review. See Fig. 1, PRISMA Flowchart.

### Characteristics of Included Studies

**Fig. 2:**
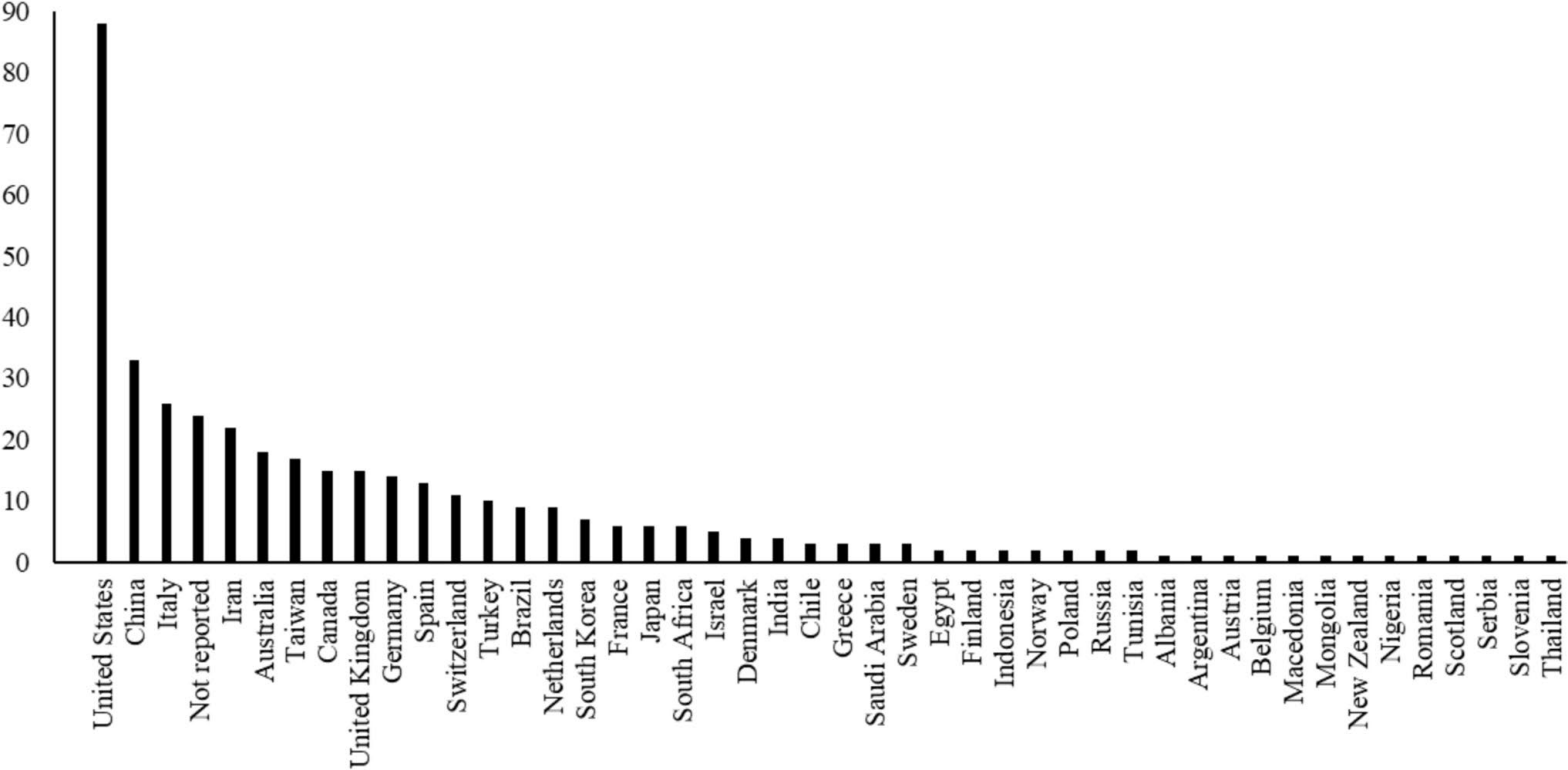
Study country frequencies

The 473 included documents (excluding ongoing studies) were published between 1966 and 2025 and were reported in a range of document types, including peer-reviewed journal articles (*n* = 372), dissertations/thesis (*n* = 55), conference presentations or posters (*n* = 16), technical reports (*n* = 1), protocol or trial register (*n* = 25), and book chapters (*n* = 2). Within the 473 included documents, 486 unique comparisons were reported (i.e. between treatment and control group or between two eligible treatments), across 402 studies. Many studies had more than one document or report associated with the evaluation (i.e. clinical trial register, protocol, and peer-reviewed journal article) and many studies had more than one eligible comparison between groups. Each unique comparison from a study was counted as a row in the EGMs, which meant that each study could represent more than one row in the maps. For example, a study may have had two treatment arms and one control group, meaning that each possible comparison was used as a separate row in the map (e.g. treatment 1 versus control, treatment 2 versions control, treatment 1 versus treatment 2). Most studies were conducted in the United States (*n* = 88), followed by China (*n* = 33), and Italy (*n* = 26) with a small group of studies not reporting the location of the study (*n* = 24), see Fig. 2 for all study countries.

### Sample Size and Sociodemographics

The included studies captured approximately 17,506 participants in total (reporting quality variable across studies) and the sample size for individual studies ranged from 5 to 1608. The mean age range for the total studies was from 7.40 months to 12.50 years. Almost a third of all included studies (*n* = 132; 33.08%) utilised samples where the target population for participants fell into a clinical category, for example, ADHD (*n* = 50; 12.44%) and Autism (*n* = 26; 6.47%). The majority of included studies (*n* = 393; 97.76%) were conducted with children only, with only 9 (2.23%) studies conducted with children and caregivers.

### Interventions

Of the 402 studies included, there were 445 interventions eligible for the three global categories of coding, where some studies had multiple eligible studies or treatment-comparison arms. Of the interventions, 62.70% (*n* = 279) were complex coordinated movement interventions, 21.80% (*n* = 97) were rhythmic or music-based interventions, and 15.50% (*n* = 69) included both complex coordinated movement and rhythmic interventions.

#### Setting and Implementation Intervention

Of the 445 eligible interventions, 319 (71.69%) were held at an educational facility (preschool, kindergarten, childcare or daycare, elementary school, primary school, or outside-of-school-hours care), 50 (11.24%) were at sport or music specialised facilities, 29 (6.52%) were held at health clinics or hospitals, 11 (2.47%) were at home, and 36 (8.09%) did not report the setting location. Implementors of the interventions were predominantly (*n* = 159, 35.73%) professionals qualified in the specific areas of music or movement (qualified coaches, music/instrumental instructors, dance instructors, or specially trained instructor) or teachers (*n* = 147; 33.03%), followed by health professionals (*n* = 52; 11.69%) and parent/caregivers (*n* = 8; 1.80%). A large number of studies (*n* = 79; 17.75%) did not specify who implemented the intervention.

#### Intervention Methodology

Most interventions were conducted in a group format (*n* = 393; 88.32%), followed by an individual format (*n* = 42; 9.44%). A small number of studies did not report the delivery format (*n* = 10; 2.25%). The modality of the intervention varied, with the majority of interventions provided face-to-face (*n* = 412; 92.58%), with a smaller number delivered audio visually (*n* = 23; 5.17%), or unspecified (*n* = 10; 2.25%). Studies reported the number of sessions for the intervention, with the average number of sessions of 36.40 (SD = 43.86), with a range of 3 to 360 sessions. A portion of studies did not specifically report the number of intervention sessions (*n* = 56; 12.58%).

The frequency of intervention sessions was most often once or more weekly (*n* = 420; 94.38%), followed by daily (*n* = 4; 0.90%), and fortnightly (*n* = 1; 0.23%). Again, a portion of studies did not report session frequency (*n* = 20; 4.49%). Studies that had their session frequency weekly were held on average 3.16 times per week (SD = 2.83). Studies that had their session frequency daily were held on average 1.75 times per day (SD = 0.96). The length of sessions varied across the studies: 7 interventions (1.57%) implemented sessions of less than 5 min, 26 interventions (5.84%) with sessions between 5 and 14 min, 36 interventions (8.09%) between 15 and 29 min, 179 interventions (40.23%) between 30 and 59 min, 95 (21.35%) of 60 min, 45 interventions (10.11%) were 61 and 90 min, and 16 (3.60%) were greater than 90 min. Some interventions (*n* = 6; 1.35%) had varying session lengths, and 35 studies (7.87%) did not report the intervention session duration.

### Outcomes

Of the 402 included studies, 82.34% (*n* = 331) utilised direct outcome measures, 6.47% (*n* = 26) used indirect measures (adult report), and 11.19% (*n* = 45) implemented both direct and indirect outcome measures. Most studies utilised multiple validated measures, with a total of 1046 direct measures reported, and 87 indirect measures reported. Across the broad outcome categories, 320 studies (79.60%) measured executive function directly and 68 (16.92%) measured executive function indirectly. Ninety-five studies (23.63%) measured general cognitive abilities directly and 3 (0.75%) measured these indirectly. Memory outcomes were the least common with 29 studies (7.21%) measuring memory directly, and zero measuring memory indirectly.

### Research Design and Comparators

Half of the included studies (*n* = 193; 48.01%) were randomised control trials (either cohort, group, or individual randomisation) and 209 (51.99%) were quasi-experimental research designs. Of the 209 quasi-experimental designs, 101 (48.10%) used unmatched comparison groups, 51 (24.29%) used a matched comparison groups, 40 (19.05%) were single group pre–post, 10 (4.76%) used a crossover design, and 7 (3.33%) used a quasi-randomised design. Comparison groups were labelled by authors as mostly standard no intervention control groups (*n* = 193; *n* = 48.01%), followed by alternative treatments (*n* = 58; 14.43%;), waitlist controls (*n* = 38; 9.45%;), treatment-as-usual (*n* = 37; 9.20%), and active controls (*n* = 36; 8.96%;). Forty studies (9.95%) used a baseline measure of the outcome as the comparator with a single group design. Forty-three studies (10.70%) included multiple comparisons in the data set. Of the studies with multiple comparisons, two (*n* = 39; 90.70%) or three (*n* = 4; 9.30%) treatments were eligible intervention arms, in addition to the comparison group.

#### Evidence and Gap Map

**Fig. 3:**
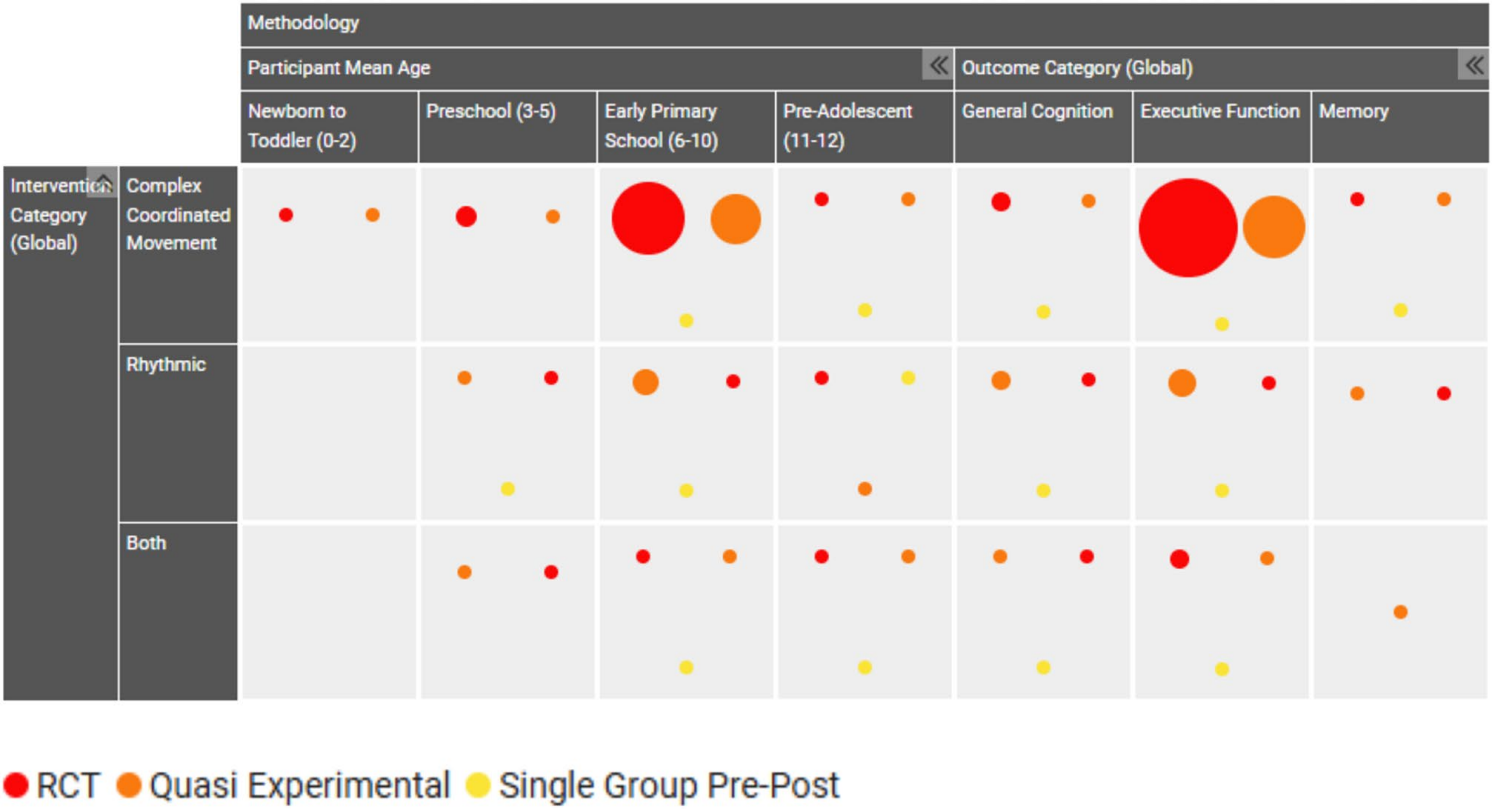
Evidence and gap map 1. Intervention categories, participant age, and outcomes filtered by study design. Interactive EGM 1 Hyperlink: https://figshare.com/ndownloader/files/56231684

**Fig. 4:**
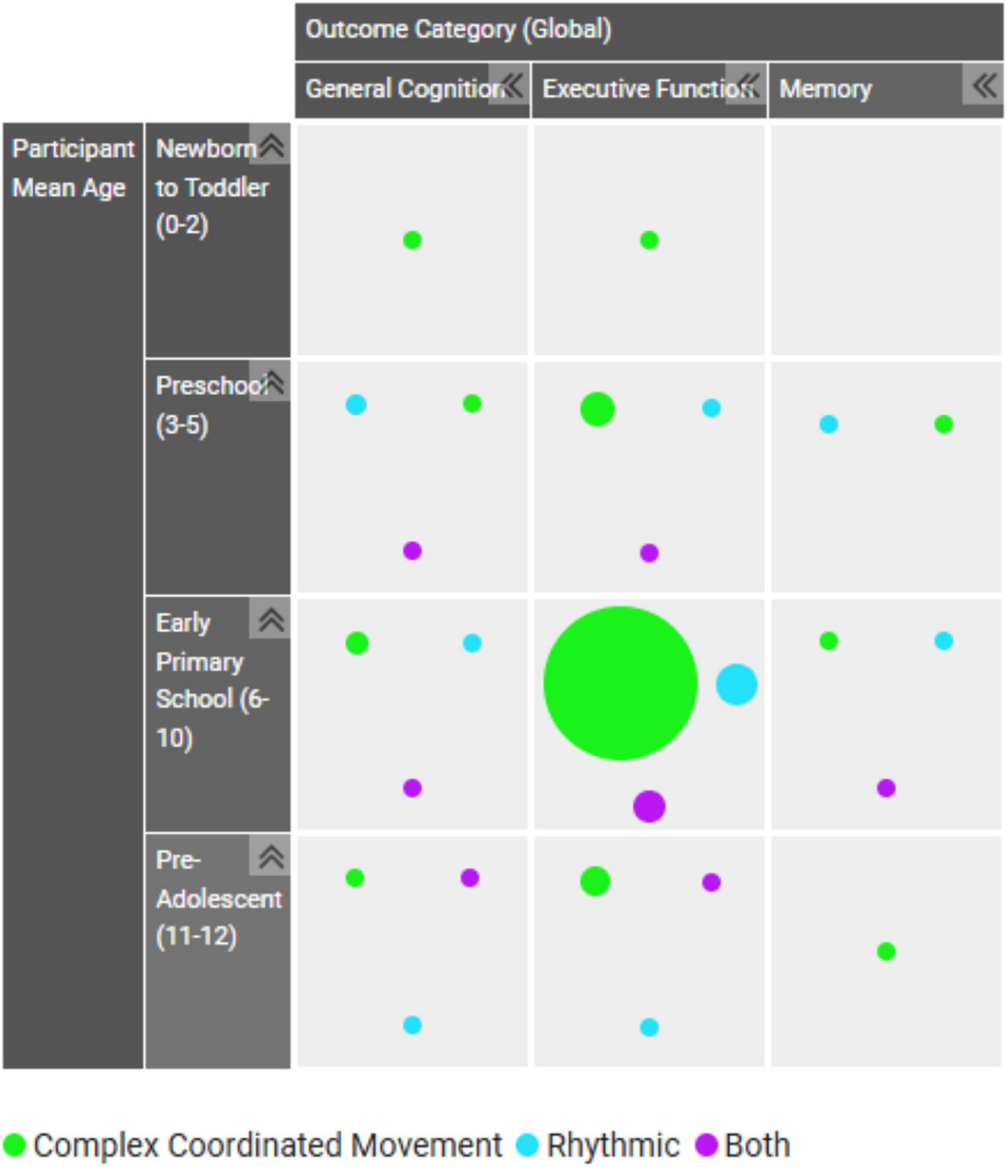
Evidence and gap map 2. Participant age and outcomes filtered by intervention categories. Interactive EGM 2 Hyperlink: https://figshare.com/ndownloader/files/56231720

An EGM was developed to capture the breadth and depth of evidence and study characteristics included in the review. Available data for 402 studies (reported across 473 reports/documents) were synthesised and presented across the five key coding domains: study summary, study design, participant characteristics, outcomes, and intervention methodology. Each coding domain contained global and specific study and methodology information, as depicted in Fig. 3 (EGM 1) and Fig. 4 (EGM 2) in static maps. For the intervention map including fully detailed coding see: https://figshare.com/ndownloader/files/56231741

Links are embedded in text to allow for full interactive features, including navigation, filtering, and reference lists on an external browser.

Circles on the EGM can be located on the legend to find corresponding colour representation, with dots intersecting across two axes (horizontal and vertical) representing a study that included both axis descriptors. Circle size represents the number of studies for that particular intersection of axis. Areas with an absence of circles represent an absence of study representation, thus highlighting areas of gap for further research.

As can be seen, the representation of the three global categories follows the results presented, where there is a high representation of studies evaluating complex coordinated movement interventions, followed by rhythmic interventions, and across methodological categories an under representation of interventions that include both complex coordinated movement and rhythmic elements.

## Discussion

This systematic review and Evidence and Gap Maps provide a synthesis of studies that evaluated the impact of rhythm, complex coordinated movement, or interventions with both elements on cognitive outcomes in aged 0–12 years. A comprehensive search and review were conducted to identify studies across research domains, to synthesise the literature of complex movement and/or rhythmic interventions for children evaluated by any valid cognitive outcome measure. The search yielded 402 studies across 473 documents published between 1966 and 2025. The EGMs presented highlighted key areas of high representation of study components, and key areas of gaps of the evidence where future research may further investigate.

### Summary of Findings

The findings demonstrate a clear gap for interventions that include both a complex coordinated movement component and a rhythmic component, with only 15 percent of total studies included being eligible to be categorised for ‘both’ intervention elements. Rhythmic interventions were on average less than half in proportional representation (22 percent of total studies included) compared to complex coordinated movement interventions. A saturation of evidence was mapped for complex coordinated movement interventions (63 percent of total studies included) in comparison to other intervention categories across study design, participant ages, and outcome measures. The consistency of over-representation of complex coordinated movement interventions across all coding domains demonstrates a high research interest for the nature of these interventions. It may also speak to the methodological considerations for the resourcing and implementation of such interventions. Evidence presented the most common setting for interventions being an educational institution (72 percent of overall studies) and the use of either specially trained implementers (36 percent) or classroom teachers (33 percent) implementing the intervention across all intervention domains. Wide dissemination of intervention content was utilised approximately 90 percent of the time through face-to-face group modality. There was high homogeneity of the intervention methodology, where all three categories of interventions (rhythm, movement, or both) followed the same proportional representation of intervention setting, implementer, and modality.

The study design distribution across interventions was not equivalent, with the greatest number of randomised controlled trials, considered gold standard for experimental intervention effectiveness, being conducted with complex coordinated movement interventions. The greatest outcome of interest was evaluating executive functions, especially in primary aged children (ages 6 to 10), with the highest intervention domain represented as complex coordinated movement interventions. This was consistent across study design, where RCTs were broadly interested in evaluating executive function impact at a higher rate than other outcome measures, and this occurred at the greatest proportion with complex coordinated movement interventions.

The evidence for investigating any eligible intervention in children aged 0 to 2 years was sparse, with only four complex coordinated movement interventions conducted, and no rhythmic or interventions that included both. Although complex coordinated movement interventions may lack appropriateness for this age group, music, rhythm, or dance interventions may be more appropriate, with no research to date found in this age group. There was low representation of studies conducted with children at preschool age (3 to 5 years), with complex coordinated movement interventions being moderately higher than rhythmic interventions, followed by a sparsity of interventions that include both elements. Another area of disparity was in the pre-adolescent age group (11 to 12 years), where there was greater than twice the number of complex coordinated movement interventions to those that included both elements, and there was even greater sparsity of rhythmic interventions. Overall, early to middle childhood appeared to be the most frequently researched target population.

Outcomes demonstrated a dominant focus of executive function outcomes, with nearly 90 percent of studies measuring executive function effects, again highly represented by complex coordinated movement interventions, but with proportionally high representation of the other two intervention categories. Executive functions appeared to be of most interest in the primary school years, especially early primary school (ages 6 to 10 years). Although general cognition was of interest across studies (23 percent of total studies), it did not indicate the same significant trajectory across age or intervention domains. Finally, a clear gap (7 percent of total studies) was observed in the assessment of memory as an outcome, across both age and intervention domains. The outcome of focus across most studies (complex coordinated movement, rhythmic activities, or both) was on executive functioning. This is understandable, as these interventions are all proposing that enhancement of executive functioning occurs when engaging in complex intervention components that challenge and engage the prefrontal cortex mechanisms, specifically in relation to executive function control such as working memory, inhibitory control, and cognitive flexibility (Diamond, 2012; Diamond & Lee, 2011; Diamond & Ling, 2016, 2019; Willoughby & Hudson, 2023).

### Breadth and Coverage of the Current Literature and Guidelines for the Future

The presented Evidence and Gap Maps highlight two key areas of breadth and coverage of the literature: areas of saturation and areas of disparity. First, high saturation existed broadly for complex coordinated interventions, and specifically for RCTs for complex coordinated interventions that evaluated executive function outcomes in children in early primary school years. Overall, there were relatively few studies of interventions that included rhythmic interventions or combined element of both rhythmic and movement interventions that were evaluated using a RCT design. It is possible that discipline-related norms and capabilities have played a role over time in this context. For instance, physical activity interventions have arisen largely from the paediatric, psychological, and exercise science disciplines where there is a long history and strong capability and associated funding opportunities for RCT research. Rhythm-based interventions have largely arisen from music therapy and music education disciplines in which capability for RCT design and implementation is not as well embedded in discipline training (though RCTs are indeed conducted) and where research funding may be more scarce. To best advance this area, multi-disciplinary collaborations will be required.

In regard to populations studied, there were far fewer studies across intervention categories including young children (ages 0 to 2 years, and ages 3 to 5 years) compared to older children. While the suitability of rhythmic-based, dance-based, or movement-based interventions in this age group is high, selecting and implementing appropriate cognitive outcome measures for this age group can be challenging. Accessing this age group for research may also be more difficult in some settings, compared to once children have entered elementary/primary school, which is a largely universal experience in most jurisdictions.

Interventions in the rhythmic and both categories were relatively under-represented in the pre-adolescent (ages 11 to 12 years) population. Challenges with this age group include the selection of age-appropriate and developmentally appropriate rhythm activities at a time when children are transitioning from childhood to adolescence. Music preferences will vary widely in this age group, while body image and self-consciousness related to dance and other rhythmic movement are often high and may be anxiety-producing. It is possible that intervention designers and researchers have struggled to identify highly engaging rhythm-based activities for this age group. Pre-adolescence is a critical time to of malleability of key cognitive functions, such as cognitive flexibility, and interventions targeting such outcomes may yield greater long-term benefits for adolescence, where higher-order metacognition develops. Carefully designed group drumming activities that embed cognitive challenges may be one approach that could be better leveraged in this age group and that can avoid the challenges of repertoire selection and adolescent self-consciousness.

In regard to outcome measures, few interventions, across all intervention categories, assessed memory outcomes. This is somewhat surprising given many intervention studies focused on an element of memorisation embedded within the intervention. However, memory skills were not often directly assessed to better understand if these skills practiced within the intervention transferred to overall memorisation ability. It is possible that researchers did not expect or aim for transfer of this skill. Nevertheless, given memory skills are associated with broader desirable outcomes including academic achievement, this is an important area for future investigation.

An additional key area for consideration for future directions is the high representation of intervention studies conducted in educational settings (preschool, kindergarten, elementary school), where often the intervention implementor was a visiting trained professional, e.g. a Taekwondo intervention for elementary school children was taught by a Taekwondo Sensei, equipped with specialist skills and equipment. Overall, approximately 50 percent of implementers in studies across this review were trained specialised professionals visiting education settings purely for the purpose of intervention delivery, and thus there are clear limitations regarding the sustainability and scalability of such interventions within the classroom for long-term benefits. Key reviews (Diamond & Lee, 2011; Maynard et al., 2017; Ruhland, 2021) note the importance of sustainable intervention delivery in education settings, and emerging research is reflecting this shift (see Theroux et al., 2025) through designing and implementing interventions that can be delivered by generalist teachers as part of their regular practice. A key consideration for future research is to continue to develop interventions that successfully utilise a teacher delivery approach with specialist training and support (e.g. Williams et al., 2023), or a co-delivery approach which brings together generalist teachers and specialists in hybrid delivery, for example specialist-led video intervention components mediated by teachers (e.g. Theroux et al., 2025). These approaches will boost sustainability and scalability, ultimately ensuring that more children will have access to the potential benefits of these programs long term.

In summary, guidelines for future intervention design and research include the following:

1. Research Design: Consider the use of high-quality RCT study designs, or alternative rigorous designs, for interventions that include rhythmic elements, or intervention with both rhythmic and complex coordinated movement elements. Building multi-disciplinary research teams will support capability in this regard.
2. Intervention Implementation: When designing interventions for education settings (a dominant area of the literature), consider how implementation can be sustainable which may include embedding implementation within the curriculum, training teachers to implement, and creating a hybrid model of implementation that allows for specialist instruction without reliance on specialist delivery. Reduce the reliance on delivery and implementation that cannot be continued based on the resources and staffing at an education setting level.
3. Population: There is a clear need for increased research within key population groups, including all intervention designs for young children (ages 0 to 2 years, and ages 3 to 5 years) and specifically for rhythmic or ‘both’ interventions for pre-adolescent populations (ages 11 to 12 years). Consider how these populations may respond to the intended intervention to maximise engagement.
4. Outcomes: Memory was under-represented in the intervention outcomes which is misaligned with key intervention activities which often included memorisation components. Consider additional outcomes that may better represent the potential transferrable skills developed explicitly through the intervention. This may include memorisation as well as visual-spatial integration, sustained attention, and motor-based outcomes.
5. Comparison: The present review did not specifically evaluate the types of comparison groups included, and importantly the alternative treatment group comparisons. The EGM has categories of comparison groups coded to guide future research. Future research should carefully consider comparison groups (in addition to a waitlist control groups) to evaluate how alternative treatment comparisons provide insight into intervention gains, especially in the case of additional intervention elements (i.e. comparing rhythmic interventions to rhythmic + complex coordinated movement interventions) to understand the additive benefits.

Future reviews may wish to build upon the basic categorisation of interventions and break down the elements of interventions that have commonality (i.e. memorisation, changing of rules, cueing of actions). The further exploration of common mechanisms for change may allow for cross genre categorisation and coding to represent the commonalities between intervention categories, and any domains of interest to intersect with these, such as outcomes or participant characteristics that may highlight areas of saturation or disparity.

In summary, guidelines for future reviews include the following:

1. Code intervention sub-elements to allow head-to-head comparisons of interventions that specifically have the following components: memorisation (may subcategorise based on number of memorised actions or rules), action cuing, inhibition of habitual/primed response, synchronisation (which may also include social synchrony), object control or coordination, and the changing of rules.
2. Code outcome sub-categories that were all included within the ‘general cognition’ outcome group in the current review: attention, verbal and non-verbal reasoning, information processing, spatial processing, comprehension, and problem-solving.
3. Investigate and document participant age as a continuous variable within review data analysis, which can provide insight into effectiveness that categorical variables with an EGM cannot.

### Limitations and Future Directions

The current review has important limitations to consider when interpreting findings. While every effort was made to obtain all eligible full-text documents, the search returned a small number (1.94% of eligible studies for full-text screening) of studies that could not be accessed due to journal access limitations or publications in a language other than English (with no translation available). Thus, there is a subsection of the literature not able to be reviewed for eligibility, which future reviews should seek to address. For transparency, this list of studies is provided in supplementary materials.

Future reviews in this field should address aspects not covered under the EGM methodology taken here. Specifically, future reviews aimed at assessing intervention effectiveness should include a quality appraisal (e.g. risk of bias assessment) to document the degree of confidence that can be placed in study findings. Further, a future meta-analytic approach could calculate and synthesise effect sizes of the studies to allow for an estimate or overall effectiveness via meta-analysis or potentially comparison and ranking of different intervention categories via network meta-analysis (Hutton et al., 2015, 2016). Given the large number of studies, this study provides a high-level synthesis of evidence and gaps which could be used to inform more targeted synthesis of sub-domains that address quality evaluation and effect sizes.

## Conclusion

Overall, this review aimed to map the breadth of the literature comparing intervention categories of rhythm, complex coordinated movement, or a combination of both interventions across a range of methodological domains. Upon completion of evidence and gap mapping, a high representation of complex coordinated movement interventions was noted, followed by rhythmic interventions, and a highlighted disparity existing for interventions that include both elements. Reviews across these domains have previously grouped interventions based on intervention modality or participant characteristics, thus the present review provides a comprehensive picture of the current research literature and provides clear evidence to inform future research directions. Overall, researchers may base their own research interests from a wide range of mapped domains, citing clear lack of evidence for research across a range of study designs, age ranges, outcome categories, and intervention methodologies.

## Supplementary Information

Below is the link to the electronic supplementary material.

Supplementary file1 (DOCX 71 KB)

Supplementary file2 (DOCX 395 KB)

Supplementary file3 (DOCX 131 KB)

Supplementary file4 (DOCX 51 KB)

## Data Availability

No datasets were generated or analysed during the current study.

## References

[CR1] Alvarez-Bueno, C. P., Cavero-Redondo, Caterina, Sanchez-Lopez, Ivan, Martinez-Hortelano, Mairena, Martinez-Vizcaino, Jose Alberto, & Vicente. (2017). The effect of physical activity interventions on children’s cognition and metacognition: A systematic review and meta-analysis. *Journal of the American Academy of Child and Adolescent Psychiatry,**56*(9), 729–738. 10.1016/j.jaac.2017.06.012 28838577 10.1016/j.jaac.2017.06.012

[CR2] Alvarez-Bueno, C. P., Cavero-Redondo, Caterina, Sanchez-Lopez, Ivan, Pardo-Guijarro, Mairena, Martinez-Vizcaino, Maria Jesus, & Vicente. (2016). Association of physical activity with cognition, metacognition and academic performance in children and adolescents: A protocol for systematic review and meta-analysis. *BMJ Open,**6*(6), Article e011065. 10.1136/bmjopen-2016-011065 27354073 10.1136/bmjopen-2016-011065 PMC4932287

[CR3] Aranbarri, A., Aizpitarte, A., Arranz-Freijo, E., Fano, E., de Miguel, M. S., Stahmer, A. C., & Ibarluzea, J. M. (2023). What influences early cognitive development? Family context as a key mediator. *Journal of Applied Developmental Psychology*. 10.1016/j.appdev.2022.101480

[CR4] Barenberg, J., Berse, T., & Dutke, S. (2011). Executive functions in learning processes: Do they benefit from physical activity? *Educational Research Review,**6*(3), 208–222. 10.1016/j.edurev.2011.04.002

[CR5] Barnett, W. S. (2011). Effectiveness of early educational intervention. *Science,**333*(6045), 975–978. 10.1126/science.1204534 21852490 10.1126/science.1204534

[CR6] Bentley, L. A., Eager, R., Savage, S., Nielson, C., White, S. L., & Williams, K. E. (2023). A translational application of music for preschool cognitive development: RCT evidence for improved executive function, self-regulation, and school readiness. *Developmental Science,**26*(5), Article e13358. 10.1111/desc.13358 36511452 10.1111/desc.13358

[CR7] Best, J. R. (2010). Effects of physical activity on children’s executive function: Contributions of experimental research on aerobic exercise. *Developmental Review,**30*(4), 331–351. 10.1016/j.dr.2010.08.001 21818169 10.1016/j.dr.2010.08.001 PMC3147174

[CR8] Betts, J. L., Eggins, E., Chandler-Mather, N., Shelton, D., Till, H., Harnett, P., & Dawe, S. (2022). Interventions for improving executive functions in children with foetal alcohol spectrum disorder (FASD): A systematic review. *Campbell Systematic Reviews,**18*(4), Article e1258. 10.1002/cl2.1258 36908848 10.1002/cl2.1258 PMC9634003

[CR9] Biddle, S. J. H., Ciaccioni, S., Thomas, G., & Vergeer, I. (2019). Physical activity and mental health in children and adolescents: An updated review of reviews and an analysis of causality. *Psychology of Sport and Exercise,**42*, 146–155. 10.1016/j.psychsport.2018.08.011

[CR10] Birdee, G. S., Yeh, G. Y., Wayne, P. M., Phillips, R. S., Davis, R. B., & Gardiner, P. (2009). Clinical applications of yoga for the pediatric population: A systematic review. *Academic Pediatrics,**9*(4), 212-220. e219. 10.1016/j.acap.2009.04.002 19608122 10.1016/j.acap.2009.04.002 PMC2844096

[CR11] Camilli, G., Vargas, S., Ryan, S., & Barnett, W. S. (2010). Meta-analysis of the effects of early education interventions on cognitive and social development. *Teachers College Record,**112*(3), 579–620. 10.1177/016146811011200

[CR12] Cattan, S., Fitzsimons, E., Goodman, A., Phimister, A., Ploubidis, G. B., & Wertz, J. (2024). Early childhood inequalities. *Oxford Open Economics,**3*(S1), i711–i740. 10.1093/ooec/odad072

[CR13] Contreras-Osorio, F., Campos-Jara, C., Martinez-Salazar, C., Chirosa-Rios, L., & Martinez-Garcia, D. (2021). Effects of sport-based interventions on children’s executive function: A systematic review and meta-analysis. *Brain Sciences*. 10.3390/brainsci11060755 34200362 10.3390/brainsci11060755 PMC8226694

[CR14] Cooper, P. K. (2019). It’s all in your head: A meta-analysis on the effects of music training on cognitive measures in schoolchildren. *International Journal of Music Education,**38*(3), 321–336. 10.1177/0255761419881495

[CR15] Costa, M. T. S., Vieira, L. P., Barbosa, E. O., Mendes Oliveira, L., Maillot, P., Ottero Vaghetti, C. A., Giovani Carta, M., Machado, S., Gatica-Rojas, V., & Monteiro-Junior, R. S. (2019). Virtual reality-based exercise with exergames as medicine in different contexts: A short review. *Clinical Practice & Epidemiology in Mental Health,**15*, 15–20. 10.2174/1745017901915010015 30972138 10.2174/1745017901915010015 PMC6407662

[CR16] Degé, F., & Frischen, U. (2022). The impact of music training on executive functions in childhood—A systematic review. *Zeitschrift Für Erziehungswissenschaft,**25*(3), 579–602. 10.1007/s11618-022-01102-2

[CR17] Diamond, A. (2010). The evidence base for improving school outcomes by addressing the whole child and by addressing skills and attitudes, not just content. *Early Education Development,**21*(5), 780–793. 10.1080/10409289.2010.514522 21274420 10.1080/10409289.2010.514522 PMC3026344

[CR18] Diamond, A. (2012). Activities and programs that improve children’s executive functions. *Current Directions in Psychological Science,**21*(5), 335–341. 10.1177/0963721412453722 25328287 10.1177/0963721412453722 PMC4200392

[CR19] Diamond, A. (2015). Effects of physical exercise on executive functions: Going beyond simply moving to moving with thought. *Annals Sports and Medicine Research,**2*(1), 1011–1017. PMC4437637 26000340

[CR20] Diamond, A. (2016). Why improving and assessing executive functions early in life is critical. In J. A. Griffin, P. McCardle, & L. S. Freund (Eds.), *Executive function in preschool-age children: Integrating measurement, neurodevelopment, and translational research* (pp. 11–43). American Psychological Association.

[CR21] Diamond, A., & Lee, K. (2011). Interventions shown to aid executive function development in children 4 to 12 years old. *Science,**333*(6045), 959–964. 10.1126/science.1204529 21852486 10.1126/science.1204529 PMC3159917

[CR22] Diamond, A., & Ling, D. S. (2016). Conclusions about interventions, programs, and approaches for improving executive functions that appear justified and those that, despite much hype, do not. *Developmental Cognitive Neuroscience,**18*, 34–48. 10.1016/j.dcn.2015.11.005 26749076 10.1016/j.dcn.2015.11.005 PMC5108631

[CR23] Diamond, A., & Ling, D. S. (2019). Aerobic-exercise and resistance-training interventions have been among the least effective ways to improve executive functions of any method tried thus far. *Developmental Cognitive Neuroscience,**37*, Article 100572. 10.1016/j.dcn.2018.05.001 29909061 10.1016/j.dcn.2018.05.001 PMC6969311

[CR25] dos Santos, E. S. L., de Kieviet, J. F., Königs, M., van Elburg, R. M., & Oosterlaan, J. (2013). Predictive value of the Bayley scales of infant development on development of very preterm/very low birth weight children: A meta-analysis. *Early Human Development,**89*(7), 487–496. 10.1016/j.earlhumdev.2013.03.008 23597678 10.1016/j.earlhumdev.2013.03.008

[CR26] Eggins, E., Mazerolle, L., Higginson, A., Hine, L., Walsh, K., Sydes, M., McEwan, J., Hassall, G., Roetman, S., & Wallis, R. (2021). Criminal justice responses to child sexual abuse material offending: A systematic review and evidence and gap map. *Trends and Issues in Crime and Criminal Justice,**623*, 1–18.

[CR27] Eggins, E., Wilson, D. B., Betts, J., Roetman, S., Chandler-Mather, N., Theroux, B., & Dawe, S. (2024). Psychosocial, pharmacological, and legal interventions for improving the psychosocial outcomes of children with substance misusing parents: A systematic review. *Campbell Systematic Reviews,**20*(3), Article e1413. 10.1002/cl2.1413 10.1002/cl2.1113 PMC8356279 37131914

[CR28] Fedewa, A. L., & Ahn, S. (2011). The effects of physical activity and physical fitness on children’s achievement and cognitive outcomes: A meta-analysis. *Research Quarterly for Exercise and Sport,**82*(3), 521–535. 10.1080/02701367.2011.10599785 21957711 10.1080/02701367.2011.10599785

[CR29] Ferreira-Vorkapic, C., Feitoza, J. M., Marchioro, M., Simoes, J., Kozasa, E., & Telles, S. (2015). Are there benefits from teaching yoga at schools? A systematic review of randomized control trials of yoga-based interventions. *Evidence-Based Complementary and Alternative Medicine,**2015*, Article 345835. 10.1155/2015/345835 26491461 10.1155/2015/345835 PMC4600929

[CR30] Filipe, M. G., Magalhaes, S., Veloso, A. S., Costa, A. F., Ribeiro, L., Araujo, P., Castro, S. L., & Limpo, T. (2021). Exploring the effects of meditation techniques used by mindfulness-based programs on the cognitive, social-emotional, and academic skills of children: A systematic review. *Frontiers in Psychology,**12*, Article 660650. 10.3389/fpsyg.2021.660650 34867573 10.3389/fpsyg.2021.660650 PMC8632731

[CR31] Finch, M., Featherston, R., Chakraborty, S., Bjørndal, L., Mildon, R., Albers, B., Fiennes, C., Taylor, D. J., Schachtman, R., & Yang, T. (2021). Interventions that address institutional child maltreatment: An evidence and gap map. *Campbell Systematic Reviews,**17*(1), Article e1139. 10.1002/cl2.1139 37133265 10.1002/cl2.1139 PMC8356353

[CR32] Fitzpatrick, C., Archambault, I., Janosz, M., & Pagani, L. (2015). Early childhood working memory forecasts high school dropout risk. *Intelligence,**53*, 160–165. 10.1016/j.intell.2015.10.002

[CR33] Friedman, Z. L., Ochoa, J., Prisco, D., & Seruya, F. M. (2023). Connected rhythm: A scoping review of therapeutic drumming as an intervention for autistic individuals. *The Open Journal of Occupational Therapy,**11*(4), 1–17. 10.15453/2168-6408.2133

[CR34] Frischen, U., Schwarzer, G., & Dege, F. (2019). Comparing the effects of rhythm-based music training and pitch-based music training on executive functions in preschoolers. *Frontiers in Integrative Neuroscience*. 10.3389/fnint.2019.00041 31507385 10.3389/fnint.2019.00041 PMC6718722

[CR35] Grande, A. J., Hoffmann, M. S., Evans-Lacko, S., Ziebold, C., de Miranda, C. T., McDaid, D., Tomasi, C., & Ribeiro, W. S. (2022). Efficacy of school-based interventions for mental health problems in children and adolescents in low and middle-income countries: A systematic review and meta-analysis. *Frontiers in Psychiatry,**13*, 1012257. 10.3389/fpsyt.2022.1012257 36684024 10.3389/fpsyt.2022.1012257 PMC9852982

[CR36] Greco, G., & De Ronzi, R. (2020). Effect of karate training on social, emotional, and executive functioning in children with autism spectrum disorder. *Journal of Physical Education & Sport,**20*(4), 1637–1645. 10.7752/jpes.2020.04223

[CR37] Hamel, C., Michaud, A., Thuku, M., Affengruber, L., Skidmore, B., Nussbaumer-Streit, B., Stevens, A., & Garritty, C. (2020). Few evaluative studies exist examining rapid review methodology across stages of conduct: A systematic scoping review. *Journal of Clinical Epidemiology,**126*, 131–140. 10.1016/j.jclinepi.2020.06.027 32599023 10.1016/j.jclinepi.2020.06.027

[CR38] Higgins, J. P., López-López, J. A., Becker, B. J., Davies, S. R., Dawson, S., Grimshaw, J. M., McGuinness, L. A., Moore, T. H., Rehfuess, E. A., & Thomas, J. (2019). Synthesising quantitative evidence in systematic reviews of complex health interventions. *BMJ Global Health,**4*(Suppl 1), Article e000858. 10.1136/bmjgh-2018-000858 30775014 10.1136/bmjgh-2018-000858 PMC6350707

[CR39] Hutton, B., Catala-Lopez, F., & Moher, D. (2016). The PRISMA statement extension for systematic reviews incorporating network meta-analysis: PRISMA-NMA. *Medicina Clínica (English Edition),**147*(6), 262–266. 10.1016/j.medcle.2016.10.003 10.1016/j.medcli.2016.02.025 27040178

[CR40] Hutton, B., Salanti, G., Caldwell, D. M., Chaimani, A., Schmid, C. H., Cameron, C., Ioannidis, J. P., Straus, S., Thorlund, K., & Jansen, J. P. (2015). The PRISMA extension statement for reporting of systematic reviews incorporating network meta-analyses of health care interventions: Checklist and explanations. *Annals of Internal Medicine,**162*(11), 777–784. 10.7326/M14-2385 26030634 10.7326/M14-2385

[CR41] Jamey, K., Foster, N., Hyde, K., & Bella, S. (2023). Does music training improve inhibition control in children? A systematic review and meta-analysis. *bioRxiv*. 10.1101/2023.02.08.527718 10.1016/j.cognition.2024.105913 39197250

[CR42] Jaschke, A. C., Eggermont, L. H., Honing, H., & Scherder, E. J. (2013). Music education and its effect on intellectual abilities in children: A systematic review. *Reviews in the Neurosciences,**24*(6), 665–675. 10.1515/revneuro-2013-0023 24169311 10.1515/revneuro-2013-0023

[CR43] Jeong, J., Franchett, E. E., Ramos de Oliveira, C. V., Rehmani, K., & Yousafzai, A. K. (2021). Parenting interventions to promote early child development in the first three years of life: A global systematic review and meta-analysis. *PLoS Medicine,**18*(5), Article e1003602. 10.1371/journal.pmed.1003602 33970913 10.1371/journal.pmed.1003602 PMC8109838

[CR44] Keeley, T. J., & Fox, K. R. (2009). The impact of physical activity and fitness on academic achievement and cognitive performance in children. *International Review of Sport and Exercise Psychology,**2*(2), 198–214. 10.1080/17509840903233822

[CR45] Khalsa, S. B., & Butzer, B. (2016). Yoga in school settings: A research review. *Annals of the New York Academy of Sciences,**1373*(1), 45–55. 10.1111/nyas.13025 26919395 10.1111/nyas.13025

[CR46] Lakes, K. D. (2013). Exploring new synergies between physical activity and cognitive/academic-related performance—Effects of school-based martial arts training on children’s cognitive and affective functioning. *Journal of Sport & Exercise Psychology,**35*, S9–S10.

[CR103] Larson, K., Russ, S. A., Nelson, B. B., Olson, L. M., & Halfon, N. (2015). Cognitive ability at kindergarten entry and socioeconomic status. *Pediatrics*, *135*(2), e440–e448. 10.1542/peds.2014-0434 25601983 10.1542/peds.2014-0434

[CR47] Lakes, K. D., Bryars, T., Sirisinahal, S., Salim, N., Arastoo, S., Emmerson, N., Kang, D., Shim, L., Wong, D., & Kang, C. J. (2013). The healthy for life taekwondo pilot study: A preliminary evaluation of effects on executive function and BMI, feasibility, and acceptability. *Mental Health and Physical Activity,**6*(3), 181–188. 10.1016/j.mhpa.2013.07.002 24563664 10.1016/j.mhpa.2013.07.002 PMC3927879

[CR48] Lakes, K. D., & Hoyt, W. I. (2004). Promoting self-regulation through school-based martial arts training. *Journal of Applied Developmental Psychology,**25*(3), 283–302. 10.1016/j.appdev.2004.04.002

[CR49] Laurent, C. W. S., Burkart, S., Andre, C., & Spencer, R. M. (2021). Physical activity, fitness, school readiness, and cognition in early childhood: A systematic review. *Journal of Physical Activity & Health,**18*(8), 1004–1013. 10.1123/jpah.2020-0844 34140418 10.1123/jpah.2020-0844 PMC9297301

[CR104] Letourneau, N. L., Duffett-Leger, L., Levac, L., Watson, B., & Young-Morris, C. (2013). Socioeconomic status and child development: A meta-analysis. *Journal of Emotional and Behavioral Disorders*, *21*(3), 211–224. 10.1177/1063426611421007

[CR50] Lobo, Y., & Winsler, A. (2006). The effects of a creative dance and movement program on the social competence of head start preschoolers. *Social Development,**15*(3), 501–519. 10.1111/j.1467-9507.2006.00353.x

[CR51] Mak, C., Whittingham, K., Cunningham, R., & Boyd, R. (2018). Efficacy of mindfulness-based interventions for attention and executive function in children and adolescents—A systematic review. *Mindfulness,**9*, 59–78. 10.1007/s12671-017-0770-6

[CR52] Maresca, G., Portaro, S., Naro, A., Crisafulli, R., Raffa, A., Scarcella, I., Aliberti, B., Gemelli, G., & Calabro, R. S. (2022). Hippotherapy in neurodevelopmental disorders: A narrative review focusing on cognitive and behavioral outcomes. *Applied Neuropsychology of Children,**11*(3), 553–560. 10.1080/21622965.2020.1852084 10.1080/21622965.2020.1852084 33949903

[CR53] Masini, A., Marini, S., Gori, D., Leoni, E., Rochira, A., & Dallolio, L. (2020). Evaluation of school-based interventions of active breaks in primary schools: A systematic review and meta-analysis. *Journal of Science and Medicine in Sport,**23*(4), 377–384. 10.1016/j.jsams.2019.10.008 31722840 10.1016/j.jsams.2019.10.008

[CR54] Mavilidi, M. F., Pesce, C., Benzing, V., Schmidt, M., Paas, F., Okely, A. D., & Vazou, S. (2022). Meta-analysis of movement-based interventions to aid academic and behavioral outcomes: A taxonomy of relevance and integration. *Educational Research Review*. 10.1016/j.edurev.2022.100478

[CR55] Mayer-Benarous, H., Benarous, X., Vonthron, F., & Cohen, D. (2021). Music therapy for children with autistic spectrum disorder and/or other neurodevelopmental disorders: A systematic review. *Frontiers in Psychiatry,**12*, Article 643234. 10.3389/fpsyt.2021.643234 33897497 10.3389/fpsyt.2021.643234 PMC8062803

[CR56] Maynard, B. R., Solis, M., Miller, V., & Brendel, K. E. (2017). Mindfulness-based intervenions for improving cogniion, academic achievement, behavior, and socioemoional funcioning of primary and secondary school students. *The Campbell Collaboration*. 10.4073/csr.2017.5

[CR57] McLaughlin, K. A., Sheridan, M. A., & Nelson, C. (2013). Adverse childhood experiences and brain development: Neurobiological mechanisms linking the social environment to psychiatric disorders. *A Life Course Approach to Mental Disorders*, pp 249–258.

[CR58] Miendlarzewska, E. A., & Trost, W. J. (2013). How musical training affects cognitive development: Rhythm, reward and other modulating variables. *Frontiers in Neuroscience,**7*, 279. 10.3389/fnins.2013.00279 24672420 10.3389/fnins.2013.00279 PMC3957486

[CR105] Najman, J. M., Aird, R., Bor, W., O’Callaghan, M., Williams, G. M., & Shuttlewood, G. J. (2004). The generational transmission of socioeconomic inequalities in child cognitive development and emotional health. *Social Science & Medicine*, *58*(6), 1147–1158. 10.1016/S0277-9536(03)00286-7 14723909 10.1016/s0277-9536(03)00286-7

[CR59] Page, M. J., McKenzie, J. E., Bossuyt, P. M., Boutron, I., Hoffmann, T. C., Mulrow, C. D., Shamseer, L., Tetzlaff, J. M., Akl, E. A., & Brennan, S. E. (2021). The PRISMA 2020 statement: An updated guideline for reporting systematic reviews. *BMJ*. 10.1136/bmj.n71 33782057 10.1136/bmj.n71 PMC8005924

[CR60] Pandey, A., Hale, D., Das, S., Goddings, A. L., Blakemore, S. J., & Viner, R. M. (2018). Effectiveness of universal self-regulation-based interventions in children and adolescents: A systematic review and meta-analysis. *JAMA Pediatrics,**172*(6), 566–575. 10.1001/jamapediatrics.2018.0232 29710097 10.1001/jamapediatrics.2018.0232 PMC6059379

[CR61] Pesce, C., Crova, C., Cereatti, L., Casella, R., & Bellucci, M. (2009). Physical activity and mental performance in preadolescents: Effects of acute exercise on free-recall memory. *Mental Health and Physical Activity,**2*(1), 16–22. 10.1016/j.mhpa.2009.02.001

[CR62] Pesce, C., Crova, C., Marchetti, R., Struzzolino, I., Masci, I., Vannozzi, G., & Forte, R. (2013). Searching for cognitively optimal challenge point in physical activity for children with typical and atypical motor development. *Mental Health and Physical Activity,**6*(3), 172–180. 10.1016/j.mhpa.2013.07.001

[CR63] Pesce, C., Vazou, S., Benzing, V., Álvarez-Bueno, C., Anzeneder, S., Mavilidi, M. F., Leone, L., & Schmidt, M. (2023). Effects of chronic physical activity on cognition across the lifespan: A systematic meta-review of randomized controlled trials and realist synthesis of contextualized mechanisms. *International Review of Sport and Exercise Psychology,**16*(1), 722–760. 10.1080/1750984X.2021.1929404

[CR64] Peters, V., Bissonnette, J., Nadeau, D., Gauthier-Legare, A., & Noel, M. A. (2024). The impact of musicking on emotion regulation: A systematic review and meta-analysis. *Psychology of Music,**52*(5), 548–568. 10.1177/03057356231212362 39297022 10.1177/03057356231212362 PMC11405141

[CR65] Petrigna, L., Thomas, E., Brusa, J., Rizzo, F., Scardina, A., Galassi, C., Lo Verde, D., Caramazza, G., & Bellafiore, M. (2022). Does learning through movement improve academic performance in primary schoolchildren? A systematic review. *Frontiers in Pediatrics,**10*, Article 841582. 10.3389/fped.2022.841582 35345611 10.3389/fped.2022.841582 PMC8957225

[CR66] Pickerell, L. E., Pennington, K., Cartledge, C., Miller, K. A., & Curtis, F. (2023). The effectiveness of school-based mindfulness and cognitive behavioural programmes to improve emotional regulation in 7–12-year-olds: A systematic review and meta-analysis. *Mindfulness,**14*(5), 1068–1087. 10.1007/s12671-023-02131-6

[CR67] Pundir, P., Saran, A., White, H., Subrahmanian, R., & Adona, J. (2020). Interventions for reducing violence against children in low-and middle-income countries: An evidence and gap map. *Campbell Systematic Reviews,**16*(4), Article e1120. 10.1002/cl2.1120 37016609 10.1002/cl2.1120 PMC8356324

[CR68] Roberts, M., Tolar-Peterson, T., Reynolds, A., Wall, C., Reeder, N., & Rico Mendez, G. (2022). The effects of nutritional interventions on the cognitive development of preschool-age children: A systematic review. *Nutrients*. 10.3390/nu14030532 35276891 10.3390/nu14030532 PMC8839299

[CR69] Rodwin, A. H., Shimizu, R., Travis, R., Jr., James, K. J., Banya, M., & Munson, M. R. (2022). A systematic review of music-based interventions to improve treatment engagement and mental health outcomes for adolescents and young adults. *Child and Adolescent Social Work Journal*. 10.1007/s10560-022-00893-x 36407676 10.1007/s10560-022-00893-x PMC9666939

[CR70] Román-Caballero, R., Vadillo, M. A., Trainor, L. J., & Lupiáñez, J. (2022). Please don’t stop the music: A meta-analysis of the cognitive and academic benefits of instrumental musical training in childhood and adolescence. *Educational Research Review*. 10.1016/j.edurev.2022.100436

[CR71] Ruhland, S. L., & K. W. (2021). Effect of classroom-based physical activity interventions on attention and on-task behavior in schoolchildren: A systematic review. *Sports Medicine and Health Science*. 10.1016/j.smhs.2021.08.003 35784522 10.1016/j.smhs.2021.08.003 PMC9219312

[CR72] Sala, G., & Gobet, F. (2017). When the music’s over. Does music skill transfer to children’s and young adolescents’ cognitive and academic skills? A meta-analysis. *Educational Research Review,**20*, 55–67. 10.1016/j.edurev.2016.11.005

[CR73] Sancassiani, F., Pintus, E., Holte, A., Paulus, P., Moro, M. F., Cossu, G., Angermeyer, M. C., Carta, M. G., & Lindert, J. (2015). Enhancing the emotional and social skills of the youth to promote their wellbeing and positive development: A systematic review of universal school-based randomized controlled trials. *Clinical Practice and Epidemiology in Mental Health: CP & EMH,**11*(Suppl 1 M2), Article 21. 10.2174/1745017901511010021 25834626 10.2174/1745017901511010021 PMC4378066

[CR74] Sarma, K. M., Carthy, S. L., & Cox, K. M. (2022). Mental disorder, psychological problems and terrorist behaviour: A systematic review and meta-analysis. *Campbell Systematic Reviews,**18*(3), Article e1268. 10.1002/cl2.1268 36913225 10.1002/cl2.1268 PMC9364674

[CR75] Schuman-Olivier, Z., Trombka, M., Lovas, D. A., Brewer, J. A., Vago, D. R., Gawande, R., Dunne, J. P., Lazar, S. W., Loucks, E. B., & Fulwiler, C. (2020). Mindfulness and behavior change. *Harvard Review of Psychiatry,**28*(6), 371–394. 10.1097/HRP.0000000000000277 33156156 10.1097/HRP.0000000000000277 PMC7647439

[CR76] Shen, Y., Zhao, Q., Huang, Y., Liu, G., & Fang, L. L. (2020). Promotion of street-dance training on the executive function in preschool children. *Frontiers in Psychology*. 10.3389/fpsyg.2020.585598 33192915 10.3389/fpsyg.2020.585598 PMC7642602

[CR77] Sibley, B. A., & Etnier, J. L. (2003). The relationship between physical activity and cognition in children: A meta-analysis. *Pediatric Exercise Science,**15*(3), 243–256. 10.1123/pes.15.3.243

[CR78] Snilstveit, B., Vojtkova, M., Bhavsar, A., Stevenson, J., & Gaarder, M. (2016). Evidence & gap maps: A tool for promoting evidence informed policy and strategic research agendas. *Journal of Clinical Epidemiology,**79*, 120–129. 10.1016/j.jclinepi.2016.05.015 27387966 10.1016/j.jclinepi.2016.05.015

[CR79] Song, W., Feng, L., Wang, J., Ma, F., Chen, J., Qu, S., & Luo, D. (2022). Play smart, be smart? Effect of cognitively engaging physical activity interventions on executive function among children 4–12 years old: A systematic review and meta-analysis. *Brain Sciences*. 10.3390/brainsci12060762 35741648 10.3390/brainsci12060762 PMC9220861

[CR80] Sousa Junior, R. R., Souto, D. O., Camargos, A. C. R., Clutterbuck, G. L., & Leite, H. R. (2023). Moving together is better: A systematic review with meta-analysis of sports-focused interventions aiming to improve physical activity participation in children and adolescents with cerebral palsy. *Disability Rehabilitation,**45*(15), 2398–2408. 10.1080/09638288.2022.2098394 35853235 10.1080/09638288.2022.2098394

[CR81] Sydes, M., Hine, L., Higginson, A., McEwan, J., Dugan, L., & Mazerolle, L. (2023). Criminal justice interventions for preventing radicalisation, violent extremism and terrorism: An evidence and gap map. *Campbell Systematic Reviews,**19*(4), Article e1366. 10.1002/cl2.1366 38024779 10.1002/cl2.1366 PMC10644945

[CR82] Thaut, M. (2013). Entrainment and the motor system. *Music Therapy Perspectives*. 10.1093/mtp/31.1.31

[CR83] Thaut, M. H., McIntosh, G. C., & Hoemberg, V. (2014). Neurobiological foundations of neurologic music therapy: Rhythmic entrainment and the motor system. *Frontiers in Psychology,**5*, 1185. 10.3389/fpsyg.2014.01185 25774137 10.3389/fpsyg.2014.01185 PMC4344110

[CR84] Theroux, B. M., Chandler-Mather, N., Paynter, J., Dawe, S., & Williams, K. E. (2025). The mindful movement program in primary schools: A single-arm pilot intervention study. *BMC Psychology,**13*(1), 1–16. 10.1186/s40359-025-02689-x 40312339 10.1186/s40359-025-02689-x PMC12044745

[CR85] Thomas, J., Graziosi, S., Brunton, J., Ghouze, Z., O'Driscoll, P., Bond, M., & Koryakina, A. (2023). *EPPI reviewer: Advanced software for systematic reviews, maps and evidence synthesis*. In *EPPI Centre Software* UCL Social Research Institute. https://eppi.ioe.ac.uk/cms/Default.aspx?alias=eppi.ioe.ac.uk/cms/er4

[CR86] Tomporowski, P. D., McCullick, B. A., & Pesce, C. (2015). *Enhancing children's cognition with physical activity games*. Human Kinetics.

[CR87] Tomporowski, P. D., & Pesce, C. (2019). Exercise, sports, and performance arts benefit cognition via a common process. *Psychological Bulletin,**145*(9), 929–951. 10.1037/bul0000200 31192623 10.1037/bul0000200

[CR88] Treurnicht, N. K., Kingsnorth, S., Lamont, A., McKeever, P., & Macarthur, C. (2011). The effectiveness of music in pediatric healthcare: A systematic review of randomized controlled trials. *Evidence Based Complementary Alternative Medicine,**2011*, Article 464759. 10.1155/2011/464759 20976017 10.1155/2011/464759 PMC2957635

[CR89] Tudor, K., Maloney, S., Raja, A., Baer, R., Blakemore, S. J., Byford, S., Crane, C., Dalgleish, T., De Wilde, K., Ford, T., Greenberg, M., Hinze, V., Lord, L., Radley, L., Opaleye, E. S., Taylor, L., Ukoumunne, O. C., Viner, R., Team, M.,…Montero-Marin, J. (2022). Universal mindfulness training in schools for adolescents: A scoping review and conceptual model of moderators, mediators, and implementation factors. *Prevention Science,**23*(6), 934–953. 10.1007/s11121-022-01361-9 35267177 10.1007/s11121-022-01361-9 PMC9343282

[CR90] van der Fels, I. M., Te Wierike, S. C., Hartman, E., Elferink-Gemser, M. T., Smith, J., & Visscher, C. (2015). The relationship between motor skills and cognitive skills in 4–16 year old typically developing children: A systematic review. *Journal of Science and Medicine in Sport,**18*(6), 697–703. 10.1016/j.jsams.2014.09.007 25311901 10.1016/j.jsams.2014.09.007

[CR91] Vazou, S., Pesce, C., Lakes, K., & Smiley-Oyen, A. (2019). More than one road leads to Rome: A narrative review and meta-analysis of physical activity intervention effects on cognition in youth. *International Journal of Sport Exercice Psychology,**17*(2), 153–178. 10.1080/1612197X.2016.1223423 10.1080/1612197X.2016.1223423 PMC6615761 31289454

[CR92] Weaver, L. L., & Darragh, A. R. (2015). Systematic review of yoga interventions for anxiety reduction among children and adolescents. *American Journal of Occupational Therapy*. 10.5014/ajot.2015.020115 10.5014/ajot.2015.020115 26565100

[CR93] White, H., Albers, B., Gaarder, M., Kornør, H., Littell, J., Marshall, Z., Mathew, C., Pigott, T., Snilstveit, B., & Waddington, H. (2020). Guidance for producing a campbell evidence and gap map. *Campbell Systematic Reviews,**16*(4), Article e1125. 10.1002/cl2.1125 37016607 10.1002/cl2.1125 PMC8356343

[CR94] White, H., Saran, A., Verma, A., Oprea, E., & Babudu, P. (2021). Evidence and gap map of interventions to prevent children getting involved in violence. https://youthendowmentfund.org.uk/wp-content/uploads/2021/02/YEF-Evidence-and-Gap-Map-Technical-Report-FINAL.pdf

[CR95] Willemin, T., Litchke, L. G., Liu, T., & Ekins, C. (2018). Social emotional effects of drumtastic®: A dyadic within-group drumming pilot program for children with autism spectrum disorder. *International Journal of Special Education,**33*(1), 94–103.

[CR96] Williams, K. E., Bentley, L. A., Savage, S., Eager, R., & Nielson, C. (2023). Rhythm and movement delivered by teachers supports self-regulation skills of preschool-aged children in disadvantaged communities: A clustered RCT. *Early Childhood Research Quarterly,**65*, 115–128. 10.1016/j.ecresq.2023.05.008

[CR97] Williams, K. E., & Berthelsen, D. (2019). Implementation of a rhythm and movement intervention to support self-regulation skills of preschool-aged children in disadvantaged communities. *Psychology of Music,**47*(6), 800–820. 10.1177/0305735619861433

[CR98] Willoughby, M. T., & Hudson, K. (2023). Contributions of motor skill development and physical activity to the ontogeny of executive function skills in early childhood. *Developmental Review,**70*, Article 101102. 10.1016/j.dr.2023.101102

[CR99] Xiao, N., Shinwari, K., Kiselev, S., Huang, X., Li, B., & Qi, J. (2023). Effects of equine-assisted activities and therapies for individuals with autism spectrum disorder: Systematic review and meta-analysis. *International Journal of Environmental Research and Public Health*. 10.3390/ijerph20032630 36767996 10.3390/ijerph20032630 PMC9915993

[CR100] Xue, Y., Yang, Y., & Huang, T. (2019). Effects of chronic exercise interventions on executive function among children and adolescents: A systematic review with meta-analysis. *British Journal of Sports Medicine,**53*(22), 1397–1404. 10.1136/bjsports-2018-099825 30737201 10.1136/bjsports-2018-099825

[CR101] Yoo, G. E., & Kim, S. J. (2018). Dyadic drum playing and social skills: Implications for rhythm-mediated intervention for children with autism spectrum disorder. *Journal of Music Therapy,**55*(3), 340–375. 10.1093/jmt/thy013 30137544 10.1093/jmt/thy013

[CR102] Zenner, C., Herrnleben-Kurz, S., & Walach, H. (2014). Mindfulness-based interventions in schools: A systematic review and meta-analysis. *Frontiers in Psychology,**5*, Article 603. 10.3389/fpsyg.2014.00603 25071620 10.3389/fpsyg.2014.00603 PMC4075476

