# Supplementary material for: A Systematic Review and Evidence Gap Map Evaluation of Rhythmic and/or Complex Movement Interventions and Child Cognitive Outcomes
Source: Clin Child Fam Psychol Rev. 2025 Oct 11;28(4):912–29. doi: 10.1007/s10567-025-00547-1 (PMC12660398; doi:10.1007/s10567-025-00547-1)
Supplement: Supplementary file 1 — Supplementary file1 (DOCX 71 KB) [file 10567_2025_547_MOESM1_ESM.docx]

# Supplementary Materials A - Tables

Table A1. Generic Search Strategy

| Search No. | Fields | Syntax |
| --- | --- | --- |
| 1 | Title, Abstract, Subject, Author Supplied Keywords | ("comparison condition*" OR "comparison group*" OR "control condition*" OR "control group*" OR evaluat* OR experiment* OR interven* OR match* OR meta-analy* OR pilot* OR placebo* OR program* OR "propensity score*" OR random* OR RCT OR review* OR therap* OR train* OR treat* OR trial*) |
| 2 | Title, Abstract, Subject, Author Supplied Keywords | (physical OR active OR activity OR exercise* OR move* OR sport* OR action* OR bilateral OR proprioception OR danc* OR martial) |
| 3 | Title, Abstract, Subject, Author Supplied Keywords | cogniti* adj5 (child* OR infan* OR student* OR toddler* OR "pre-adolescen*" OR youth OR adolescen* OR preschool* OR kinder*) |
| 4 | Title, Abstract, Subject, Author Supplied Keywords | inhibit* adj5 (child* OR infan* OR student* OR toddler* OR "pre-adolescen*" OR youth OR adolescen* OR preschool* OR kinder*) |
| 5 | Title, Abstract, Subject, Author Supplied Keywords | memory adj5 (child* OR infan* OR student* OR toddler* OR "pre-adolescen*" OR youth OR adolescen* OR preschool* OR kinder*) |
| 6 | Title, Abstract, Subject, Author Supplied Keywords | attention adj5 (child* OR infan* OR student* OR toddler* OR "pre-adolescen*" OR youth OR adolescen* OR preschool* OR kinder*) |
| 7 | Title, Abstract, Subject, Author Supplied Keywords | spatial adj5 (child* OR infan* OR student* OR toddler* OR "pre-adolescen*" OR youth OR adolescen* OR preschool* OR kinder*) |
| 8 | Title, Abstract, Subject, Author Supplied Keywords | receptive adj5 (child* OR infan* OR student* OR toddler* OR "pre-adolescen*" OR youth OR adolescen* OR preschool* OR kinder*) |
| 9 | Title, Abstract, Subject, Author Supplied Keywords | expressive adj5 (child* OR infan* OR student* OR toddler* OR "pre-adolescen*" OR youth OR adolescen* OR preschool* OR kinder*) |
| 10 | Title, Abstract, Subject, Author Supplied Keywords | reasoning adj5 (child* OR infan* OR student* OR toddler* OR "pre-adolescen*" OR youth OR adolescen* OR preschool* OR kinder*) |
| 11 | Title, Abstract, Subject, Author Supplied Keywords | processing adj5 (child* OR infan* OR student* OR toddler* OR "pre-adolescen*" OR youth OR adolescen* OR preschool* OR kinder*) |
| 12 | Title, Abstract, Subject, Author Supplied Keywords | "problem-solv*" adj5 (child* OR infan* OR student* OR toddler* OR "pre-adolescen*" OR youth OR adolescen* OR preschool* OR kinder*) |
| 13 | Title, Abstract, Subject, Author Supplied Keywords | "problem solv*" adj5 (child* OR infan* OR student* OR toddler* OR "pre-adolescen*" OR youth OR adolescen* OR preschool* OR kinder*) |
| 14 | Title, Abstract, Subject, Author Supplied Keywords | "executive function*" adj5 (child* OR infan* OR student* OR toddler* OR "pre-adolescen*" OR youth OR adolescen* OR preschool* OR kinder*) |
| 15 | Title, Abstract, Subject, Author Supplied Keywords | 3 OR 4 OR 5 OR 6 OR 7 OR 8 OR 9 OR 10 OR 11 OR 12 OR 13 OR 14 |
| 16 | Title, Abstract, Subject, Author Supplied Keywords | 1 AND 2 AND 15 |
| 17 | Title, Abstract, Subject, Author Supplied Keywords | "comparison condition*" OR "comparison group*" OR "control condition*" OR "control group*" OR evaluat* OR experiment* OR interven* OR match* OR meta-analy* OR pilot* OR placebo* OR program* OR "propensity score*" OR random* OR RCT OR review* OR therap* OR train* OR treat* OR trial* |
| 18 | Title, Abstract, Subject, Author Supplied Keywords | rhythm* OR music* OR beat* OR synchroni* OR entrain* |
| 19 | Title, Abstract, Subject, Author Supplied Keywords | cogniti* adj5 (child* OR infan* OR student* OR toddler* OR "pre-adolescen*" OR youth OR adolescen* OR preschool* OR kinder*) |
| 20 | Title, Abstract, Subject, Author Supplied Keywords | inhibit* adj5 (child* OR infan* OR student* OR toddler* OR "pre-adolescen*" OR youth OR adolescen* OR preschool* OR kinder*) |
| 21 | Title, Abstract, Subject, Author Supplied Keywords | memory adj5 (child* OR infan* OR student* OR toddler* OR "pre-adolescen*" OR youth OR adolescen* OR preschool* OR kinder*) |
| 22 | Title, Abstract, Subject, Author Supplied Keywords | attention adj5 (child* OR infan* OR student* OR toddler* OR "pre-adolescen*" OR youth OR adolescen* OR preschool* OR kinder*) |
| 23 | Title, Abstract, Subject, Author Supplied Keywords | spatial adj5 (child* OR infan* OR student* OR toddler* OR "pre-adolescen*" OR youth OR adolescen* OR preschool* OR kinder*) |
| 24 | Title, Abstract, Subject, Author Supplied Keywords | receptive adj5 (child* OR infan* OR student* OR toddler* OR "pre-adolescen*" OR youth OR adolescen* OR preschool* OR kinder*) |
| 25 | Title, Abstract, Subject, Author Supplied Keywords | expressive adj5 (child* OR infan* OR student* OR toddler* OR "pre-adolescen*" OR youth OR adolescen* OR preschool* OR kinder*) |
| 26 | Title, Abstract, Subject, Author Supplied Keywords | reasoning adj5 (child* OR infan* OR student* OR toddler* OR "pre-adolescen*" OR youth OR adolescen* OR preschool* OR kinder*) |
| 27 | Title, Abstract, Subject, Author Supplied Keywords | processing adj5 (child* OR infan* OR student* OR toddler* OR "pre-adolescen*" OR youth OR adolescen* OR preschool* OR kinder*) |
| 28 | Title, Abstract, Subject, Author Supplied Keywords | "problem-solv*" adj5 (child* OR infan* OR student* OR toddler* OR "pre-adolescen*" OR youth OR adolescen* OR preschool* OR kinder*) |
| 29 | Title, Abstract, Subject, Author Supplied Keywords | "problem solv*" adj5 (child* OR infan* OR student* OR toddler* OR "pre-adolescen*" OR youth OR adolescen* OR preschool* OR kinder*) |
| 30 | Title, Abstract, Subject, Author Supplied Keywords | "executive function*" adj5 (child* OR infan* OR student* OR toddler* OR "pre-adolescen*" OR youth OR adolescen* OR preschool* OR kinder*) |
| 31 | Title, Abstract, Subject, Author Supplied Keywords | 19 OR 20 OR 21 OR 22 OR 23 OR 24 OR 25 OR 26 OR 27 OR 28 OR 29 OR 30 |
| 32 | Title, Abstract, Subject, Author Supplied Keywords | 17 AND 18 AND 31 |
| 33 | Title, Abstract, Subject, Author Supplied Keywords | 16 OR 32 |

Table A2. Search Strategy Applied to PsycINFO (Ovid)

| APA PsycInfo <10 April 2025> | |
| --- | --- |
|  |  |
| 1 | ("comparison condition*" or "comparison group*" or "control condition*" or "control group*" or evaluat* or experiment* or interven* or match* or meta-analy* or pilot* or placebo* or program* or "propensity score*" or random* or RCT or review* or therap* or train* or treat* or trial*).ab,hw,id,ti. |
| 2 | (physical or active or activity or exercise* or move* or sport* or action* or bilateral or proprioception or danc* or martial).ab,hw,id,ti. |
| 3 | (cogniti* adj5 (child* or infan* or student* or toddler* or "pre-adolescen*" or youth or adolescen* or preschool* or kinder*)).ab,hw,id,ti. |
| 4 | (inhibit* adj5 (child* or infan* or student* or toddler* or "pre-adolescen*" or youth or adolescen* or preschool* or kinder*)).ab,hw,id,ti. |
| 5 | (memory adj5 (child* or infan* or student* or toddler* or "pre-adolescen*" or youth or adolescen* or preschool* or kinder*)).ab,hw,id,ti. |
| 6 | (attention adj5 (child* or infan* or student* or toddler* or "pre-adolescen*" or youth or adolescen* or preschool* or kinder*)).ab,hw,id,ti. |
| 7 | (spatial adj5 (child* or infan* or student* or toddler* or "pre-adolescen*" or youth or adolescen* or preschool* or kinder*)).ab,hw,id,ti. |
| 8 | (receptive adj5 (child* or infan* or student* or toddler* or "pre-adolescen*" or youth or adolescen* or preschool* or kinder*)).ab,hw,id,ti. |
| 9 | (expressive adj5 (child* or infan* or student* or toddler* or "pre-adolescen*" or youth or adolescen* or preschool* or kinder*)).ab,hw,id,ti. |
| 10 | (reasoning adj5 (child* or infan* or student* or toddler* or "pre-adolescen*" or youth or adolescen* or preschool* or kinder*)).ab,hw,id,ti. |
| 11 | (processing adj5 (child* or infan* or student* or toddler* or "pre-adolescen*" or youth or adolescen* or preschool* or kinder*)).ab,hw,id,ti. |
| 12 | ("problem-solv*" adj5 (child* or infan* or student* or toddler* or "pre-adolescen*" or youth or adolescen* or preschool* or kinder*)).ab,hw,id,ti. |
| 13 | ("problem solv*" adj5 (child* or infan* or student* or toddler* or "pre-adolescen*" or youth or adolescen* or preschool* or kinder*)).ab,hw,id,ti. |
| 14 | ("executive function*" adj5 (child* or infan* or student* or toddler* or "pre-adolescen*" or youth or adolescen* or preschool* or kinder*)).ab,hw,id,ti. |
| 15 | 3 or 4 or 5 or 6 or 7 or 8 or 9 or 10 or 11 or 12 or 13 or 14 |
| 16 | 1 and 2 and 15 |
| 17 | ("comparison condition*" or "comparison group*" or "control condition*" or "control group*" or evaluat* or experiment* or interven* or match* or meta-analy* or pilot* or placebo* or program* or "propensity score*" or random* or RCT or review* or therap* or train* or treat* or trial*).ab,hw,id,ti. |
| 18 | (rhythm* or music* or beat* or synchroni* or entrain*).ab,hw,id,ti. |
| 19 | (cogniti* adj5 (child* or infan* or student* or toddler* or "pre-adolescen*" or youth or adolescen* or preschool* or kinder*)).ab,hw,id,ti. |
| 20 | (inhibit* adj5 (child* or infan* or student* or toddler* or "pre-adolescen*" or youth or adolescen* or preschool* or kinder*)).ab,hw,id,ti. |
| 21 | (memory adj5 (child* or infan* or student* or toddler* or "pre-adolescen*" or youth or adolescen* or preschool* or kinder*)).ab,hw,id,ti. |
| 22 | (attention adj5 (child* or infan* or student* or toddler* or "pre-adolescen*" or youth or adolescen* or preschool* or kinder*)).ab,hw,id,ti. |
| 23 | (spatial adj5 (child* or infan* or student* or toddler* or "pre-adolescen*" or youth or adolescen* or preschool* or kinder*)).ab,hw,id,ti. |
| 24 | (receptive adj5 (child* or infan* or student* or toddler* or "pre-adolescen*" or youth or adolescen* or preschool* or kinder*)).ab,hw,id,ti. |
| 25 | (expressive adj5 (child* or infan* or student* or toddler* or "pre-adolescen*" or youth or adolescen* or preschool* or kinder*)).ab,hw,id,ti. |
| 26 | (reasoning adj5 (child* or infan* or student* or toddler* or "pre-adolescen*" or youth or adolescen* or preschool* or kinder*)).ab,hw,id,ti. |
| 27 | (processing adj5 (child* or infan* or student* or toddler* or "pre-adolescen*" or youth or adolescen* or preschool* or kinder*)).ab,hw,id,ti. |
| 28 | ("problem-solv*" adj5 (child* or infan* or student* or toddler* or "pre-adolescen*" or youth or adolescen* or preschool* or kinder*)).ab,hw,id,ti. |
| 29 | ("problem solv*" adj5 (child* or infan* or student* or toddler* or "pre-adolescen*" or youth or adolescen* or preschool* or kinder*)).ab,hw,id,ti. |
| 30 | ("executive function*" adj5 (child* or infan* or student* or toddler* or "pre-adolescen*" or youth or adolescen* or preschool* or kinder*)).ab,hw,id,ti. |
| 31 | 19 or 20 or 21 or 22 or 23 or 24 or 25 or 26 or 27 or 28 or 29 or 30 |
| 32 | 17 and 18 and 31 |
| 33 | 16 or 32 |
| 34 | limit 33 to (human and english language and english and human) |

Table A3. Databases Searched

| Indexes | Provider |
| --- | --- |
| Campbell Systematic Reviews | Campbell Collaboration |
| Cochrane Database of Systematic Reviews | Cochrane Library |
| Cochrane Central Register of Controlled Trials (CENTRAL) |  |
| Database of Abstracts of Reviews of Effectiveness |  |
| CINAHL | EBSCO |
| Education Source |  |
| Education Resources Information Center (ERIC) |  |
| SPORT Discussion |  |
| Embase | Elsevier |
| MEDLINE | OVID |
| PsycARTICLES |  |
| PsycEXTRA |  |
| PsycINFO |  |
| Dissertation and Theses Global | ProQuest |
| International Bibliography of the Social Sciences |  |
| Psychology Journals |  |
| Research Library |  |
| Social Science Database |  |
| Music Periodicals Database |  |
| Web of Science Core Collection | Web of Science |
| Arts & Humanities Citation Index |  |
| Social Science Citation Index |  |
| Conference Proceedings Citation Index |  |

Table A4. Screening Questions

| Screening Level | Document Screening | Screening Questions | Screening Response Options | | | Additional Information for Screening |
| --- | --- | --- | --- | --- | --- | --- |
| 1 | Abstract | Is the document a duplicate / ineligible document type? | Yes | No |  | Ineligible – not peer-reviewed (i.e. magazine article, news/journalist article, etc.) |
|  |  | IF YES, THEN EXCLUDE | | | | |
|  |  | Does the document have an ineligible population? | Yes | No |  | Ineligible – mean age of participants greater than 12.5 years. |
|  |  | IF YES, THEN EXCLUDE | | | | |
|  |  | Is there discussion/description of some type of relevant intervention? | Yes | No |  | Must mention something about one of the three types of eligible intervention – does not require enough detail to determine eligibility, simply to determine relevance. If unsure, mediate or answer yes and determine at full text screening. |
|  |  | IF NO, THEN EXCLUDE | | | | |
|  |  | Has it been verified to be available only in a language other than English? | Yes | Requires verification |  |  |
|  |  | IF YES, THEN EXCLUDE | | | | |
| 2 | Abstract | Cannot screen on abstract, need to contact authors (order and conference abstracts only) | Yes |  |  |  |
|  |  | IF YES, THEN EXCECUTE CONTACT/ORDER PROTOCOL AND CEASE SCREENING | | | | |
|  |  | Is the document a duplicate / ineligible document type? | Yes | No |  |  |
|  |  | IF YES, THEN EXCLUDE | | | | |
|  |  | Does the document have an ineligible population? | Yes | No |  |  |
|  |  | IF YES, THEN EXCLUDE | | | | |
|  |  | Does the document have an ineligible outcome measure(s)? | Yes | No |  |  |
|  |  | IF YES, THEN EXCLUDE | | | | |
|  |  | Does the document have an ineligible intervention? | Yes | No |  |  |
|  |  | IF YES, THEN EXCLUDE | | | | |
| 3 | Full Text  &  Preliminary Coding: Outcomes | Is there an evaluation of the intervention using eligible outcomes and participants? | Yes | No |  |  |
|  |  | IF NO, THEN EXCLUDE | | | | |
|  |  | Select the research design. | Drop down option |  |  | Drop Down: RCT, Matched group with/without baseline, Unmatched group with/without baseline, Single group pre-post, Case study, Ongoing study, Multi-arm trial (RCT), Multi-arm trial (Quasi), Review/Meta-analysis, Other |
|  |  | IF RESEARCH DESIGN: CASE STUDY (EXCLUDE), ONGOING STUDY (TRIAGE), OR REVIEW (HARVEST) | | | | |
|  |  | Are the outcomes, direct, indirect, or both? | Direct Only | Indirect Only | Both |  |
|  |  | Drop Down: Enter the construct for each direct/indirect measure. |  |  |  | Drop Down: Typed response |
|  |  | Drop Down: Enter the construct’s assessment measure name. |  |  |  | Drop Down: Typed response |
| 4 | Coding: Study Summary | Document Type |  |  |  |  |
|  |  | Country of intervention |  |  |  |  |
|  |  | Was the intervention/evaluation funded? | No | Yes (specify funding source – typed response) |  |  |
|  |  | Is there a trial register or prospective protocol for this evaluation? | Yes (Protocol number – typed response) | No | Unsure |  |
|  | Coding: Participant | Ex: Who are the participants? | Children only | Children and Caregiver Dyads |  |  |
|  |  | See Table A4 for all data coding forms | | | | |
|  | Coding: Intervention |  |  |  |  |  |
|  |  | Ex: What is the intervention category? | Complex coordinated movement | Rhythmic | Both |  |
|  |  | See Table A4 for all data coding forms | | | | |

Table A5. Coding Data Extracted

| **Data Extracted** | **Response Selection Options (if applicable)** | **Additional Instructions** |
| --- | --- | --- |
| **Basic Study Information** | | |
| Reference (APA) – Study authors, year of publication, study title, journal/publisher, volume number, issue number, page number, URL/DOI/source. | APA 7 formatted export | Endnote citation export. |
| Country of intervention | List of all available current countries and an ‘other’ fillable category provided. |  |
| Publication type | Journal article, protocol or trial register, thesis, book chapter, report, conference paper. |  |
| Funding source (if applicable) | Typed response. |  |
| Number of dependent documents | Number response and Reference ID number. |  |
| Study Design | RCT, RCT (Crossover), Quasi (Randomized controlled), Quasi (Matched), Quasi (Unmatched), Quasi (Crossover), Quasi (Single Group Pre-Post). |  |
| Study Design - Comparator | Control, Waitlist Control, Active Control, Alternative Treatment, Treatment as Usual, Not Applicable (Single Group Pre-Post) |  |
| **Participant Information*** | | |
| Participant type | Just Children or Children and Caregiver dyads |  |
| N participants recruited - comparison | Number response |  |
| N participants recruited - intervention | Number response |  |
| N participants began - comparison | Number response |  |
| N participants began - intervention | Number response |  |
| N participants completed - comparison | Number response |  |
| N participants completed - intervention | Number response |  |
| Mean age of participants – comparison group | Number response | If mean not available, age range accepted |
| Mean age of participants – intervention group | Number response | If mean not available, age range accepted |
| Mean age of participants – total | Number response | If mean not available, age range accepted |
| % of female participants – comparison group | Percentage response | If percentage unable to be calculated, N accepted |
| % of female participants – intervention group | Percentage response |  |
| % of female participants – total | Percentage response |  |
| Clinical population | Typed response | Only include if majority of population or study focus for intervention (i.e. Typical vs Clinical groups); Record name of clinical group, N or percentage of group compared to comparison, intervention, and total Ns. |
| Ethnicity | Typed response | Percentage or N of each ethnicity reported for comparison group, intervention group, and total. |
| Additional sampling information | Typed response | Any relevant information such as participant medication, socioeconomic measures, etc. |
| **Outcome Information** | | |
| Outcome construct category | Executive Function, Memory, Cognition (multiple responses permitted). |  |
| Outcome Assessment Measure | Typed response – Assessment title | Each construct has a drop down to label each assessment(s) to the construct. |
| Outcome method | Direct, Indirect, Both | For overall outcome measures across constructs. |
| **Intervention Information*** | | |
| Intervention category | Complex coordinated movement, Rhythmic, Both |  |
| Intervention Name | Typed response – Title of intervention program or (if not available) summary title of intervention program. | Summary title should include the key intervention elements. |
| Intervention Subcategory | Typed response (multiple responses permitted). | Responses provided from separate table of common subcategories i.e. Active breaks, exergaming, sport, group games, group music, instrumental, martial arts, gymnastics, etc. |
| Intervention Setting | Educational (options: Preschool, Kindergarten, Primary School, Elementary School, After-school care/program, Before-school care/program, Summer school camp/care/program); Health clinic; Other (typed response – typical responses included Home, Community Centre, Hospital; Sport-related Centre, Music-related Centre) |  |
| Intervention Implementers | Teachers, Parents/Caregivers, Physical Therapists, Occupational Therapists, Social Workers, Other qualified professions (typed response), Other allied health practitioners (typed response), Other (typed response). | Qualified professional was defined as qualified in the domain of the intervention, either through training or expertise. |
| Intervention Delivery Method | Group, Individual, Other (typed response) |  |
| Intervention Delivery Modality | Face-to-face, Audiovisual mode, Telehealth, Other (typed response) |  |
| Intervention Session - Number | Number response | When exact intervention session number was not calculated, duration of intervention (e.g. 2 years) was provided. |
| Intervention Session - Frequency | Daily, Weekly, Fortnightly, Monthly, Yearly |  |
| Intervention Session – Frequency N | Number response | Number of times per frequency (i.e. weekly, fortnightly) the intervention occurred. |
| Intervention Session – Length of Session | <5 minutes, 5-14 minutes, 15-29 minutes, 30-59 minutes, 60 minutes, 61-90 minutes, >90 minutes. | If intervention session length varied each session, the average session length was selected, and a note was made under session length other notes. |

*If multiple eligible interventions, entered in additional coding forms for participant and intervention information and labelled Intervention 1, Intervention 2, etc. consistently throughout.

A6. PRISMA Checklist

| **Section and Topic** | **Item #** | **Checklist item** | **Location where item is reported** |
| --- | --- | --- | --- |
| **TITLE** | | |  |
| Title | 1 | Identify the report as a systematic review. | Page 1 |
| **ABSTRACT** | | |  |
| Abstract | 2 | See the PRISMA 2020 for Abstracts checklist. | Page 2 |
| **INTRODUCTION** | | |  |
| Rationale | 3 | Describe the rationale for the review in the context of existing knowledge. | Pages 4-9 |
| Objectives | 4 | Provide an explicit statement of the objective(s) or question(s) the review addresses. | Pages 9-11 |
| **METHODS** | | |  |
| Eligibility criteria | 5 | Specify the inclusion and exclusion criteria for the review and how studies were grouped for the syntheses. | Pages 12-16 |
| Information sources | 6 | Specify all databases, registers, websites, organisations, reference lists and other sources searched or consulted to identify studies. Specify the date when each source was last searched or consulted. | Pages 12  Appendix A2 |
| Search strategy | 7 | Present the full search strategies for all databases, registers and websites, including any filters and limits used. | Appendix A1 |
| Selection process | 8 | Specify the methods used to decide whether a study met the inclusion criteria of the review, including how many reviewers screened each record and each report retrieved, whether they worked independently, and if applicable, details of automation tools used in the process. | Pages 13-15 |
| Data collection process | 9 | Specify the methods used to collect data from reports, including how many reviewers collected data from each report, whether they worked independently, any processes for obtaining or confirming data from study investigators, and if applicable, details of automation tools used in the process. | Pages 16-18; Appendix A3, A4 |
| Data items | 10a | List and define all outcomes for which data were sought. Specify whether all results that were compatible with each outcome domain in each study were sought (e.g. for all measures, time points, analyses), and if not, the methods used to decide which results to collect. | Page 13-14 |
|  | 10b | List and define all other variables for which data were sought (e.g. participant and intervention characteristics, funding sources). Describe any assumptions made about any missing or unclear information. | Pages 12-16 |
| Study risk of bias assessment | 11 | Specify the methods used to assess risk of bias in the included studies, including details of the tool(s) used, how many reviewers assessed each study and whether they worked independently, and if applicable, details of automation tools used in the process. | Not relevant for EGM review. |
| Effect measures | 12 | Specify for each outcome the effect measure(s) (e.g. risk ratio, mean difference) used in the synthesis or presentation of results. | N/A for EGMs |
| Synthesis methods | 13a | Describe the processes used to decide which studies were eligible for each synthesis (e.g. tabulating the study intervention characteristics and comparing against the planned groups for each synthesis (item #5)). | Appendix A3, A4 |
|  | 13b | Describe any methods required to prepare the data for presentation or synthesis, such as handling of missing summary statistics, or data conversions. | N/A for EGMs |
|  | 13c | Describe any methods used to tabulate or visually display results of individual studies and syntheses. | N/A for EGMs |
|  | 13d | Describe any methods used to synthesize results and provide a rationale for the choice(s). If meta-analysis was performed, describe the model(s), method(s) to identify the presence and extent of statistical heterogeneity, and software package(s) used. | N/A for EGMs |
|  | 13e | Describe any methods used to explore possible causes of heterogeneity among study results (e.g. subgroup analysis, meta-regression). | N/A for EGMs |
|  | 13f | Describe any sensitivity analyses conducted to assess robustness of the synthesized results. | N/A for EGMs |
| Reporting bias assessment | 14 | Describe any methods used to assess risk of bias due to missing results in a synthesis (arising from reporting biases). | N/A for EGMs |
| Certainty assessment | 15 | Describe any methods used to assess certainty (or confidence) in the body of evidence for an outcome. | N/A for EGMs |
| **RESULTS** | | |  |
| Study selection | 16a | Describe the results of the search and selection process, from the number of records identified in the search to the number of studies included in the review, ideally using a flow diagram. | Pages 18-20 |
|  | 16b | Cite studies that might appear to meet the inclusion criteria, but which were excluded, and explain why they were excluded. | Not conducted |
| Study characteristics | 17 | Cite each included study and present its characteristics. | Appendix C |
| Risk of bias in studies | 18 | Present assessments of risk of bias for each included study. | N/A for EGMs |
| Results of individual studies | 19 | For all outcomes, present, for each study: (a) summary statistics for each group (where appropriate) and (b) an effect estimate and its precision (e.g. confidence/credible interval), ideally using structured tables or plots. | N/A for EGMs |
| Results of syntheses | 20a | For each synthesis, briefly summarise the characteristics and risk of bias among contributing studies. | N/A for EGMs |
|  | 20b | Present results of all statistical syntheses conducted. If meta-analysis was done, present for each the summary estimate and its precision (e.g. confidence/credible interval) and measures of statistical heterogeneity. If comparing groups, describe the direction of the effect. | Pages 18--25 |
|  | 20c | Present results of all investigations of possible causes of heterogeneity among study results. | N/A for EGMs |
|  | 20d | Present results of all sensitivity analyses conducted to assess the robustness of the synthesized results. | N/A for EGMs |
| Reporting biases | 21 | Present assessments of risk of bias due to missing results (arising from reporting biases) for each synthesis assessed. | N/A for EGMs |
| Certainty of evidence | 22 | Present assessments of certainty (or confidence) in the body of evidence for each outcome assessed. | N/A for EGMs |
| **DISCUSSION** | | |  |
| Discussion | 23a | Provide a general interpretation of the results in the context of other evidence. | Pages 25-28 |
|  | 23b | Discuss any limitations of the evidence included in the review. | Pages 28-30 |
|  | 23c | Discuss any limitations of the review processes used. | Pages 30 |
|  | 23d | Discuss implications of the results for practice, policy, and future research. | Pages 28-30 |
| **OTHER INFORMATION** | | |  |
| Registration and protocol | 24a | Provide registration information for the review, including register name and registration number, or state that the review was not registered. | Page 2, 12 |
|  | 24b | Indicate where the review protocol can be accessed, or state that a protocol was not prepared. | Page 2, 12 |
|  | 24c | Describe and explain any amendments to information provided at registration or in the protocol. | Page 12 |
| Support | 25 | Describe sources of financial or non-financial support for the review, and the role of the funders or sponsors in the review. | Page 31 |
| Competing interests | 26 | Declare any competing interests of review authors. | Page 31 |
| Availability of data, code and other materials | 27 | Report which of the following are publicly available and where they can be found: template data collection forms; data extracted from included studies; data used for all analyses; analytic code; any other materials used in the review. | Page 31 |

*From:*  Page MJ, McKenzie JE, Bossuyt PM, Boutron I, Hoffmann TC, Mulrow CD, et al. The PRISMA 2020 statement: an updated guideline for reporting systematic reviews. BMJ 2021;372:n71. doi: 10.1136/bmj.n7
