# Supplementary material for: A Systematic Review and Evidence Gap Map Evaluation of Rhythmic and/or Complex Movement Interventions and Child Cognitive Outcomes
Source: Clin Child Fam Psychol Rev. 2025 Oct 11;28(4):912–29. doi: 10.1007/s10567-025-00547-1 (PMC12660398; doi:10.1007/s10567-025-00547-1)
Supplement: Supplementary file 2 — Supplementary file2 (DOCX 395 KB) [file 10567_2025_547_MOESM2_ESM.docx]

**Supplementary Materials B – Figures**

B1. Figure EGM 3 – Intervention Methodology

<https://figshare.com/ndownloader/files/56231741>


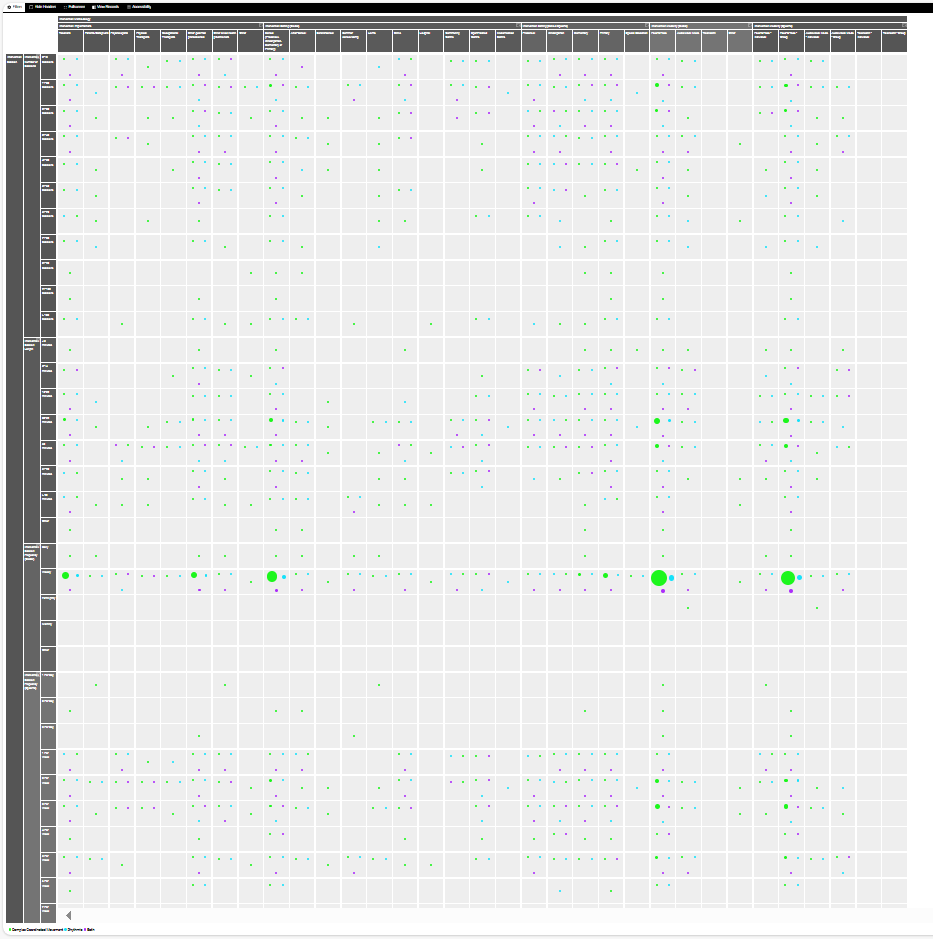


B2. Figures Included in Manuscript

**Figure 1**

*PRISMA Flow Diagram*

Records identified in systematic search up to April 2025

*n* = 108,276

Duplicates and LOTE removed *n* = 46,716

Records screened on title and abstract

*n* = 61,560

(16,308 human; 45,252 machine)

Duplicate or ineligible document type *n* = 206

Ineligible population *n* = 3,444

Ineligible intervention *n* = 9,979

Machine excluded *n* = 45,252

fnn

Harvested review *n* = 2

On order or awaiting classification *n* = 52

Ongoing or inactive trials *n* = 59

Duplicate or ineligible document type *n* = 154

Ineligible population *n* = 145

Ineligible intervention *n* = 765

Ineligible outcome *n* = 943

No impact evaluation using eligible research design (*n* = 86)

jhm

Records eligible for full-text eligibility screening

*n* = 2,679

Studies included in review and EGMs

*n* = 402 (473 reports)

**Figure 2**

*Study Country Frequencies*


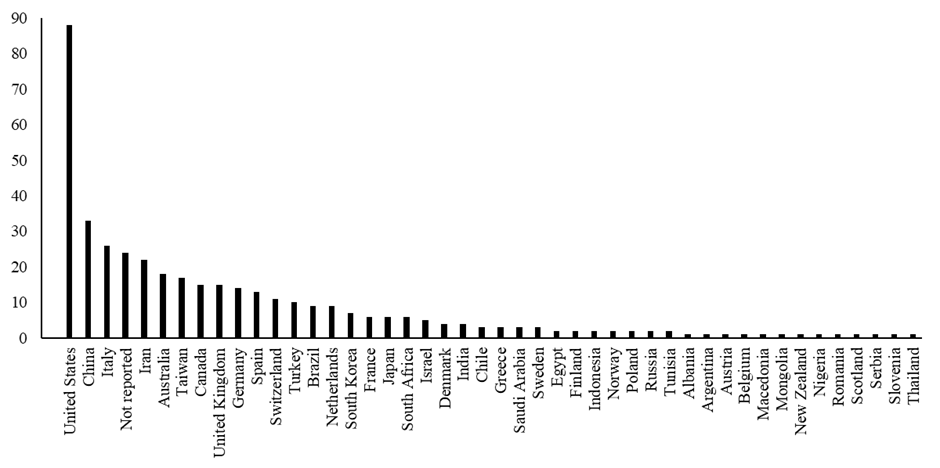


**Figure 3**

*Evidence and Gap Map 1*


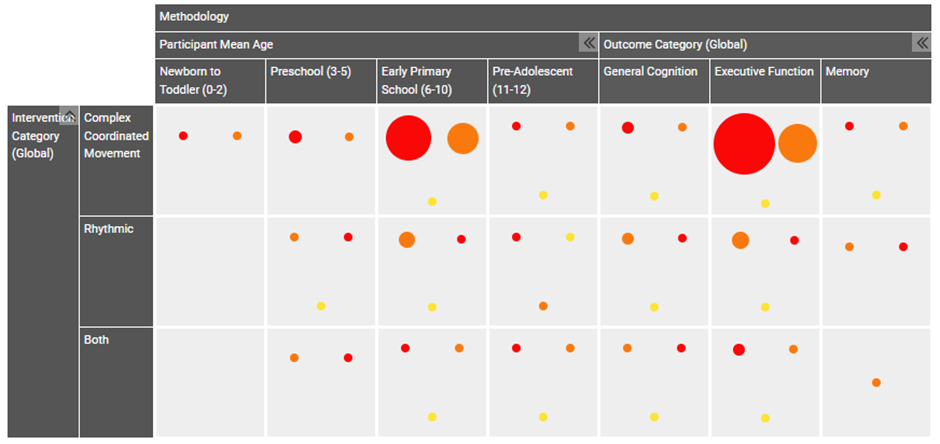


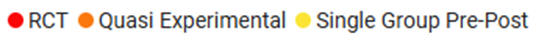


Interactive EGM 1 Hyperlink:

<https://figshare.com/ndownloader/files/56231684>

**Figure 4**

*Evidence and Gap Map 2*


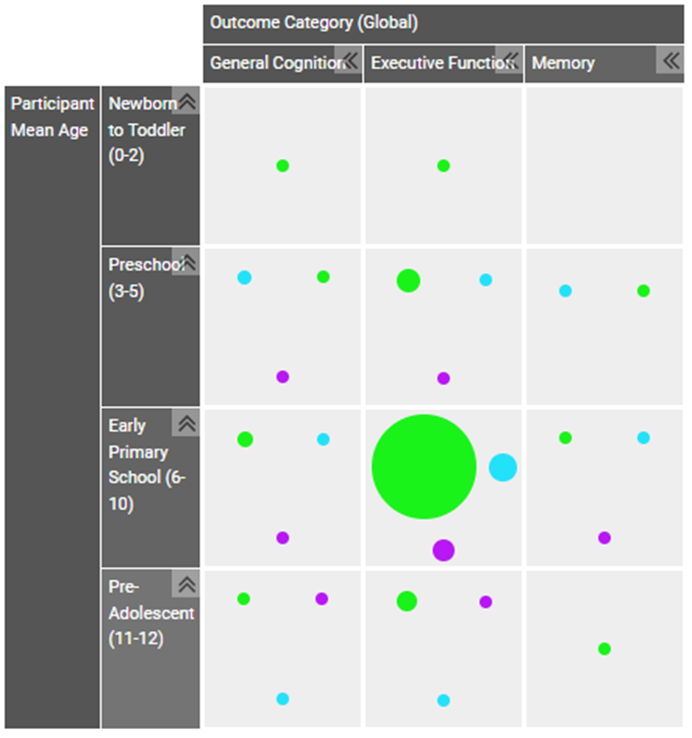


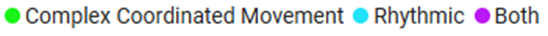


Interactive EGM 2 Hyperlink:

<https://figshare.com/ndownloader/files/56231720>
