# Supplementary material for: A Systematic Review and Evidence Gap Map Evaluation of Rhythmic and/or Complex Movement Interventions and Child Cognitive Outcomes
Source: Clin Child Fam Psychol Rev. 2025 Oct 11;28(4):912–29. doi: 10.1007/s10567-025-00547-1 (PMC12660398; doi:10.1007/s10567-025-00547-1)
Supplement: Supplementary file 3 — Supplementary file3 (DOCX 131 KB) [file 10567_2025_547_MOESM3_ESM.docx]

**Supplementary Materials C – Included Studies**

**C.1 Reference list of included parent studies in EGM – Original Search (Sept 2021)**

Aadland, K. N., Ommundsen, Y., Anderssen, S. A., Bronnick, K. S., Moe, V. F., Resaland, G. K., Skrede, T., Stavnsbo, M., & Aadland, E. (2019). Effects of the active smarter kids (ask) physical activity school-based intervention on executive functions: A Cluster-randomized controlled trial. *Scandinavian Journal of Educational Research, 63*(2), 214-228. https://doi.org/10.1080/00313831.2017.1336477

Adsiz, E., Dorak, F., Ozsaker, M., & Vurgun, N. (2012). The influence of physical activity on attention in Turkish children. *HealthMED, 6*(4), 1384-1389. https://www.embase.com/search/results?subaction=viewrecord&id=L364870508&from=export http://www.drunpp.ba/pdf/healthmed_6_4_web.pdf

Alesi, M., Bianco, A., Luppina, G., Palma, A., & Pepi, A. (2016). Improving children's coordinative skills and executive functions: The effects of a football exercise program. *Perceptual and Motor Skills, 122*(1), 27-46. https://doi.org/10.1177/0031512515627527

Alesi, M., Bianco, A., Padulo, J., Luppina, G., Petrucci, M., Paoli, A., Palma, A., & Pepi, A. (2015). Motor and cognitive growth following a football training program. *Frontiers in Psychology*, 6. https://ovidsp.ovid.com/ovidweb.cgi?T=JS&PAGE=reference&D=psyc12&NEWS=N&AN=2016-22890-001

Alloway, T. P., & Warner, C. (2008). Task-specific training, learning, and memory for children with developmental coordination disorder: A pilot study. *Perceptual & Motor Skills, 107*(2), 473-480. https://doi.org/10.2466/PMS.107.6.473-480

Almeida, A. (2002). The impact of increased music notation instruction on the perceptual speed of third-grade students in a public school setting (Publication Number 3042941) [Ed.D., University of Central Florida]. ProQuest Dissertations & Theses Global. Ann Arbor. http://search.proquest.com.libraryproxy.griffith.edu.au/dissertations-theses/impact-increased-music-notation-instruction-on/docview/305438842/se-2?accountid=14543 http://hy8fy9jj4b.search.serialssolutions.com/directLink?&atitle=The+impact+of+increased+music+notation+instruction+on+the+perceptual+speed+of+third-grade+students+in+a+public+school+setting&author=Almeida%2C+Artie&issn=&title=The+impact+of+increased+music+notation+instruction+on+the+perceptual+speed+of+third-grade+students+in+a+public+school+setting&volume=&issue=&date=2002-01-01&spage=&id=doi:&sid=ProQ_ss&genre=article

Alrazain, B. (2016). Developing and evaluating an arts therapies programme for children with attention deficit hyperactivity disorder (ADHD) in primary schools in the kingdom of sAudi Arabia (Ksa) (Publication Number 10303546) [Ph.D., Queen Margaret University (United Kingdom)]. ProQuest Dissertations & Theses Global. Ann Arbor. http://search.proquest.com.libraryproxy.griffith.edu.au/dissertations-theses/developing-evaluating-arts-therapies-programme/docview/1857845086/se-2?accountid=14543 http://hy8fy9jj4b.search.serialssolutions.com/directLink?&atitle=Developing+and+evaluating+an+arts+therapies+programme+for+children+with+attention+deficit+hyperactivity+disorder+%28adhd%29+in+primary+schools+in+the+kingdom+of+saudi+arabia+%28ksa%29&author=Alrazain%2C+Badr&issn=&title=Developing+and+evaluating+an+arts+therapies+programme+for+children+with+attention+deficit+hyperactivity+disorder+%28adhd%29+in+primary+schools+in+the+kingdom+of+saudi+arabia+%28ksa%29&volume=&issue=&date=2016-01-01&spage=&id=doi:&sid=ProQ_ss&genre=article

Alrazain, B., Zubala, A., & Karkou, V. (2018). Movement-based arts therapy for children with attention deficit hyperactivity disorder (ADHD) in the Kingdom of Saudi Arabia. https://doi.org/10.4324/9781315454412

Altenburg, T. M., Chinapaw, M. J. M., & Singh, A. S. (2016). Effects of one versus two bouts of moderate intensity physical activity on selective attention during a school morning in Dutch primary schoolchildren: A randomized controlled trial. Journal of Science & Medicine in Sport, 19(10), 820-824. https://doi.org/10.1016/j.jsams.2015.12.003

Arias Rodriguez, I. N., Jessica M. Voigt, Marcos F. Santos, Flavia H. (2019). Numeracy musical training for school children with low achievement in mathematics [Academic Learning & Achievement 3550]. Anales de Psicologia, 35(3), 405-416. https://doi.org/http://dx.doi.org/10.6018/analesps.35.3.340091

Austin, S. (2014). Movin' and groovin' creative movement group for children diagnosed with ADHD (Publication Number 3739810) [Psy.D., The Chicago School of Professional Psychology]. ProQuest Dissertations & Theses Global. Ann Arbor. http://search.proquest.com.libraryproxy.griffith.edu.au/dissertations-theses/movin-groovin-creative-movement-group-children/docview/1749792477/se-2?accountid=14543 http://hy8fy9jj4b.search.serialssolutions.com/directLink?&atitle=Movin%27+and+Groovin%27+Creative+Movement+Group+for+Children+Diagnosed+With+ADHD&author=Austin%2C+Stacey&issn=&title=Movin%27+and+Groovin%27+Creative+Movement+Group+for+Children+Diagnosed+With+ADHD&volume=&issue=&date=2014-01-01&spage=&id=doi:&sid=ProQ_ss&genre=article

Baglio, G., Zanette, M., Di Cesare, M., Di Tella, S., Clerici, M., Baglio, F., & Blasi, V. (2021). Rehabilitation and disability spectrum from adverse childhood experience: the impact of the movement cognition and narration of emotions treatment (MCNT) version 2.0. Frontiers in psychiatry, 11, 609819.

Baker, T. C. (2005). The use of mini -exercise breaks in the classroom management of ADHD -type behaviors (Publication Number 3179064) [Ph.D., Capella University]. ProQuest Dissertations & Theses Global. Ann Arbor. http://search.proquest.com.libraryproxy.griffith.edu.au/dissertations-theses/use-mini-exercise-breaks-classroom-management/docview/305358905/se-2?accountid=14543 http://hy8fy9jj4b.search.serialssolutions.com/directLink?&atitle=The+use+of+mini+-exercise+breaks+in+the+classroom+management+of+ADHD+-type+behaviors&author=Baker%2C+Teresa+C.&issn=&title=The+use+of+mini+-exercise+breaks+in+the+classroom+management+of+ADHD+-type+behaviors&volume=&issue=&date=2005-01-01&spage=&id=doi:&sid=ProQ_ss&genre=article

Barbaroux, M., Dittinger, E., & Besson, M. (2019). Music training with Demos program positively influences cognitive functions in children from low socio-economic backgrounds. *PLoS ONE, 14*(5), e0216874. https://doi.org/10.1371/journal.pone.0216874

Battaglia, G., Alesi, M., Tabacchi, G., Palma, A., & Bellafiore, M. (2019). The development of motor and pre-literacy skills by a physical education program in preschool children: A non-randomized pilot trial. *Frontiers In Psychology*, *9*. https://doi.org/http://dx.doi.org/10.3389/fpsyg.2018.02694

Bear, J. R. (2005). The relationship between teaching kindergarten students' reading, writing, and performance of rhythmic notation and increased spatial skills (Publication Number 3196190) [Ph.D., Wayne State University]. ProQuest Dissertations & Theses Global. Ann Arbor. http://search.proquest.com.libraryproxy.griffith.edu.au/dissertations-theses/relationship-between-teaching-kindergarten/docview/305386895/se-2?accountid=14543 http://hy8fy9jj4b.search.serialssolutions.com/directLink?&atitle=The+relationship+between+teaching+kindergarten+students%27+reading%2C+writing%2C+and+performance+of+rhythmic+notation+and+increased+spatial+skills&author=Bear%2C+Jessica+Rose&issn=&title=The+relationship+between+teaching+kindergarten+students%27+reading%2C+writing%2C+and+performance+of+rhythmic+notation+and+increased+spatial+skills&volume=&issue=&date=2005-01-01&spage=&id=doi:&sid=ProQ_ss&genre=article

Beck, M. M., Lind, R. R., Geertsen, S. S., Ritz, C., Lundbye-Jensen, J., & Wienecke, J. (2016). Motor-enriched learning activities can improve mathematical performance in preadolescent children. *Frontiers in Human Neuroscience, 10*(DEC2016). https://doi.org/10.3389/fnhum.2016.00645

Bégel, V., Bachrach, A., Dalla Bella, S., Laroche, J., Clément, S., Riquet, A., & Dellacherie, D. (2021). Dance improves motor, cognitive, and social skills in children with developmental cerebellar anomalies. *Cerebellum.* https://doi.org/10.1007/s12311-021-01291-2

Benítez, M. A., Abrahan, V. D., & Justel, N. (2020). Active and receptive musical training affects emotional and neutral memory in 4-and 5-year-old children. *ASN Neuro, 13,* 75. https://doi.org/10.1177/1759091420979851

Benzing, V., & Schmidt, M. (2019). The effect of exergaming on executive functions in children with ADHD: A randomized clinical trial. *Scandinavian Journal of Medicine & Science in Sports, 29*(8), 1243‐1253. https://doi.org/10.1111/sms.13446

Benzing, V., Spitzhüttl, J., Siegwart, V., Schmid, J., Grotzer, M., Heinks, T., Roebers, C. M., Steinlin, M., Leibundgut, K., & Schmidt, M. (2020). Effects of cognitive training and exergaming in pediatric cancer survivors- A randomized clinical trial. *Medicine and Science in Sports and Exercise, 52*(11), 2293‐2302. https://doi.org/10.1249/MSS.0000000000002386

Berent, R. C. (2017). The impact of music therapy on sensory gating and attention abilities in children with autism spectrum disorder: A feasibility study (Publication Number 10604727) [M.S., Colorado State University]. ProQuest Dissertations & Theses Global. Ann Arbor. http://search.proquest.com.libraryproxy.griffith.edu.au/dissertations-theses/impact-music-therapy-on-sensory-gating-attention/docview/1978469958/se-2?accountid=14543 http://hy8fy9jj4b.search.serialssolutions.com/directLink?&atitle=The+Impact+of+Music+Therapy+on+Sensory+Gating+And+Attention+Abilities+in+Children+with+Autism+Spectrum+Disorder%3A+A+Feasibility+Study&author=Berent%2C+Rachel+C.&issn=&title=The+Impact+of+Music+Therapy+on+Sensory+Gating+And+Attention+Abilities+in+Children+with+Autism+Spectrum+Disorder%3A+A+Feasibility+Study&volume=&issue=&date=2017-01-01&spage=&id=doi:&sid=ProQ_ss&genre=article

Bertoni, S., Franceschini, S., Puccio, G., Mancarella, M., Gori, S., & Facoetti, A. (2021). Action video games enhance attentional control and phonological decoding in children with developmental dyslexia. Brain Sciences, 11(2), 171.

Besson, M., Schon, D., Moreno, S., Santos, A., & Magne, C. (2007). Influence of musical expertise and musical training on pitch processing in music and language. *Restorative Neurology And Neuroscience, 25*(3-4), 399-410. https://ovidsp.ovid.com/ovidweb.cgi?T=JS&PAGE=reference&D=med6&NEWS=N&AN=17943015

Bilhartz, T. D. B., Rick A. Olson, Judith E. (1999). The effect of early music training on child cognitive development. *Journal of Applied Developmental Psychology, 20*(4), 615-636. https://doi.org/http://dx.doi.org/10.1016/S0193-3973%2899%2900033-7

Blasi, V. Z., M. Baglio, G. Giangiacomo, A. Di Tella, S. Canevini, M. P. Walder, M. Clerici, M. Baglio, F. Grp, B. I. F. (2020). Intervening on the developmental course of children with borderline intellectual functioning with a multimodal intervention: Results from a randomized controlled trial. *Frontiers In Psychology, 11,* Article 679. https://doi.org/10.3389/fpsyg.2020.00679

Bogdanowicz, E. (2016). Dalcroze eurhythmics in therapy for children with attention deficit hyperactivity disorder (ADHD) symptoms: An interdisciplinary journal of music therapy [Η Ρυθμική Dalcroze στη θεραπεία παιδιών με συμπτώματα Διαταραχής Ελλειμματικής Προσοχής και Υπερκινητικότητας (ΔΕΠΥ)]. Approaches, 8(2), 134. http://search.proquest.com.libraryproxy.griffith.edu.au/scholarly-journals/dalcroze-eurhythmics-therapy-children-with/docview/2108872847/se-2?accountid=14543 http://hy8fy9jj4b.search.serialssolutions.com/directLink?&atitle=Dalcroze+Eurhythmics+in+therapy+for+children+with+Attention+Deficit+Hyperactivity+Disorder+%28ADHD%29+symptoms&author=Bogdanowicz%2C+Ewa&issn=&title=Approaches&volume=8&issue=2&date=2016-01-01&spage=134&id=doi:&sid=ProQ_ss&genre=article

Bolduc, J., Gosselin, N., Chevrette, T., & Peretz, I. (2021). The impact of music training on inhibition control, phonological processing, and motor skills in kindergarteners: A randomized control trial. *Early Child Development And Care.* https://doi.org/10.1080/03004430.2020.1781841

Bouallegue, M., Yahya, H. B., & Bouden, A. (2019). Rehabilitation of ADHD children by sport intervention: A Tunisian experience. *Tunisie Medicale, 97*(7), 874-881.

Bowmer, A., Mason, K., Knight, J., & Welch, G. (2018). Investigating the impact of a musical intervention on preschool children's executive function. *Frontiers In Psychology, 9.* https://doi.org/http://dx.doi.org/10.3389/fpsyg.2018.02389

Bridges, C. E. (2018). Improving fitness, executive function, and competence of children with developmental disabilities through an adapted gymnastics intervention https://login.libraryproxy.griffith.edu.au/login?url=https://www.proquest.com/docview/2779135973?accountid=14543&bdid=37598&_bd=S0MMmbddi%2BxQhznZU8SbSi6D2Vw%3D

Brodsky, W., & Sulkin, I. (2011). Handclapping songs: A spontaneous platform for child development among 5-10-year-old children. *Early Child Development & Care, 181*(8), 1111-1136. https://doi.org/10.1080/03004430.2010.517837

Budde, H., & Akko, D. P. (2020). The effects of an exercise training on steroid hormones in school children - A predictor for enhanced cognition? *Journal of Sport & Exercise Psychology, 42,* S69-S69. <Go to ISI>://WOS:000557622200234

Bugos, J. A. D., Darlene. (2017). The effects of a short-term music program on preschool children's executive functions [Curriculum & Programs & Teaching Methods 3530]. PSYCHOLOGY OF MUSIC, 45(6), 855-867. https://doi.org/http://dx.doi.org/10.1177/0305735617692666

BÜKer, N., Kaya², D. Ö., & TÜKel, Ş. (2020). Neurocognitive exercise program improves selective attention in children aged between 7-13 years: a pilot study. / Nörobilişsel egzersiz programı 7-13 yaş arası çocuklarda seçici dikkati geliştirir: pilot çalışma. Journal of Exercise Therapy & Rehabilitation, 7(3), 239-246. https://search.ebscohost.com/login.aspx?direct=true&db=sph&AN=149658775&site=ehost-live&scope=site

Bustamante, E. E. (2013). Physical activity intervention for ADHD and DBD (Publication Number 3604214) [Ph.D., University of Illinois at Chicago]. ProQuest Dissertations & Theses Global. Ann Arbor. http://search.proquest.com.libraryproxy.griffith.edu.au/dissertations-theses/physical-activity-intervention-adhd-dbd/docview/1468954505/se-2?accountid=14543 http://hy8fy9jj4b.search.serialssolutions.com/directLink?&atitle=Physical+activity+intervention+for+ADHD+and+DBD&author=Bustamante%2C+Eduardo+Esteban&issn=&title=Physical+activity+intervention+for+ADHD+and+DBD&volume=&issue=&date=2013-01-01&spage=&id=doi:&sid=ProQ_ss&genre=article

Cancilla, C. E. (2016). Mindfulness martial arts training versus traditional martial arts training as support for improved academic performance in children diagnosed as attention deficit hyperactivity disorder (Publication Number 10170165) [Ph.D., Fielding Graduate University]. ProQuest Dissertations & Theses Global. Ann Arbor. http://search.proquest.com.libraryproxy.griffith.edu.au/dissertations-theses/mindfulness-martial-arts-training-versus/docview/1848299239/se-2?accountid=14543 http://hy8fy9jj4b.search.serialssolutions.com/directLink?&atitle=Mindfulness+martial+arts+training+versus+traditional+martial+arts+training+as+support+for+improved+academic+performance+in+children+diagnosed+as+attention+deficit+hyperactivity+disorder&author=Cancilla%2C+Charles+Edward&issn=&title=Mindfulness+martial+arts+training+versus+traditional+martial+arts+training+as+support+for+improved+academic+performance+in+children+diagnosed+as+attention+deficit+hyperactivity+disorder&volume=&issue=&date=2016-01-01&spage=&id=doi:&sid=ProQ_ss&genre=article

Castello-Juan, B., Anton-Suay, M. T., Flores-Morales, N., Vicedo-Reche, M., & Romero-Naranjo, F. J. (2018, Jun 20-22

2018). EVALUATING EXECUTIVE FUNCTIONS IN PRIMARY SCHOOL CHILDREN IN ALICANTE USING BODY PERCUSSION.European Proceedings of Social and Behavioural Sciences [Viii international conference on intercultural education and international conference on transcultural health: The value of education and health for a global, transcultural world (eduhem 2018)]. Almeria, SPAIN.

Cataldi, S., BonavolontÀ, V., & Fischetti, F. (2021). Starting a sport as outdoor education in infancy: orienteering, visual spatial memory for empowering school learning. Journal of Physical Education & Sport, 21, 696-701. https://search.ebscohost.com/login.aspx?direct=true&db=sph&AN=152036484&site=ehost-live&scope=site

Chacona, S. M. (2007). Effect of world music drumming on auditory and visual attention skills of ADHD elementary students (Publication Number 3274167) [Ph.D., Lynn University]. ProQuest Dissertations & Theses Global. Ann Arbor. http://search.proquest.com.libraryproxy.griffith.edu.au/dissertations-theses/effect-world-music-drumming-on-auditory-visual/docview/304700857/se-2?accountid=14543 http://hy8fy9jj4b.search.serialssolutions.com/directLink?&atitle=Effect+of+world+music+drumming+on+auditory+and+visual+attention+skills+of+ADHD+elementary+students&author=Chacona%2C+Shannon+Michael&issn=&title=Effect+of+world+music+drumming+on+auditory+and+visual+attention+skills+of+ADHD+elementary+students&volume=&issue=&date=2007-01-01&spage=&id=doi:&sid=ProQ_ss&genre=article

Chaddock-Heyman, L., Erickson, K. I., Voss, M. W., Knecht, A. M., Pontifex, M. B., Castelli, D. M., Hillman, C. H., & Kramer, A. F. (2013). The effects of physical activity on functional MRI activation associated with cognitive control in children: a randomized controlled intervention [Journal: Article]. Frontiers in Human Neuroscience, (FEB). https://doi.org/10.3389/fnhum.2013.00072

Chaddock-Heyman, L., Weng, T. B., Kienzler, C., Weisshappel, R., Drollette, E. S., Raine, L. B., Westfall, D. R., Kao, S. C., Baniqued, P., Castelli, D. M., Hillman, C. H., & Kramer, A. F. (2020). Brain Network Modularity Predicts Improvements in Cognitive and Scholastic Performance in Children Involved in a Physical Activity Intervention [Article]. Frontiers in Human Neuroscience, 14. https://doi.org/10.3389/fnhum.2020.00346

Chan, A. S., Han, Y. M. Y., Sze, S. L., & Lau, E. M. (2015). Neuroenhancement of memory for children with autism by a mind-body exercise [Specialized Interventions 3350]. FRONTIERS IN PSYCHOLOGY, 6. https://ovidsp.ovid.com/ovidweb.cgi?T=JS&PAGE=reference&D=psyc12&NEWS=N&AN=2016-24745-001

Chan, A. S., Sze, S. L., Siu, N. Y., Lau, E. M., & Cheung, M. C. (2013). A chinese mind-body exercise improves self-control of children with autism: a randomized controlled trial [Journal Article; Randomized Controlled Trial; Research Support, Non‐U.S. Gov't]. PLoS ONE, 8(7), e68184. https://doi.org/10.1371/journal.pone.0068184

Chan, Y. S., & Ho, C. S. (2021). Reaction Performance Improvement in Children with ADHD through Adapted Physical Activity - A Pilot Study. / Erhöhung der Reaktionsfähigkeit bei Kindern mit ADHS durch angepasste körperliche Aktivität - Eine Pilotstudie. German Journal of Sports Medicine / Deutsche Zeitschrift fur Sportmedizin, 72(1), 21-26. https://search.ebscohost.com/login.aspx?direct=true&db=sph&AN=149037601&site=ehost-live&scope=site

Chang, Y. K., Hung, C. L., Huang, C. J., Hatfield, B. D., & Hung, T. M. (2014). Effects of an aquatic exercise program on inhibitory control in children with ADHD: a preliminary study [Journal: Article]. ARCHIVES OF CLINICAL NEUROPSYCHOLOGY, 29(3), 217‐223. https://doi.org/10.1093/arclin/acu003

Chang, Y. K., Tsai, Y. J., Chen, T. T., & Hung, T. M. (2013). The impacts of coordinative exercise on executive function in kindergarten children: an ERP study. Experimental Brain Research, 225(2), 187-196. https://doi.org/10.1007/s00221-012-3360-9

Chaya, M. S., Nagendra, H., Selvam, S., Kurpad, A., & Srinivasan, K. (2012). Effect of yoga on cognitive abilities in schoolchildren from a socioeconomically disadvantaged background: a randomized controlled study. Journal of alternative and complementary medicine (New York, N.Y.), 18(12), 1161-1167. https://doi.org/https://dx.doi.org/10.1089/acm.2011.0579

Chen, M. D., Tsai, H. Y., Wang, C. C., & Wuang, Y. P. (2015). The effectiveness of racket-sport intervention on visual perception and executive functions in children with mild intellectual disabilities and borderline intellectual functioning [Journal: Article]. Neuropsychiatric Disease and Treatment, 11, 2287‐2297. https://doi.org/10.2147/NDT.S89083

Chen, S. R., Tseng, C. L., Kuo, S. Y., & Chang, Y. K. (2016). Effects of a Physical Activity Intervention on Autonomic and Executive Functions in Obese Young Adolescents: A Randomized Controlled Trial. Health psychology, 35(10), 1120-1125. https://doi.org/10.1037/hea0000390

Cherriere, C. M., M. Fortin, S. Raymond, M. J. Veilleux, L. N. Lemay, M. (2020). An adapted dance program for children with Charcot-Marie-Tooth disease: An exploratory study [Article]. Journal of Bodywork and Movement Therapies, 24(2), 85-91. https://doi.org/10.1016/j.jbmt.2019.09.009

Chou, C. C., Chen, K. C., Huang, M. Y., Tu, H. Y., & Huang, C. J. (2020). Can Movement Games Enhance Executive Function in Overweight Children? A Randomized Controlled Trial. Journal of Teaching in Physical Education, 39(4), 527-535. https://doi.org/10.1123/jtpe.2019-0165

Clark, D. P., Seymour, K. E. P., Findling, R. L. M. D. M. B. A., & Mostofsky, S. H. M. D. (2020). Subtle Motor Signs as a Biomarker for Mindful Movement Intervention in Children with Attention-Deficit/Hyperactivity Disorder. Journal of Developmental and Behavioral Pediatrics, 41(5), 349. https://doi.org/http://dx.doi.org/10.1097/DBP.0000000000000795

Cofini, V. C., A. Cecilia, M. R. Carbonelli, A. Di Giacomo, D. (2021). Impact of dance therapy on children with specific learning disability: a two-arm cluster randomized control study on an Italian sample [Journal: Article]. Minerva pediatrics, 73(3), 243‐250. https://doi.org/10.23736/S2724-5276.18.05249-0

Collins, R. C., Consulting, C. M., & et al. (1994). Kaleidoscope: Profile of an Arts-Based Early Childhood Program. Final Report of an Evaluation Study [and] Technical Appendix. In.

Cooper, E. K. (2005). The effects of martial arts on inattention, impulsivity, hyperactivity and aggression in children with attention -deficit /hyperactivity disorder: A single -subject multiple -baseline design across participants (Publication Number 3174337) [Ph.D., Capella University]. ProQuest Dissertations & Theses Global. Ann Arbor. http://search.proquest.com.libraryproxy.griffith.edu.au/dissertations-theses/effects-martial-arts-on-inattention-impulsivity/docview/305357757/se-2?accountid=14543 http://hy8fy9jj4b.search.serialssolutions.com/directLink?&atitle=The+effects+of+martial+arts+on+inattention%2C+impulsivity%2C+hyperactivity+and+aggression+in+children+with+attention+-deficit+%2Fhyperactivity+disorder%3A+A+single+-subject+multiple+-baseline+design+across+participants&author=Cooper%2C+Eric+K.&issn=&title=The+effects+of+martial+arts+on+inattention%2C+impulsivity%2C+hyperactivity+and+aggression+in+children+with+attention+-deficit+%2Fhyperactivity+disorder%3A+A+single+-subject+multiple+-baseline+design+across+participants&volume=&issue=&date=2005-01-01&spage=&id=doi:&sid=ProQ_ss&genre=article

Cooper, E. K. (2006). Information & Strategies for Martial Arts Instructors. Journal of Asian Martial Arts, 15(4), 20-29. https://search.ebscohost.com/login.aspx?direct=true&db=sph&AN=23428058&site=ehost-live&scope=site

Cosper, S. M., Lee, G. P., Peters, S. B., & Bishop, E. (2009). Interactive Metronome training in children with attention deficit and developmental coordination disorders. International journal of rehabilitation research. Internationale Zeitschrift fur Rehabilitationsforschung. Revue internationale de recherches de readaptation, 32(4), 331-336. https://doi.org/https://dx.doi.org/10.1097/MRR.0b013e328325a8cf

Costa-Giomi, E. (1999). The effects of three years of piano instruction on children's cognitive development [Article]. JOURNAL OF RESEARCH IN MUSIC EDUCATION, 47(3), 198-212. https://doi.org/10.2307/3345779

Cox, E., Bells, S., Timmons, B. W., Laughlin, S., Bouffet, E., de Medeiros, C., Beera, K., Harasym, D., & Mabbott, D. J. (2020). A controlled clinical crossover trial of exercise training to improve cognition and neural communication in pediatric brain tumor survivors [Controlled Clinical Trial; Journal Article; Research Support, Non‐U.S. Gov't]. Clinical Neurophysiology, 131(7), 1533‐1547. https://doi.org/10.1016/j.clinph.2020.03.027

Cozzutti, G., Guaran, F., Blessano, E., & Romero-Naranjo, F. J. (2017). Effects on executive functions in the BAPNE Method; a study on 8-9 years old children in Friuli Venezia Giulia, Italy

Crova, C., Marchetti, R., Struzzolino, I., Forte, R., & Pesce, C. (2014). Training attention in physical education: effects on typically developing and DCD children. In J. C. O. F. M. Y. Laborda (Ed.), (Vol. 116, pp. 1509-1512). https://doi.org/10.1016/j.sbspro.2014.01.425

Crova, C., Struzzolino, I., Marchetti, R., Masci, I., Vannozzi, G., Forte, R., & Pesce, C. (2014). Cognitively challenging physical activity benefits executive function in overweight children [Journal Article; Randomized Controlled Trial]. Journal of Sports Sciences, 32(3), 201‐211. https://doi.org/10.1080/02640414.2013.828849

Cruz, K. (2017). Effects of a Randomized Trial After-School Physical Activity Club on the Math Achievement and Executive Functioning of Girls https://login.libraryproxy.griffith.edu.au/login?url=https://www.proquest.com/docview/1901929238?accountid=14543&bdid=37598&_bd=bbKBuBUH%2FOWyv7R0eREEkLaSts8%3D

Cuellar-Moreno, M. (2016). Effects of the command and mixed styles on student learning in primary education. Journal of Physical Education & Sport, 16(4), 1159-1168. https://search.ebscohost.com/login.aspx?direct=true&db=sph&AN=120826451&site=ehost-live&scope=site

Da Silva, E. R., Dos Santos Baldin, M., & Dos Santos, F. H. (2017). Cognitive effects of numeracy musical training in brazilian preschool children: A prospective pilot study [Article]. Psychology and Neuroscience, 10(3), 281-296. https://doi.org/10.1037/pne0000098

Da Silva, L. A., Doyenart, R., Henrique Salvan, P., Rodrigues, W., Felipe Lopes, J., Gomes, K., Thirupathi, A., De Pinho, R. A., & Silveira, P. C. (2019). Swimming training improves mental health parameters, cognition and motor coordination in children with Attention Deficit Hyperactivity Disorder [Journal: Article in Press]. International journal of environmental health research. https://doi.org/10.1080/09603123.2019.1612041

Dalziell, A., Booth, J. N., Boyle, J., & Mutrie, N. (2019). Better Movers and Thinkers: An evaluation of how a novel approach to teaching physical education can impact children's physical activity, coordination and cognition [Article]. British Educational Research Journal, 45(3), 576-591. https://doi.org/10.1002/berj.3514

Dalziell, A., Boyle, J., & Mutrie, N. (2015). Better movers and thinkers (BMT): An exploratory study of an innovative approach to physical education. Europe's Journal of Psychology, 11(4), 722.

Davis, C. L., Tomporowski, P. D., McDowell, J. E., Austin, B. P., Miller, P. H., Yanasak, N. E., Allison, J. D., & Naglieri, J. A. (2011). Exercise improves executive function and achievement and alters brain activation in overweight children: a randomized, controlled trial [Journal Article; Randomized Controlled Trial; Research Support, N.I.H., Extramural; Research Support, Non‐U.S. Gov't]. Health psychology, 30(1), 91‐98. https://doi.org/10.1037/a0021766

de Greeff, J. W., Hartman, E., Mullender-Wijnsma, M. J., Bosker, R. J., Doolaard, S., & Visscher, C. (2016). Long-term effects of physically active academic lessons on physical fitness and executive functions in primary school children [Journal Article; Randomized Controlled Trial; Research Support, Non‐U.S. Gov't]. Health education research, 31(2), 185‐194. https://doi.org/10.1093/her/cyv102

Degé, F., Patscheke, H., & Schwarzer, G. (2022). The influence of music training on motoric inhibition in German preschool children. MUSICAE SCIENTIAE, 26(1), 172-184.

Dege, F., Wehrum, S., Stark, R., & Schwarzer, G. (2011). The influence of two years of school music training in secondary school on visual and auditory memory [Curriculum & Programs & Teaching Methods 3530]. European Journal of Developmental Psychology, 8(5), 608-623. https://doi.org/http://dx.doi.org/10.1080/17405629.2011.590668

Dewi, E. K., Rusmawati, D., & Ratnaningsih, I. Z. (2015). The Effect of Music and Motoric Movement Intervention to Increase Attention among Elementary School Studentsin Semarang Central Java

Disanto-Rose, M. (1986). EFFECT OF CREATIVE DANCE CLASSES ON THE LEARNING OF SPATIAL CONCEPTS AND THE ABILITY TO ANALYZE SPATIAL PATHWAYS IN DANCE VIDEO BY THIRD AND FOURTH GRADE STUDENTS (Publication Number 8627443) [Educat.D., Temple University]. ProQuest Dissertations & Theses Global. Ann Arbor. http://search.proquest.com.libraryproxy.griffith.edu.au/dissertations-theses/effect-creative-dance-classes-on-learning-spatial/docview/303518719/se-2?accountid=14543 http://hy8fy9jj4b.search.serialssolutions.com/directLink?&atitle=EFFECT+OF+CREATIVE+DANCE+CLASSES+ON+THE+LEARNING+OF+SPATIAL+CONCEPTS+AND+THE+ABILITY+TO+ANALYZE+SPATIAL+PATHWAYS+IN+DANCE+VIDEO+BY+THIRD+AND+FOURTH+GRADE+STUDENTS&author=DISANTO-ROSE%2C+MARY&issn=&title=EFFECT+OF+CREATIVE+DANCE+CLASSES+ON+THE+LEARNING+OF+SPATIAL+CONCEPTS+AND+THE+ABILITY+TO+ANALYZE+SPATIAL+PATHWAYS+IN+DANCE+VIDEO+BY+THIRD+AND+FOURTH+GRADE+STUDENTS&volume=&issue=&date=1986-01-01&spage=&id=doi:&sid=ProQ_ss&genre=article

Dkang, K. D., Choi, J. W., Kang, S. G., & Han, D. H. (2011). Sports Therapy for Attention, Cognitions and Sociality. International Journal of Sports Medicine, 32(12), 953-959. https://doi.org/10.1055/s-0031-1283175

Douglas, S. (2014). Examining dance education experiences and critical thinking in urban adolescents (Publication Number 3687827) [Ed.D., Saint Mary's College of California]. ProQuest Dissertations & Theses Global. Ann Arbor. http://search.proquest.com.libraryproxy.griffith.edu.au/dissertations-theses/examining-dance-education-experiences-critical/docview/1671752506/se-2?accountid=14543 http://hy8fy9jj4b.search.serialssolutions.com/directLink?&atitle=Examining+dance+education+experiences+and+critical+thinking+in+urban+adolescents&author=Douglas%2C+Shelese&issn=&title=Examining+dance+education+experiences+and+critical+thinking+in+urban+adolescents&volume=&issue=&date=2014-01-01&spage=&id=doi:&sid=ProQ_ss&genre=article

Drollette, E. S. (2016). Physical activity for the brain, but for whom? An individual difference investigation of the FITKids clinical trial on cognitive control and ERPS in children (Publication Number 10301784) [Ph.D., University of Illinois at Urbana-Champaign]. ProQuest Dissertations & Theses Global. Ann Arbor. http://search.proquest.com.libraryproxy.griffith.edu.au/dissertations-theses/physical-activity-brain-whom-individual/docview/1857449488/se-2?accountid=14543 http://hy8fy9jj4b.search.serialssolutions.com/directLink?&atitle=Physical+activity+for+the+brain%2C+but+for+whom%3F+An+individual+difference+investigation+of+the+FITKids+clinical+trial+on+cognitive+control+and+ERPS+in+children&author=Drollette%2C+Eric+Scott&issn=&title=Physical+activity+for+the+brain%2C+but+for+whom%3F+An+individual+difference+investigation+of+the+FITKids+clinical+trial+on+cognitive+control+and+ERPS+in+children&volume=&issue=&date=2016-01-01&spage=&id=doi:&sid=ProQ_ss&genre=article

D'Souza, A. A., & Wiseheart, M. (2018). Cognitive effects of music and dance training in children [Cognitive & Perceptual Development 2820]. Archives of Scientific Psychology, 6(1), 178-192. https://doi.org/http://dx.doi.org/10.1037/arc0000048 (Special Section: Heterodox Issues in Psychology.)

Egger, F., Benzing, V., Conzelmann, A., & Schmidt, M. (2019). Boost your brain, while having a break! The effects of long-term cognitively engaging physical activity breaks on children’s executive functions and academic achievement [Article]. PLoS ONE, 14(3). https://doi.org/10.1371/journal.pone.0212482

Eom, S. L., Mi Kyung Park, Ji-Hye Lee, Dongpyo Kang, Hoon-Chul Lee, Joon Soo Jeon, Justin Y. Kim, Heung Dong. (2016). The Impact of a 35-Week Long-Term Exercise Therapy on Psychosocial Health of Children With Benign Epilepsy. Journal of Child Neurology, 31(8), 985-990. https://doi.org/https://dx.doi.org/10.1177/0883073816634859

Ericsson, I. (2008). Motor skills, attention and academic achievements. An intervention study in school years 1-3 [Article]. British Educational Research Journal, 34(3), 301-313. https://doi.org/10.1080/01411920701609299

Famelia, R. (2018). Getting an Active Start: Evaluating the Feasibility of INDO-SKIP to Promote Motor Competence, Perceived Motor Competence and Executive Function in Young, Muslim Children in Indonesia https://login.libraryproxy.griffith.edu.au/login?url=https://www.proquest.com/docview/2184251029?accountid=14543&bdid=37598&_bd=5GGVIfan233KI8bUaHIHOZYwvKM%3D

Faramarzi, S., Rad, S. A., & Abedi, A. (2016). Effect of sensory integration training on executive functions of children with attention deficit hyperactivity disorder [Journal: Article]. Neuropsychiatria i neuropsychologia, 11(1), 1‐5. https://doi.org/10.5114/nan.2016.60388

Fasano, M. C., Semeraro, C., Cassibba, R., Kringelbach, M. L., Monacis, L., e Palo, V., Vuust, P., & Brattico, E. (2019). Short-term orchestral music training modulates hyperactivity and inhibitory control in school-age children: A longitudinal behavioural study [Curriculum & Programs & Teaching Methods 3530]. FRONTIERS IN PSYCHOLOGY, 10. https://doi.org/http://dx.doi.org/10.3389/fpsyg.2019.00750

Fedewa, A. L. A., Soyeon Erwin, Heather Davis, Matthew C. (2015). A randomized controlled design investigating the effects of classroom-based physical activity on children’s fluid intelligence and achievement. School Psychology International, 36(2), 135-153. https://doi.org/10.1177/0143034314565424

Felmet, M. B. (1998). The effects of karate training on the levels of attention and impulsivity of children with attention deficit/hyperactivity disorder (Publication Number 9829263) [Ph.D., The University of Toledo]. ProQuest Dissertations & Theses Global. Ann Arbor. http://search.proquest.com.libraryproxy.griffith.edu.au/dissertations-theses/effects-karate-training-on-levels-attention/docview/304454741/se-2?accountid=14543 http://hy8fy9jj4b.search.serialssolutions.com/directLink?&atitle=The+effects+of+karate+training+on+the+levels+of+attention+and+impulsivity+of+children+with+attention+deficit%2Fhyperactivity+disorder&author=Felmet%2C+Mary+Bess&issn=&title=The+effects+of+karate+training+on+the+levels+of+attention+and+impulsivity+of+children+with+attention+deficit%2Fhyperactivity+disorder&volume=&issue=&date=1998-01-01&spage=&id=doi:&sid=ProQ_ss&genre=article

Fisher, A. B., J. M. E. Paton, J. Y. Tomporowski, P. Watson, C. McColl, J. H. Reilly, J. J. (2011). Effects of a physical education intervention on cognitive function in young children: Randomized controlled pilot study [Article]. BMC Pediatrics, 11. https://doi.org/10.1186/1471-2431-11-97

Flohr, J. W. (1996). Children's Electrophysiological Responses to Music. In.

Franceschini, S., Trevisan, P., Ronconi, L., Bertoni, S., Colmar, S., Double, K., Facoetti, A., & Gori, S. (2017). Action video games improve reading abilities and visual-to-auditory attentional shifting in English-speaking children with dyslexia [Article]. Scientific reports, 7(1), 5863. https://doi.org/10.1038/s41598-017-05826-8

Frey, A. C., François Chobert, Julie Velay, Jean-Luc Habib, Michel Besson, Mireille. (2019). Music Training Positively Influences the Preattentive Perception of Voice Onset Time in Children with Dyslexia: A Longitudinal Study. Brain Sciences, 9(4), 91. https://doi.org/http://dx.doi.org/10.3390/brainsci9040091

Frischen, U., Schwarzer, G., & Dege, F. (2019). Comparing the Effects of Rhythm-Based Music Training and Pitch-Based Music Training on Executive Functions in Preschoolers [Journal: Article]. Frontiers in integrative neuroscience, 13. https://doi.org/10.3389/fnint.2019.00041

Frischen, U., Schwarzer, G., & Dege, F. (2021). Music lessons enhance executive functions in 6-to 7-year-old children. Learning and Instruction, 74, Article 101442. https://doi.org/10.1016/j.learninstruc.2021.101442

Gall, S. A., L. Joubert, N. Ludyga, S. Müller, I. Nqweniso, S. Pühse, U. u Randt, R. Seelig, H. Smith, D. (2018). Effect of a 20-week physical activity intervention on selective attention and academic performance in children living in disadvantaged neighborhoods: a cluster randomized control trial [Journal Article; Randomized Controlled Trial; Research Support, Non‐U.S. Gov't]. PLoS ONE, 13(11), e0206908. https://doi.org/10.1371/journal.pone.0206908

Gallotta, M. C., Emerenziani, G. P., Iazzoni, S., Meucci, M., Baldari, C., & Guidetti, L. (2015). Impacts of coordinative training on normal weight and overweight/obese children’s attentional performance [Journal: Article]. Frontiers in Human Neuroscience, 9(OCTOBER). https://doi.org/10.3389/fnhum.2015.00577

Gallotta, M. C., Guidetti, L., Franciosi, E., Emerenziani, G. P., Bonavolonta, V., & Baldari, C. (2012). Effects of varying type of exertion on children's attention capacity. Medicine and Science in Sports and Exercise, 44(3), 550-555. https://doi.org/https://dx.doi.org/10.1249/MSS.0b013e3182305552

Gao, Z. L., J. E. Zeng, N. Pope, Z. C. Zhang, Y. Li, X. X. (2019). Home-Based Exergaming on Preschoolers' Energy Expenditure, Cardiovascular Fitness, Body Mass Index and Cognitive Flexibility: A Randomized Controlled Trial. Journal of Clinical Medicine, 8(10), Article 1745. https://doi.org/10.3390/jcm8101745

Garcia-Hermoso, A. H.-A., Ignacio Fernandez-Vergara, Omar Gonzalez-Calderon, Nicole Russell-Guzman, Javier Vicencio-Rojas, Francisca Chacana-Canas, Cesar Ramirez-Velez, Robinson. (2020). A before-school physical activity intervention to improve cognitive parameters in children: The Active-Start study. Scandinavian Journal of Medicine & Science in Sports, 30(1), 108-116. https://doi.org/https://dx.doi.org/10.1111/sms.13537

Georgievich Polevoy, G. (2019). The Development of the Ability to Concentrate Using Coordination Exercises for Different Types of Children. International Medical Journal, 26(3), 169-171. https://search.ebscohost.com/login.aspx?direct=true&db=c8h&AN=137024269&site=ehost-live&scope=site

Ghafori, R., Heirani, A., & Aghadsi, M. T. (2018). Effect of motor exercises on serum level of brain-derived neurotrophic factor and executive function in children with dysgraphia [Article]. Journal of Kermanshah University of Medical Sciences, 22(2). https://doi.org/10.5812/jkums.79187

Golding, A. B., Claudia Nordin-Bates, Sanna M. (2016). Investigating learning through developmental dance movement as a kinaesthetic tool in the Early Years Foundation Stage [Article]. Research in Dance Education, 17(3), 235-267. https://doi.org/10.1080/14647893.2016.1204282

Golos, A., Sarid, M., Weill, M., & Weintraub, N. (2011). Efficacy of an Early Intervention Program for At-Risk Preschool Boys: A Two-Group Control Study. American Journal of Occupational Therapy, 65(4), 400-408. https://doi.org/10.5014/ajot.2011.000455

Granacher, U., & Borde, R. (2017). Effects of sport-specific training during the early stages of long-term athlete development on physical fitness, body composition, cognitive, and academic performances [Article]. FRONTIERS IN PHYSIOLOGY, 8(OCT). https://doi.org/10.3389/fphys.2017.00810

Gray, S. I. (2017). Developing and evaluating the feasibility of an active training game for smart-phones as a tool for promoting executive function in children https://login.libraryproxy.griffith.edu.au/login?url=https://www.proquest.com/docview/1999216325?accountid=14543&bdid=37598&_bd=c%2BDdcaM4PKp7DBO1crY88ws3%2BPU%3D

Greco, G., & De Ronzi, R. (2020). Effect of Karate training on social, emotional, and executive functioning in children with autism spectrum disorder. Journal of Physical Education & Sport, 20(4), 1637-1645. http://libraryproxy.griffith.edu.au/login?url=https://search.ebscohost.com/login.aspx?direct=true&db=s3h&AN=146868575&site=ehost-live&scope=site

Gromko, J. E. P., Allison Smith. (1998). The effect of music training on preschoolers' spatial-temporal task performance [Curriculum & Programs & Teaching Methods 3530]. JOURNAL OF RESEARCH IN MUSIC EDUCATION, 46(2), 173-181. https://doi.org/http://dx.doi.org/10.2307/3345621

Gross, W., Linden, U., & Ostermann, T. (2010). Effects of music therapy in the treatment of children with delayed speech development - results of a pilot study. BMC COMPLEMENTARY AND ALTERNATIVE MEDICINE, 10. https://doi.org/10.1186/1472-6882-10-39

Guo, X., Ohsawa, C., Suzuki, A., & Sekiyama, K. (2018). Improved digit span in children after a 6-week intervention of playing a musical instrument: An exploratory randomized controlled trial [Developmental Psychology 2800]. FRONTIERS IN PSYCHOLOGY, 8. https://doi.org/http://dx.doi.org/10.3389/fpsyg.2017.02303

Habibi, A., Damasio, A., Ilari, B., Sachs, M. E., & Damasio, H. (2018). Music training and child development: a review of recent findings from a longitudinal study. ANNALS OF THE NEW YORK ACADEMY OF SCIENCES, 1423(1), 73-81. https://doi.org/10.1111/nyas.13606

Hallberg, K. A., Martin, W. E., & McClure, J. R. (2017). The impact of music instruction on attention in kindergarten children [Curriculum & Programs & Teaching Methods 3530]. Psychomusicology: Music, Mind, and Brain, 27(2), 113-121. https://doi.org/http://dx.doi.org/10.1037/pmu0000177 (Psychomusicology: A Journal of Research in Music Cognition, Psychomusicology: Music, Mind and Brain)

Halperin, J. M., Marks, D. J., Chacko, A., Bedard, A. C., O'Neill, S., Curchack-Lichtin, J., Bourchtein, E., & Berwid, O. G. (2020). Training Executive, Attention, and Motor Skills (TEAMS): a Preliminary Randomized Clinical Trial of Preschool Youth with ADHD [Journal Article; Randomized Controlled Trial; Research Support, N.I.H., Extramural]. Journal of Abnormal Child Psychology, 48(3), 375‐389. https://doi.org/10.1007/s10802-019-00610-w

Hanson, M. K. (2001). An investigation of the effects of sequenced Kodály literacy-based music instruction on the spatial reasoning skills of kindergarten students (Publication Number 1406182) [M.A., University of St. Thomas (Minnesota)]. ProQuest Dissertations & Theses Global. Ann Arbor. http://search.proquest.com.libraryproxy.griffith.edu.au/dissertations-theses/investigation-effects-sequenced-kodály-literacy/docview/304770407/se-2?accountid=14543 http://hy8fy9jj4b.search.serialssolutions.com/directLink?&atitle=An+investigation+of+the+effects+of+sequenced+Kod%C3%A1ly+literacy-based+music+instruction+on+the+spatial+reasoning+skills+of+kindergarten+students&author=Hanson%2C+Marlene+Kay&issn=&title=An+investigation+of+the+effects+of+sequenced+Kod%C3%A1ly+literacy-based+music+instruction+on+the+spatial+reasoning+skills+of+kindergarten+students&volume=&issue=&date=2001-01-01&spage=&id=doi:&sid=ProQ_ss&genre=article

Harbourne, R. T., & Berger, S. E. (2019). Embodied Cognition in Practice: exploring Effects of a Motor-Based Problem-Solving Intervention [Journal Article; Randomized Controlled Trial; Research Support, Non‐U.S. Gov't]. Physical Therapy, 99(6), 786‐796. https://doi.org/10.1093/ptj/pzz031

Harris, H. B. (2016). The Impact of Technology-Enhanced Classroom Physical Activity Interventions on Executive Function, Motivation, and Physical Fitness https://login.libraryproxy.griffith.edu.au/login?url=https://www.proquest.com/docview/1874475099?accountid=14543&bdid=37598&_bd=wMIQP675TJajfBb6RvBPg40D4F0%3D

Have, M., Nielsen, J. H., Ernst, M. T., Gejl, A. K., Fredens, K., Grøntved, A., & Kristensen, P. L. (2018). Classroom-based physical activity improves children's math achievement - A randomized controlled trial [Journal article]. PLoS ONE, 13(12), e0208787. https://doi.org/10.1371/journal.pone.0208787

Hecht, M. F. (2020). Increasing Physical Activity in Elementary School Classrooms https://login.libraryproxy.griffith.edu.au/login?url=https://www.proquest.com/docview/2418797061?accountid=14543&bdid=37598&_bd=exDUz3o3oztSug5cA7lQJAJEpJw%3D

Hecht, M. F., & Garber, C. E. (2021). Effectiveness of the POWER Program in Improving Physical Activity and Executive Function in Fifth Grade Students. Journal of School Health, 91(7), 574-583. https://doi.org/10.1111/josh.13035

Hedayati, N., Schibli, K., & D'Angiulli, A. (2016). El Sistema-inspired ensemble music training is associated with changes in children's neurocognitive functional integration: Preliminary ERP evidence [Electrophysiology 2530]. Neurocase, 22(6), 538-547. https://doi.org/http://dx.doi.org/10.1080/13554794.2016.1241885

Hedayatjoo, M. R., M. Zarei, M. A. Mirzakhany, N. Nazeri, A. Baghban, A. A. Hedayatjoo, Z. Dezfoly, R. M. (2020). Effect of balance training on balance performance, motor coordination, and attention in children with hearing deficits [Article]. Archives of Neuroscience, 7(1). https://doi.org/10.5812/ans.84869

Hendry, J. K., R. (1983). Communication through physical activity for learning disabled children [Article]. Perceptual and Motor Skills, 56(1), 155-158. https://doi.org/10.2466/pms.1983.56.1.155

Hill, L., Williams, J. H., Aucott, L., Milne, J., Thomson, J., Greig, J., Munro, V., & Mon-Williams, M. (2010). Exercising attention within the classroom [Journal Article; Randomized Controlled Trial; Research Support, Non‐U.S. Gov't]. Developmental Medicine and Child Neurology, 52(10), 929‐934. https://doi.org/10.1111/j.1469-8749.2010.03661.x

Hilton, C. L., Cumpata, K., Klohr, C., Gaetke, S., Artner, A., Johnson, H., & Dobbs, S. (2014). Effects of Exergaming on Executive Function and Motor Skills in Children With Autism Spectrum Disorder: A Pilot Study. American Journal of Occupational Therapy, 68(1), 57-65. https://doi.org/10.5014/ajot.2014.008664

Holochwost, S. J., Propper, C. B., Wolf, D. P., Willoughby, M. T., Fisher, K. R., Kolacz, J., Volpe, V. V., & Jaffee, S. R. (2017). Music Education, Academic Achievement, and Executive Functions. Psychology of Aesthetics Creativity and the Arts, 11(2), 147-166. https://doi.org/10.1037/aca0000112

Howard, S. J., Vasseleu, E., Batterham, M., & Neilsen-Hewett, C. (2020). Everyday Practices and Activities to Improve Pre-school Self-Regulation: Cluster RCT Evaluation of the PRSIST Program. FRONTIERS IN PSYCHOLOGY, 11, Article 137. https://doi.org/10.3389/fpsyg.2020.00137

Hsieh, S.-S. L., Chih-Chien Chang, Yu-Kai Huang, Chung-Ju Hung, Tsung-Min. (2017). Effects of Childhood Gymnastics Program on Spatial Working Memory. Medicine and Science in Sports and Exercise, 49(12), 2537-2547. https://doi.org/https://dx.doi.org/10.1249/MSS.0000000000001399

Hudson, K. N., Ballou, H. M., & Willoughby, M. T. (2021). Short report: Improving motor competence skills in early childhood has corollary benefits for executive function and numeracy skills. Developmental science, 24(4), Article e13071. https://doi.org/10.1111/desc.13071

Ilari, B., Helfter, S., Huynh, T., Bowmer, A., Mason, K., Knight, J., & Welch, G. (2021). Musical Activities, Prosocial Behaviors, and Executive Function Skills of Kindergarten Children. Music & Science, 4. https://login.libraryproxy.griffith.edu.au/login?url=https://www.proquest.com/docview/2613208183?accountid=14543&bdid=37599&_bd=Xwevs23nzc0UOMGnoNRbP3IF6RM%3D

Ishihara, T., & Mizuno, M. (2018). Effects of tennis play on executive function in 6-11-year-old children: a 12-month longitudinal study. European Journal of Sport Science, 18(5), 741-752. https://doi.org/10.1080/17461391.2018.1444792

Ishihara, T., Sugasawa, S., Matsuda, Y., & Mizuno, M. (2017). Improved executive functions in 6-12-year-old children following cognitively engaging tennis lessons [Article]. Journal of Sports Sciences, 35(20), 2014-2020. https://doi.org/10.1080/02640414.2016.1250939

Jacob, U. S., Pillay, J., & Oyefeso, E. O. (2021). Attention Span of Children With Mild Intellectual Disability: Does Music Therapy and Pictorial Illustration Play Any Significant Role? FRONTIERS IN PSYCHOLOGY, 12. https://doi.org/10.3389/fpsyg.2021.677703

Jaksic, D. M., S. Maksimovic, N. Milosevic, Z. Roklicer, R. Vukovic, J. Pocek, S. Lakicevic, N. Bianco, A. Cassar, S. Drid, P. (2020). Effects of a nine-month physical activity intervention on morphological characteristics and motor and cognitive skills of preschool children [Article]. International Journal of Environmental Research and Public Health, 17(18), 1-11. https://doi.org/10.3390/ijerph17186609

James, C. E., Zuber, S., Dupuis-Lozeron, E., Abdili, L., Gervaise, D., & Kliegel, M. (2020). Formal string instrument training in a class setting enhances cognitive and sensorimotor development of primary school children. Frontiers in Neuroscience, 14, 510563.

Jaschke, A. C., Honing, H., & Scherder, E. J. A. (2018). Longitudinal Analysis of Music Education on Executive Functions in Primary School Children. Frontiers in Neuroscience, 12, Article 103. https://doi.org/10.3389/fnins.2018.00103

Jeyanthi, S., Arumugam, N., & Parasher, R. K. (2021). Effectiveness of structured exercises on motor skills, physical fitness and attention in children with ADHD compared to typically developing children-A pilot study [Article]. eNeurologicalSci, 24. https://doi.org/10.1016/j.ensci.2021.100357

Johnson, D. C., & Davis, V. W. (2016). The Effects of Musical Ensembles-in-Residence on Elementary Students' Auditory Discrimination and Spatial Reasoning Skills: A Longitudinal Study [Article]. Visions of Research in Music Education, 28, 1-28. https://search.ebscohost.com/login.aspx?direct=true&db=eue&AN=122407670&site=ehost-live&scope=site

Kamijo, K. P., M. B. O'Leary, K. C. Scudder, M. R. Wu, C. T. Castelli, D. M. Hillman, C. H. (2011). The effects of an afterschool physical activity program on working memory in preadolescent children [Journal Article; Randomized Controlled Trial; Research Support, N.I.H., Extramural; Research Support, Non‐U.S. Gov't]. Developmental science, 14(5), 1046‐1058. https://doi.org/10.1111/j.1467-7687.2011.01054.x

Kanitz, J. L., Pretzer, K., Calaminus, G., Wiener, A., Längler, A., Henze, G., Driever, P. H., & Seifert, G. (2013). Eurythmy therapy in the aftercare of pediatric posterior fossa tumour survivors--A pilot study. Complementary Therapies in Medicine, 21(S1), S3-9. https://doi.org/http://dx.doi.org/10.1016/j.ctim.2012.02.007

Kashfi, T. E., Sohrabi, M., Kakhki, A. S., Mashhadi, A., & Nooghabi, M. J. (2019). Effects of a Motor Intervention Program on Motor Skills and Executive Functions in Children With Learning Disabilities. Perceptual and Motor Skills, 126(3), 477-498. https://doi.org/10.1177/0031512519836811

Kaviani, H., Mirbaha, H., Pournaseh, M., & Sagan, O. (2014). Can music lessons increase the performance of preschool children in IQ tests? [Article]. Cognitive Processing, 15(1), 77-84. https://doi.org/10.1007/s10339-013-0574-0

Kayili, G. K., Özden. (2020). The effects of Orff-based attention-enhancing music education programme on impulsive preschool children's cognitive tempo [Article]. Early Child Development & Care, 190(3), 390-399. https://doi.org/10.1080/03004430.2018.1475367

Keiver, K., Pritchard Orr, A., Schneider, A. L. J., Golubovich, N., Reynolds, J., & Graham, T. C. N. (2019). Effect of the liberi exergamingprogram on executive function abilities in children with fetal alcohol spectrum disorder: A pilot study [Conference Abstract]. Alcoholism: Clinical and Experimental Research, 43, 227A. https://doi.org/10.1111/acer.14059

Khondowe, O., Nikodem, V. C., Frantz, J. M., & Harper, K. (2015). A physical activity programme to improve motor and cognitive development in HIV positive children on antiretroviral therapy: A randomised controlled trial. African Journal for Physical, Health Education, Recreation & Dance, 21(4:1), 1187-1199. https://search.ebscohost.com/login.aspx?direct=true&db=sph&AN=119182051&site=ehost-live&scope=site

Kim, H. B., & So, W. Y. (2015). EFFECT OF SIXTEEN WEEKS OF COMBINED EXERCISE ON BODY COMPOSITION, PHYSICAL FITNESS AND COGNITIVE FUNCTION IN KOREAN CHILDREN. South African Journal for Research in Sport Physical Education and Recreation, 37(1), 47-57. <Go to ISI>://WOS:000359390400004

Knight, N. A. (2015). Effects of a Before School Physical Activity Program on Physical Activity, Musculoskeletal Fitness, and Cognitive Function (Publication Number 1590117) [M.S., East Carolina University]. ProQuest Dissertations & Theses Global. Ann Arbor. http://search.proquest.com.libraryproxy.griffith.edu.au/dissertations-theses/effects-before-school-physical-activity-program/docview/1693995795/se-2?accountid=14543 http://hy8fy9jj4b.search.serialssolutions.com/directLink?&atitle=Effects+of+a+Before+School+Physical+Activity+Program+on+Physical+Activity%2C+Musculoskeletal+Fitness%2C+and+Cognitive+Function&author=Knight%2C+Noelle+A.&issn=&title=Effects+of+a+Before+School+Physical+Activity+Program+on+Physical+Activity%2C+Musculoskeletal+Fitness%2C+and+Cognitive+Function&volume=&issue=&date=2015-01-01&spage=&id=doi:&sid=ProQ_ss&genre=article

Kosokabe, T., Mizusaki, M., Nagaoka, W., Honda, M., Suzuki, N., Naoi, R., & Moriguchi, Y. (2021). Self-directed dramatic and music play programs enhance executive function in Japanese children. TRENDS IN NEUROSCIENCE AND EDUCATION, 24. https://doi.org/10.1016/j.tine.2021.100158

Kouhbanani, S. S. A., S. M. Zarenezhad, S. Khosrorad, R. (2020). The effect of perceptual-motor training on executive functions in children with non-verbal learning disorder [Article]. Neuropsychiatric Disease and Treatment, 16, 1129-1137. https://doi.org/10.2147/NDT.S252662

Krafft, C. E. S., D. J. Schwarz, N. F. Chi, L. Weinberger, A. L. Pierce, J. E. Rodrigue, A. L. Allison, J. D. Yanasak, N. E. Liu, T. (2014). Improved frontoparietal white matter integrity in overweight children is associated with attendance at an after-school exercise program [Journal Article; Randomized Controlled Trial; Research Support, N.I.H., Extramural; Research Support, U.S. Gov't, Non‐P.H.S.]. Developmental neuroscience, 36(1), 1‐9. https://doi.org/10.1159/000356219

Kvalo, S. E., Bru, E., Bronnick, K., & Dyrstad, S. M. (2017). Does increased physical activity in school affect children's executive function and aerobic fitness? Scandinavian Journal of Medicine & Science in Sports, 27(12), 1833-1841. https://doi.org/10.1111/sms.12856

LaGasse, A. B. (2019). Assessing the Impact of Music Therapy on Sensory Gating and Attention in Children With Autism: A Pilot and Feasibility Study. JOURNAL OF MUSIC THERAPY, 56(3), 287-314. https://doi.org/http://dx.doi.org/10.1093/jmt/thz008

Lai, Y. J. C., K. M. (2020). Improvement of attention in elementary school students through fixation focus training activity [Article]. International Journal of Environmental Research and Public Health, 17(13), 1-13. https://doi.org/10.3390/ijerph17134780

Lakes, K. D., Bryars, T., Sirisinahal, S., Salim, N., Arastoo, S., Emmerson, N., Kang, D., Shim, L., Wong, D., & Kang, C. J. (2013). The Healthy for Life Taekwondo pilot study: A preliminary evaluation of effects on executive function and BMI, feasibility, and acceptability. Mental Health and Physical Activity, 6(3), 181-188. https://doi.org/10.1016/j.mhpa.2013.07.002

Lakes, K. D., & Hoyt, W. I. (2004). Promoting self-regulation through school-based martial arts training. Journal of Applied Developmental Psychology, 25(3), 283-302. https://doi.org/10.1016/j.appdev.2004.04.002

Lakes, K. D. S., K. Grant-Beuttler, M. Neville, R. Haddad, F. Sunico, R. Ho, D. Schneider, M. Sawitz, S. Paulsen, J. Caputo, K. Lu, K. D. Aminian, A. Lopez-Ortiz, C. Radom-Aizik, S. (2019). A Six Week Therapeutic Ballet Intervention Improved Gait and Inhibitory Control in Children With Cerebral Palsy-A Pilot Study. Frontiers in Public Health, 7, Article 137. https://doi.org/10.3389/fpubh.2019.00137

Lau, M. T. E. (2017). Treatment Effects of Chan-based Mind Body Exercise and Progressive Muscle Relaxation on Impulse Control and Cognitive Flexibility of Children with Autism Spectrum Disorder: A Randomized Controlled Trial (Publication Number 10672408) [Ph.D., The Chinese University of Hong Kong (Hong Kong)]. ProQuest Dissertations & Theses Global. Ann Arbor. http://search.proquest.com.libraryproxy.griffith.edu.au/dissertations-theses/treatment-effects-chan-based-mind-body-exercise/docview/1994437640/se-2?accountid=14543 http://hy8fy9jj4b.search.serialssolutions.com/directLink?&atitle=Treatment+Effects+of+Chan-based+Mind+Body+Exercise+and+Progressive+Muscle+Relaxation+on+Impulse+Control+and+Cognitive+Flexibility+of+Children+with+Autism+Spectrum+Disorder%3A+A+Randomized+Controlled+Trial&author=Lau%2C+Mei+Ting+Eliza&issn=&title=Treatment+Effects+of+Chan-based+Mind+Body+Exercise+and+Progressive+Muscle+Relaxation+on+Impulse+Control+and+Cognitive+Flexibility+of+Children+with+Autism+Spectrum+Disorder%3A+A+Randomized+Controlled+Trial&volume=&issue=&date=2017-01-01&spage=&id=doi:&sid=ProQ_ss&genre=article

Layne, T., Yli-Piipari, S., & Knox, T. (2021). Physical activity break program to improve elementary students' executive function and mathematics performance [Article]. Education 3-13, 49(5), 583-591. https://doi.org/10.1080/03004279.2020.1746820

Lee, J., Zhang, T., Chu, T. L., Gu, X., & Zhu, P. (2020). Effects of a fundamental motor skill-based afterschool program on children’s physical and cognitive health outcomes [Article]. International Journal of Environmental Research and Public Health, 17(3). https://doi.org/10.3390/ijerph17030733

Lee, S. K., Song, J., & Park, J. H. (2017). Effects of combination exercises on electroencephalography and frontal lobe executive function measures in children with ADHD: A pilot study [Article]. Biomedical Research (India), 2017(Special Issue HealthScienceandBioConvergenceTechnologyEdition-II), S455-S460. https://www.embase.com/search/results?subaction=viewrecord&id=L621285467&from=export

Leth-Steensen, C., Gallitto, E., Haghbin, M., & Hannan, P. A. (2021). Enhancing the Cognitive and Motor Abilities of very Young Children: A Pilot Study of the Efficacy of the PlayWisely Approach. Mind Brain and Education, 15(1), 103-110. https://doi.org/10.1111/mbe.12269

Li, M. H., Sit, C. H. P., Wong, S. H. S., Wing, Y. K., Ng, C. K., & Sum, R. K. W. (2021). Promoting physical activity and health in Hong Kong primary school children through a blended physical literacy intervention: protocol and baseline characteristics of the "Stand+Move" randomized controlled trial [Clinical Trial Protocol]. Trials [Electronic Resource], 22(1), 944. https://libraryproxy.griffith.edu.au/login?url=https://ovidsp.ovid.com/ovidweb.cgi?T=JS&CSC=Y&NEWS=N&PAGE=fulltext&D=med20&AN=34930404 https://griffithuni.on.worldcat.org/atoztitles/link?&rft_val_fmt=info:ofi/fmt:kev:mtx:journal&rfr_id=info:sid/Ovid:med20&rft.genre=article&rft_id=info:doi/10.1186%2Fs13063-021-05925-y&rft_id=info:pmid/34930404&rft.issn=1745-6215&rft.volume=22&rft.issue=1&rft.spage=944&rft.pages=944&rft.date=2021&rft.jtitle=Trials+%5BElectronic+Resource%5D&rft.atitle=Promoting+physical+activity+and+health+in+Hong+Kong+primary+school+children+through+a+blended+physical+literacy+intervention%3A+protocol+and+baseline+characteristics+of+the+%22Stand%2BMove%22+randomized+controlled+trial.&rft.aulast=Li

Li, Y. H. C., D. Ransdell, M. Coleman, L. Irwin, C. (2011). SPORT STACKING ACTIVITIES IN SCHOOL CHILDREN'S MOTOR SKILL DEVELOPMENT. Perceptual and Motor Skills, 113(2), 431-438. https://doi.org/10.2466/05.06.10.11.25.Pms.113.5.431-438

Lima, R. F., Da Silva, V. F., De Oliveira, G. L., De Oliveira, T. A. P., Filho, J. F., MendonÇA, J. G. R., Borges, C. J., MilitÃO, A. G., De Aquino Freire, I., & Valentim-Silva, J. R. (2017). Practicing karate may improves executive functions of 8-11-year-old schoolchildren. Journal of Physical Education & Sport, 17(4), 2513-2518. https://search.ebscohost.com/login.aspx?direct=true&db=sph&AN=129264104&site=ehost-live&scope=site

Lin, C.-C., Hsieh, S.-S., Chang, Y.-K., Huang, C.-J., Hillman, C. H., & Hung, T.-M. (2021). Up-regulation of proactive control is associated with beneficial effects of a childhood gymnastics program on response preparation and working memory. Brain & Cognition, 149, N.PAG-N.PAG. https://doi.org/10.1016/j.bandc.2021.105695

Lind, R. R. G., S. S. Orntoft, C. Madsen, M. Larsen, M. N. Dvorak, J. Ritz, C. Krustrup, P. (2018). Improved cognitive performance in preadolescent Danish children after the school-based physical activity programme "FIFA 11 for Health" for Europe - A cluster-randomised controlled trial [Article]. European Journal of Sport Science, 18(1), 130‐139. https://doi.org/10.1080/17461391.2017.1394369

Linnavalli, T., Putkinen, V., Lipsanen, J., Huotilainen, M., & Tervaniemi, M. (2018). Music playschool enhances children's linguistic skills [Article]. Scientific reports, 8(1), 8767. https://doi.org/10.1038/s41598-018-27126-5

lisahunter, A., Rebecca, M., Doune, Z., & Jennifer, C. (2014). Active Kids Active Minds: A Physical Activity Intervention to Promote Learning? Asia-Pacific Journal of Health, Sport and Physical Education, 5(2), 117-131. https://search.ebscohost.com/login.aspx?direct=true&db=eric&AN=EJ1031533&site=ehost-live&scope=site http://dx.doi.org/10.1080/18377122.2014.906057

Ludyga, S., Kochli, S., Puhse, U., Gerber, M., & Hanssen, H. (2018). Effects of a school-based physical activity program on retinal microcirculation and cognitive function in adolescents [Journal: Article in Press]. Journal of Science and Medicine in Sport. https://doi.org/10.1016/j.jsams.2018.11.029

Ludyga, S., Koutsandreou, F., Reuter, E. M., Voelcker-Rehage, C., & Budde, H. (2019). A randomized controlled trial on the effects of aerobic and coordinative training on neural correlates of inhibitory control in children [Journal: Article]. Journal of Clinical Medicine, 8(2). https://doi.org/10.3390/jcm8020184

Ludyga, S., Trankner, S., Gerber, M., & Puhse, U. (2021). Effects of Judo on Neurocognitive Indices of Response Inhibition in Preadolescent Children: A Randomized Controlled Trial. Medicine and Science in Sports and Exercise, 53(8), 1648-1655. https://doi.org/10.1249/mss.0000000000002626

MacCutcheon, D., Fullgrabe, C., Eccles, R., van der Linde, J., Panebianco, C., & Ljung, R. (2020). Investigating the effect of one year of learning to play a musical instrument on speech-in-noise perception and phonological short-term memory in 5-to-7-year-old children [Cognitive & Perceptual Development 2820]. FRONTIERS IN PSYCHOLOGY, 10. https://doi.org/http://dx.doi.org/10.3389/fpsyg.2019.02865

Marson, F. F., Antonio De Pellegrino, Michele Pesce, Caterina Glicksohn, Joseph Ben-Soussan, Tal Dotan. (2021). Age-Related Differential Effects of School-Based Sitting and Movement Meditation on Creativity and Spatial Cognition: A Pilot Study. Children, 8(7), 1-19. https://doi.org/10.3390/children8070583

Mason, K., Marshall, C. R., & Morgan, G. (2021). Executive Function Training for Deaf Children: Impact of a Music Intervention [Research Support, Non-U.S. Gov't]. Journal of Deaf Studies & Deaf Education, 26(4), 490-500. https://libraryproxy.griffith.edu.au/login?url=https://ovidsp.ovid.com/ovidweb.cgi?T=JS&CSC=Y&NEWS=N&PAGE=fulltext&D=med20&AN=34476479 https://griffithuni.on.worldcat.org/atoztitles/link?&rft_val_fmt=info:ofi/fmt:kev:mtx:journal&rfr_id=info:sid/Ovid:med20&rft.genre=article&rft_id=info:doi/10.1093%2Fdeafed%2Fenab026&rft_id=info:pmid/34476479&rft.issn=1081-4159&rft.volume=26&rft.issue=4&rft.spage=490&rft.pages=490-500&rft.date=2021&rft.jtitle=Journal+of+Deaf+Studies+%26+Deaf+Education&rft.atitle=Executive+Function+Training+for+Deaf+Children%3A+Impact+of+a+Music+Intervention.&rft.aulast=Mason

Mason, K. C. (2017). Executive Function in Deaf Children : an Intervention Study https://login.libraryproxy.griffith.edu.au/login?url=https://www.proquest.com/docview/2636121059?accountid=14543&bdid=37599&_bd=eS4DSedaAK0XbOFlc0Mma7%2FZq8w%3D

Mavilidi, M. F., Lubans, D. R., Eather, N., Morgan, P. J., & Riley, N. (2018). Preliminary efficacy and feasibility of “Thinking While Moving in English”: A program with physical activity integrated into primary school English lessons. Children, 5(8), 109.

Mavilidi, M. F., Lubans, D. R., Morgan, P. J., Miller, A., Eather, N., Karayanidis, F., Lonsdale, C., Noetel, M., Shaw, K., & Riley, N. (2019). Integrating physical activity into the primary school curriculum: rationale and study protocol for the "Thinking while Moving in English" cluster randomized controlled trial. BMC PUBLIC HEALTH, 19, Article 379. https://doi.org/10.1186/s12889-019-6635-2

Mavilidi, M. F., Okely, A. D., Chandler, P., Cliff, D. P., & Paas, F. (2015). Effects of Integrated Physical Exercises and Gestures on Preschool Children's Foreign Language Vocabulary Learning. Educational Psychology Review, 27(3), 413-426. https://doi.org/10.1007/s10648-015-9337-z

Mavilidi, M. F. D., Ryan Morgan, Philip J. Lubans, David R. Schmidt, Mirko Riley, Nicholas. (2020). Effects of different types of classroom physical activity breaks on children's on-task behaviour, academic achievement and cognition. Acta paediatrica (Oslo, Norway : 1992), 109(1), 158-165. https://doi.org/https://dx.doi.org/10.1111/apa.14892 (Comment in: Acta Paediatr. 2020 Jan;109(1):14-16; PMID: 31692046 [https://www.ncbi.nlm.nih.gov/pubmed/31692046])

Mazzoli, E., Salmon, J., Pesce, C., Teo, W., P, R., N, M., & T, B. (2021). Effects of classroom‐based active breaks on cognition, sitting and on‐task behaviour in children with intellectual disability: a pilot study. Journal of Intellectual Disability Research, 65(5), 464-488. https://doi.org/10.1111/jir.12826

Mazzoli, E., Salmon, J., Teo, W. P., Pesce, C., He, J., Ben-Soussan, T. D., & Barnett, L. M. (2021). Breaking up classroom sitting time with cognitively engaging physical activity: behavioural and brain responses [Journal: Article]. PLoS ONE, 16(7 July). https://doi.org/10.1371/journal.pone.0253733

McCullogh, N. (2019). The Efficacy of School-Based Classroom Learning and Physical Activity Interventions for Children’s Cognitive Performance and Wellbeing (Publication Number 27992034) [Ph.D., University of Northumbria at Newcastle (United Kingdom)]. ProQuest Dissertations & Theses Global. Ann Arbor. http://search.proquest.com.libraryproxy.griffith.edu.au/dissertations-theses/efficacy-school-based-classroom-learning-physical/docview/2425018835/se-2?accountid=14543 http://hy8fy9jj4b.search.serialssolutions.com/directLink?&atitle=The+Efficacy+of+School-Based+Classroom+Learning+and+Physical+Activity+Interventions+for+Children%E2%80%99s+Cognitive+Performance+and+Wellbeing&author=McCullogh%2C+Nicola&issn=&title=The+Efficacy+of+School-Based+Classroom+Learning+and+Physical+Activity+Interventions+for+Children%E2%80%99s+Cognitive+Performance+and+Wellbeing&volume=&issue=&date=2019-01-01&spage=&id=doi:&sid=ProQ_ss&genre=article http://nrl.northumbria.ac.uk/41886/

Mehibe, A., Gülsüm, B., Recep, A., & Kahraman, B. (2010). THE EFFECT OF EDUCATIONAL GAME OVER ATTENTION IN CHILDREN. Ovidius University Annals, Series Physical Education & Sport/Science, Movement & Health, 10(2), 325-329. https://search.ebscohost.com/login.aspx?direct=true&db=sph&AN=54581214&site=ehost-live&scope=site

Memarmoghaddam, M., Torbati, H. T., Sohrabi, M., Mashhadi, A., & Kashi, A. (2016). Effects of a selected exercise programon executive function of children with attention deficit hyperactivity disorder [Journal Article; Randomized Controlled Trial]. Journal of medicine and life, 9(4), 373‐379. https://www.cochranelibrary.com/central/doi/10.1002/central/CN-01337075/full

Mierau, A., Hülsdünker, T., Mierau, J., Hense, A., Hense, J., & Strüder, H. K. (2014). Acute exercise induces cortical inhibition and reduces arousal in response to visual stimulation in young children [Journal Article; Randomized Controlled Trial]. International Journal of Developmental Neuroscience, 34, 1‐8. https://doi.org/10.1016/j.ijdevneu.2013.12.009

Mihai-Adrian, S. (2019). Study on Improving Distributive Attention by Using Movement Games Third Grade Students. / Studiu Privind Îmbunătăţirea Atenţiei Distributive prin Folosirea Jocurilor de Mişcare la Elevii de Clasa A III-A. Gymnasium: Scientific Journal of Education, Sports & Health, 20(2), 148-161. https://search.ebscohost.com/login.aspx?direct=true&db=sph&AN=146020599&site=ehost-live&scope=site

Milajerdi, H. R., Sheikh, M., Najafabadi, M. G., Saghaei, B., Naghdi, N., & Dewey, D. (2021). The Effects of Physical Activity and Exergaming on Motor Skills and Executive Functions in Children with Autism Spectrum Disorder. Games for health journal, 10(1), 33-42. https://doi.org/10.1089/g4h.2019.0180

Mireille, J. C. M. A., Eva, D., Hondt, L., Alexander, L. V., Acker, D. G., Ann, L., Matthieu, D., & Frederik J. A, C. (2019). Weight loss, behavioral change, and structural neuroplasticity in children with obesity through a multidisciplinary treatment program. Human Brain Mapping, 40(1), 137-150. https://login.libraryproxy.griffith.edu.au/login?url=https://www.proquest.com/docview/2154064383?accountid=14543&bdid=37598&_bd=QQXzPsMln5XHzyv8%2BkTtZJSsi2s%3D

Mitts, M. (2018). Effect of Sensory Stimulation in Physical Activity on Academic Achievement and Classroom Behavior in Elementary Students ProQuest LLC]. eric. https://search.ebscohost.com/login.aspx?direct=true&db=eric&AN=ED591381&site=ehost-live&scope=site http://gateway.proquest.com/openurl?url_ver=Z39.88-2004&rft_val_fmt=info:ofi/fmt:kev:mtx:dissertation&res_dat=xri:pqm&rft_dat=xri:pqdiss:10936553

Mohanty, B., & Hejmadi, A. (1992). Effects of intervention training on some cognitive abilities of preschool children [Curriculum & Programs & Teaching Methods 3530]. Psychological Studies, 37(1), 31-37. https://ovidsp.ovid.com/ovidweb.cgi?T=JS&PAGE=reference&D=psyc3&NEWS=N&AN=1993-15394-001

Monti, J. M., Hillman, C. H., & Cohen, N. J. (2012). Aerobic fitness enhances relational memory in preadolescent children: the FITKids randomized control trial [Journal Article; Randomized Controlled Trial; Research Support, N.I.H., Extramural]. Hippocampus, 22(9), 1876‐1882. https://doi.org/10.1002/hipo.22023

Moreau, D., Kirk, I. J., & Waldie, K. E. (2017). High-intensity training enhances executive function in children in a randomized, placebo-controlled trial [Journal Article; Multicenter Study; Randomized Controlled Trial]. Elife, 6. https://doi.org/10.7554/eLife.25062

Moreno, S., Bialystok, E., Barac, R., Schellenberg, E. G., Cepeda, N. J., & Chau, T. (2011). Short-Term Music Training Enhances Verbal Intelligence and Executive Function. Psychological Science (0956-7976), 22(11), 1425-1433. https://search.ebscohost.com/login.aspx?direct=true&db=sph&AN=67745918&site=ehost-live&scope=site

Moreno Sala, M. T. (2005). The influence of perceptual shift, cognitive abilities and environmental factors on young children's development of absolute and relative pitch perception (Publication Number NR21678) [Ph.D., McGill University (Canada)]. ProQuest Dissertations & Theses Global. Ann Arbor. http://search.proquest.com.libraryproxy.griffith.edu.au/dissertations-theses/influence-perceptual-shift-cognitive-abilities/docview/305375111/se-2?accountid=14543 http://hy8fy9jj4b.search.serialssolutions.com/directLink?&atitle=The+influence+of+perceptual+shift%2C+cognitive+abilities+and+environmental+factors+on+young+children%27s+development+of+absolute+and+relative+pitch+perception&author=Moreno+Sala%2C+Maria+Teresa&issn=&title=The+influence+of+perceptual+shift%2C+cognitive+abilities+and+environmental+factors+on+young+children%27s+development+of+absolute+and+relative+pitch+perception&volume=&issue=&date=2005-01-01&spage=&id=doi:&sid=ProQ_ss&genre=article

Morse, M. (2017). Physical Activity in the Preschool Classroom: An Approach to Enhance Executive Functioning Through the Move for Thought PreK-K Program (Publication Number 10681038) [M.S., Iowa State University]. ProQuest Dissertations & Theses Global. Ann Arbor. http://search.proquest.com.libraryproxy.griffith.edu.au/dissertations-theses/physical-activity-preschool-classroom-approach/docview/2021741454/se-2?accountid=14543 http://hy8fy9jj4b.search.serialssolutions.com/directLink?&atitle=Physical+Activity+in+the+Preschool+Classroom%3A+An+Approach+to+Enhance+Executive+Functioning+Through+the+Move+for+Thought+PreK-K+Program&author=Morse%2C+Morgan&issn=&title=Physical+Activity+in+the+Preschool+Classroom%3A+An+Approach+to+Enhance+Executive+Functioning+Through+the+Move+for+Thought+PreK-K+Program&volume=&issue=&date=2017-01-01&spage=&id=doi:&sid=ProQ_ss&genre=article

Mortimer, J., Krysztofiak, J., Custard, S., & McKune, A. J. (2011). Sport stacking in auditory and visual attention of grade 3 learners [Comparative Study; Journal Article; Randomized Controlled Trial]. Perceptual and Motor Skills, 113(1), 98‐112. https://doi.org/10.2466/05.11.25.PMS.113.4.98-112

Mulvey, K. L., Taunton, S., Pennell, A., & Brian, A. (2018). Head, Toes, Knees, SKIP! Improving Preschool Children's Executive Function Through a Motor Competence Intervention. Journal of Sport & Exercise Psychology, 40(5), 233-239. https://doi.org/10.1123/jsep.2018-0007

Munoz-Parreno, J. A. B.-P., N. Manzano-Sanchez, D. Valero-Valenzuela, A. (2021). The Effect of an Active Breaks Program on Primary School Students' Executive Functions and Emotional Intelligence. Psicothema, 33(3), 466-472. https://doi.org/10.7334/psicothema2020.201

Muñoz-Suazo, D., Díaz-Román, A., Navarro Muñoz, J., Camacho Lazarraga, P., Robles Rodríguez, A., Ibáñez Alcayde, M., Coronilla, M., García Eugenia, G., González Alba, C., & Cano García, R. (2019). Mejora de la atención en niños y niñas con TDAH tras una intervención física deportiva dirigida. / Improving attention in children with ADHD after a directed physical activity intervention. Cuadernos de Psicología del Deporte, 19(3), 37-46. https://search.ebscohost.com/login.aspx?direct=true&db=sph&AN=140996794&site=ehost-live&scope=site

Nan, Y., Liu, L., Geiser, E., Shu, H., Gong, C. C., Dong, Q., Gabrieli, J. D. E., & Desimone, R. (2018). Piano training enhances the neural processing of pitch and improves speech perception in Mandarin-speaking children. Proceedings of the National Academy of Sciences of the United States of America, 115(28), E6630-E6639. https://doi.org/https://dx.doi.org/10.1073/pnas.1808412115

Nejati, V. (2021). Balance-based Attentive Rehabilitation of Attention Networks (BARAN) improves executive functions and ameliorates behavioral symptoms in children with ADHD [Article]. Complementary Therapies in Medicine, 60. https://doi.org/10.1016/j.ctim.2021.102759

Nejati, V., & Derakhshan, Z. (2021). The effect of physical activity with and without cognitive demand on the improvement of executive functions and behavioral symptoms in children with ADHD [Journal: Article in Press]. Expert review of neurotherapeutics. https://doi.org/10.1080/14737175.2021.1912600

Niederer, I. K., S. Zahner, L. Bürgi, F. Ebenegger, V. Hartmann, T. Meyer, U. Schindler, C. Nydegger, A. Marques-Vidal, P. (2009). Influence of a lifestyle intervention in preschool children on physiological and psychological parameters (Ballabeina): study design of a cluster randomized controlled trial [Journal Article; Multicenter Study; Randomized Controlled Trial; Research Support, Non‐U.S. Gov't]. BMC PUBLIC HEALTH, 9, 94. https://doi.org/10.1186/1471-2458-9-94

Nixon, A. M. (2008). An investigation of the relationship between movement breaks and attention (Publication Number 3310892) [Ph.D., Capella University]. ProQuest Dissertations & Theses Global. Ann Arbor. http://search.proquest.com.libraryproxy.griffith.edu.au/dissertations-theses/investigation-relationship-between-movement/docview/304833174/se-2?accountid=14543 http://hy8fy9jj4b.search.serialssolutions.com/directLink?&atitle=An+investigation+of+the+relationship+between+movement+breaks+and+attention&author=Nixon%2C+Anne+Marie&issn=&title=An+investigation+of+the+relationship+between+movement+breaks+and+attention&volume=&issue=&date=2008-01-01&spage=&id=doi:&sid=ProQ_ss&genre=article

O'Brien, C. C. (2015). Physical activity impact on executive function and academic achievement with elementary students https://login.libraryproxy.griffith.edu.au/login?url=https://www.proquest.com/docview/1776620989?accountid=14543&bdid=37598&_bd=%2BCoIaebomKgShF%2FrVgpxbsqd6ZU%3D

O'Donnell, K. (2011). Examining joint attention and its effect on skill acquisition in children with autism using a group drumming therapy approach (Publication Number 3483137) [Ph.D., Texas A&M University - Commerce]. ProQuest Dissertations & Theses Global. Ann Arbor. http://search.proquest.com.libraryproxy.griffith.edu.au/dissertations-theses/examining-joint-attention-effect-on-skill/docview/902512076/se-2?accountid=14543 http://hy8fy9jj4b.search.serialssolutions.com/directLink?&atitle=Examining+joint+attention+and+its+effect+on+skill+acquisition+in+children+with+autism+using+a+group+drumming+therapy+approach&author=O%27Donnell%2C+Kristin&issn=&title=Examining+joint+attention+and+its+effect+on+skill+acquisition+in+children+with+autism+using+a+group+drumming+therapy+approach&volume=&issue=&date=2011-01-01&spage=&id=doi:&sid=ProQ_ss&genre=article

Oppici, L., Rudd, J. R., Buszard, T., & Spittle, S. (2020). Efficacy of a 7-week dance (RCT) PE curriculum with different teaching pedagogies and levels of cognitive challenge to improve working memory capacity and motor competence in 8–10 years old children [Academic Journal]. Psychology of Sport and Exercise, 50. https://doi.org/10.1016/j.psychsport.2020.101675

Orr, A. B. P., Keiver, K., Bertram, C. P., & Clarren, S. (2018). FAST Club: The Impact of a Physical Activity Intervention on Executive Function in Children With Fetal Alcohol Spectrum Disorder. Adapted Physical Activity Quarterly, 35(4), 403-423. https://doi.org/10.1123/apaq.2017-0137

Orsmond, G. I. (1997). Cognitive correlates of early music instruction (Publication Number 9815180) [Ph.D., University of Illinois at Chicago]. ProQuest Dissertations & Theses Global. Ann Arbor. http://search.proquest.com.libraryproxy.griffith.edu.au/dissertations-theses/cognitive-correlates-early-music-instruction/docview/304417064/se-2?accountid=14543 http://hy8fy9jj4b.search.serialssolutions.com/directLink?&atitle=Cognitive+correlates+of+early+music+instruction&author=Orsmond%2C+Gael+Ingrid&issn=&title=Cognitive+correlates+of+early+music+instruction&volume=&issue=&date=1997-01-01&spage=&id=doi:&sid=ProQ_ss&genre=article

Painter, G. (1966). The effect of a rhythmic and sensory motor activity program on perceptual motor spatial abilities of kindergarten children [Article]. Exceptional children, 33(2), 113-116. https://www.embase.com/search/results?subaction=viewrecord&id=L87112538&from=export

Pan, C. Y., Chu, C. H., Tsai, C. L., Lo, S. Y., Cheng, Y. W., & Liu, Y. J. (2016). A racket-sport intervention improves behavioral and cognitive performance in children with attention-deficit/hyperactivity disorder [Journal Article; Randomized Controlled Trial]. Research in Developmental Disabilities, 57, 1‐10. https://doi.org/10.1016/j.ridd.2016.06.009

Pan, C.-Y., Tsai, C.-L., Chu, C.-H., Sung, M.-C., Huang, C.-Y., & Ma, W.-Y. (2019). Effects of physical exercise intervention on motor skills and executive functions in children with ADHD: A pilot study. Journal of Attention Disorders, 23(4), 384-397.

Pan, C.-Y. C., Chia-Hua Tsai, Chia-Liang Sung, Ming-Chih Huang, Chu-Yang Ma, Wei-Ya. (2017). The impacts of physical activity intervention on physical and cognitive outcomes in children with autism spectrum disorder. Autism : the international journal of research and practice, 21(2), 190-202. https://doi.org/https://dx.doi.org/10.1177/1362361316633562

Panczykowski, H., Murphy, L., Adams, K., Bralley, M., & Millner, L. (2021). The Impact of an Interactive Vaulting Equine Program on Executive Function and Group Participation of Children with Disabilities: A Mixed Methods Pilot Study. Alternative & Complementary Therapies, 27(4), 187-195. https://doi.org/10.1089/act.2021.29340.hpa

Park, S. L., J. M. Baik, Y. Kim, K. Yun, H. J. Kwon, H. Jung, Y. K. Kim, B. N. (2015). A Preliminary Study of the Effects of an Arts Education Program on Executive Function, Behavior, and Brain Structure in a Sample of Nonclinical School-Aged Children [Article]. Journal of Child Neurology, 30(13), 1757-1766. https://doi.org/10.1177/0883073815579710

Patrizia, T., Guido, F., Roberto, C., Rosaria, S., & Salvatore, P. (2019). The role of the educator/adult in supporting children of pre-school age in learning difficult tasks: the case of the Playground "Primo Sport 0246". Journal of Physical Education & Sport, 19, 2015-2023. https://search.ebscohost.com/login.aspx?direct=true&db=sph&AN=139461134&site=ehost-live&scope=site

Pedro Ángel, L. R., Beatriz, B. A., Jerónimo, A. V., & Antonio, P. V. (2021). Effects of a 10-week active recess program in school setting on physical fitness, school aptitudes, creativity and cognitive flexibility in elementary school children. A randomised-controlled trial [Journal Article; Randomized Controlled Trial]. Journal of Sports Sciences, 39(11), 1277‐1286. https://doi.org/10.1080/02640414.2020.1864985

Pesce, C., Crova, C., Marchetti, R., Struzzolino, I., Masci, I., Vannozzi, G., & Forte, R. (2013). Searching for cognitively optimal challenge point in physical activity for children with typical and atypical motor development [Article]. Mental Health and Physical Activity, 6(3), 172-180. https://doi.org/10.1016/j.mhpa.2013.07.001

Pesce, C., Lakes, K. D., Stodden, D. F., & Marchetti, R. (2021). Fostering Self-Control Development With a Designed Intervention in Physical Education: a Two-Year Class-Randomized Trial [Journal article]. Child Development, 92(3), 937‐958. https://doi.org/10.1111/cdev.13445

Pesce, C., Masci, I., Marchetti, R., Vazou, S., Saakslahti, A., & Tomporowski, P. D. (2016). Deliberate play and preparation jointly benefit motor and cognitive development: Mediated and moderated effects [Cognitive & Perceptual Development 2820]. FRONTIERS IN PSYCHOLOGY, 7. https://doi.org/http://dx.doi.org/10.3389/fpsyg.2016.00349

Petrenko, N. B. (2016). MASTERING OF MUSICAL RHYTHM BY PRE-SCHOOL AGE CHILDREN WITH SPEECH DISORDERS WITH THE HELP OF DANCE-CORRECTION PROGRAM TRAININGS. PEDAGOGICS PSYCHOLOGY MEDICAL-BIOLOGICAL PROBLEMS OF PHYSICAL TRAINING AND SPORTS, 20(4), 23-28. https://doi.org/10.15561/18189172.2016.0404

Petrenko, N. B., & Filippov, M. M. (2017). Potentials of speech disorders correction in 4-6 yrs children by means of ergo and art therapy. PEDAGOGICS PSYCHOLOGY MEDICAL-BIOLOGICAL PROBLEMS OF PHYSICAL TRAINING AND SPORTS, 21(2), 75-81. https://doi.org/10.15561/18189172.2017.0205

Phung, J. N. (2017). Mixed Martial Arts as a Means to Improve Social Communication and Executive Functioning in Children with Autism Spectrum Disorder. Mixed Martial Arts as a Means to Improve Social Communication & Executive Functioning in Children With Autism Spectrum Disorder, 1-1. http://libraryproxy.griffith.edu.au/login?url=https://search.ebscohost.com/login.aspx?direct=true&db=ccm&AN=131792220&site=ehost-live&scope=site

Pienaar, A. E., Van Rensburg, E., & Smit, A. (2011). EFFECT OF A KINDERKINETICS PROGRAMME ON COMPONENTS OF CHILDREN'S PERCEPTUAL-MOTOR AND COGNITIVE FUNCTIONING. South African Journal for Research in Sport, Physical Education & Recreation, 33(3), 113-128. https://search.ebscohost.com/login.aspx?direct=true&db=sph&AN=67764024&site=ehost-live&scope=site

Polevoy, G. G. (2021). Development of attention stability in children aged 9-10 years with the help of exercises classics [Article]. Indian Journal of Forensic Medicine and Toxicology, 15(3), 3882-3887. https://doi.org/10.37506/IJFMT.V15I3.15900

Polevoy, G. G. (2021). Influence of exercise classics on the development the volume of attention of schoolchildren aged 8-9 [Article]. Indian Journal of Forensic Medicine and Toxicology, 15(1), 1414-1418. https://doi.org/10.37506/ijfmt.v15i1.13611

Portowitz, A., Peppler, K. A., & Downton, M. (2014). In Harmony: A technology-based music education model to enhance musical understanding and general learning skills [Article]. INTERNATIONAL JOURNAL OF MUSIC EDUCATION, 32(2), 242-260. https://doi.org/10.1177/0255761413517056

Priddle, R. E., & Rubin, K. H. (1977). A comparison of two methods for the training of spatial cognition [Cognitive & Perceptual Development 2820]. Merrill-Palmer Quarterly, 23(1), 57-65. https://ovidsp.ovid.com/ovidweb.cgi?T=JS&PAGE=reference&D=psyc2&NEWS=N&AN=1978-07691-001

Ramah, N. (2014). THE EFFECTS OF SQUARE - STEPPING EXERCISES ON COGNITIVE SKILLS FOR KINDERGARTEN AGE CHILDREN. Ovidius University Annals, Series Physical Education & Sport/Science, Movement & Health, 14(1), 126-130. https://search.ebscohost.com/login.aspx?direct=true&db=sph&AN=94810529&site=ehost-live&scope=site

Rauscher, F. H., & Hinton, S. C. (2011). Music instruction and its diverse extra-musical benefits [Curriculum & Programs & Teaching Methods 3530]. MUSIC PERCEPTION, 29(2), 215-226. https://doi.org/http://dx.doi.org/10.1525/mp.2011.29.2.215

Rauscher, F. H., Shaw, G. L., Levine, L. J., Wright, E. L., Dennis, W. R., & Newcomb, R. L. (1997). Music training causes long-term enhancement of preschool children's spatial-temporal reasoning [Article]. Neurological Research, 19(1), 2-8. https://doi.org/10.1080/01616412.1997.11740765

Rauscher, F. H., & Zupan, M. A. (2000). Classroom keyboard instruction improves kindergarten children's spatial-temporal performance: A field experiment [Academic Learning & Achievement 3550]. Early Childhood Research Quarterly, 15(2), 215-228. https://doi.org/http://dx.doi.org/10.1016/S0885-2006%2800%2900050-8

Richard, L. (2015). Cardiorespiratory Exercise Improves White Matter Microstructure and Processing Speed in Childhood Survivors of Brain Tumours (Publication Number 1604817) [M.A., University of Toronto (Canada)]. ProQuest Dissertations & Theses Global. Ann Arbor. http://search.proquest.com.libraryproxy.griffith.edu.au/dissertations-theses/cardiorespiratory-exercise-improves-white-matter/docview/1748045891/se-2?accountid=14543 http://hy8fy9jj4b.search.serialssolutions.com/directLink?&atitle=Cardiorespiratory+Exercise+Improves+White+Matter+Microstructure+and+Processing+Speed+in+Childhood+Survivors+of+Brain+Tumours&author=Richard%2C+Logan&issn=&title=Cardiorespiratory+Exercise+Improves+White+Matter+Microstructure+and+Processing+Speed+in+Childhood+Survivors+of+Brain+Tumours&volume=&issue=&date=2015-01-01&spage=&id=doi:&sid=ProQ_ss&genre=article

Rickard, N. S., Vasquez, J. T., Murphy, F., Gill, A., & Toukhsati, S. R. (2010). Benefits of a classroom based instrumental music program on verbal memory of primary school children: a longitudinal study [Article]. Australian Journal of Music Education(1), 36-47. https://search.ebscohost.com/login.aspx?direct=true&db=eue&AN=57672340&site=ehost-live&scope=site

Rochette, F., Moussard, A., & Bigand, E. (2014). Music lessons improve auditory perceptual and cognitive performance in deaf children [Article]. Frontiers in Human Neuroscience, 8(JULY). https://doi.org/10.3389/fnhum.2014.00488

Roden, I. K., Gunter Bongard, Stephan. (2012). Effects of a school-based instrumental music program on verbal and visual memory in primary school children: A longitudinal study [Curriculum & Programs & Teaching Methods 3530]. FRONTIERS IN PSYCHOLOGY, 3. https://doi.org/http://dx.doi.org/10.3389/fpsyg.2012.00572

Rodriguez-Negro, J., Pesola, J. A., & Yanci, J. (2020). Effects and retention of different physical exercise programs on children's cognitive and motor development. JOURNAL OF EDUCATIONAL RESEARCH, 113(6), 431-437. https://doi.org/10.1080/00220671.2020.1854159

Roh, H. T., Cho, S. Y., & So, W. Y. (2018). Taekwondo Training Improves Mood and Sociability in Children from Multicultural Families in South Korea: a Randomized Controlled Pilot Study [Journal Article; Randomized Controlled Trial; Research Support, Non‐U.S. Gov't]. International Journal of Environmental Research and Public Health, 15(4). https://doi.org/10.3390/ijerph15040757

Ros-Silla, E. V.-M., S. Jaikel-Arce, D. Berlai, S. Giglio, R. Payro-Escobar, A. Romero-Naranjo, F. J. (2019). ATTENTION IN CONSERVATOIRE STUDENTS USING BODY PERCUSSION FOLLOWING THE BAPNE METHOD https://www.europeanproceedings.com/files/data/article/85/5295/article_85_5295_pdf_100.pdf

Rudd, J., Buszard, T., Spittle, S., O'Callaghan, L., & Oppici, L. (2021). Comparing the efficacy (RCT) of learning a dance choreography and practicing creative dance on improving executive functions and motor competence in 6–7 years old children. Psychology of Sport & Exercise, 53, N.PAG-N.PAG. https://doi.org/10.1016/j.psychsport.2020.101846

Sa, V. (2020). The Effect of Music Attention Control Training (MACT) for Pre-Adolescents with Autism Spectrum Disorder (Publication Number 27834225) [M.M., University of the Pacific]. ProQuest Dissertations & Theses Global. Ann Arbor. http://search.proquest.com.libraryproxy.griffith.edu.au/dissertations-theses/effect-music-attention-control-training-mact-pre/docview/2395527424/se-2?accountid=14543 http://hy8fy9jj4b.search.serialssolutions.com/directLink?&atitle=The+Effect+of+Music+Attention+Control+Training+%28MACT%29+for+Pre-Adolescents+with+Autism+Spectrum+Disorder&author=Sa%2C+Vienna&issn=&title=The+Effect+of+Music+Attention+Control+Training+%28MACT%29+for+Pre-Adolescents+with+Autism+Spectrum+Disorder&volume=&issue=&date=2020-01-01&spage=&id=doi:&sid=ProQ_ss&genre=article

Sabel, M. S., A. Broeren, J. Arvidsson, D. Saury, J. M. Gillenstrand, J. Emanuelson, I. Blomgren, K. Lannering, B. (2017). Effects of physically active video gaming on cognition and activities of daily living in childhood brain tumor survivors: a randomized pilot study [Article]. Neuro-oncology practice, 4(2), 98‐110. https://doi.org/10.1093/nop/npw020

Sachs, M., Kaplan, J., Der Sarkissian, A., & Habibi, A. (2017). Increased engagement of the cognitive control network associated with music training in children during an fMRI Stroop task. PLoS ONE Vol 12(10), 2017, ArtID e0187254, 12(10). https://libraryproxy.griffith.edu.au/login?url=https://ovidsp.ovid.com/ovidweb.cgi?T=JS&CSC=Y&NEWS=N&PAGE=fulltext&D=psyc16&AN=2018-17234-001 https://griffithuni.on.worldcat.org/atoztitles/link?&rft_val_fmt=info:ofi/fmt:kev:mtx:journal&rfr_id=info:sid/Ovid:psyc16&rft.genre=article&rft_id=info:doi/10.1371%2Fjournal.pone.0187254&rft_id=info:pmid/&rft.issn=1932-6203&rft.volume=12&rft.issue=10&rft.spage=e0187254&rft.pages=&rft.date=2017&rft.jtitle=PLoS+ONE&rft.atitle=Increased+engagement+of+the+cognitive+control+network+associated+with+music+training+in+children+during+an+fMRI+Stroop+task.&rft.aulast=Sachs

Salici, O., & Söyleyici, Z. S. (2020). Investigation of the Effects of Six-Weeks of Regular Table Tennis Education on Attention Levels of Primary School Children. African Educational Research Journal, 8(3), 449-452. https://search.ebscohost.com/login.aspx?direct=true&db=eric&AN=EJ1263354&site=ehost-live&scope=site

Sanchez-Lopez, M., Cavero-Redondo, I., Alvarez-Bueno, C., Ruiz-Hermosa, A., Pozuelo-Carrascosa, D. P., Diez-Fernandez, A., el Campo, D. G.-D., Pardo-Guijarro, M. J., & Martinez-Vizcaino, V. (2019). Impact of a multicomponent physical activity intervention on cognitive performance: The MOVI-KIDS study [Health Psychology & Medicine 3360]. Scandinavian Journal of Medicine & Science in Sports, 29(5), 766-775. https://doi.org/http://dx.doi.org/10.1111/sms.13383

Santner, A. K., Martin Federolf, Peter. (2018). Partly randomised, controlled study in children aged 6–10 years to investigate motor and cognitive effects of a 9-week coordination training intervention with concurrent mental tasks. BMJ Open, 8(5). https://doi.org/http://dx.doi.org/10.1136/bmjopen-2017-021026

Sassi, J. M. (2018). The Impact of a Motor Skill Intervention on Executive Function in Preschoolers from Low Socioeconomic Backgrounds https://login.libraryproxy.griffith.edu.au/login?url=https://www.proquest.com/docview/2779136787?accountid=14543&bdid=37598&_bd=rzjiF84bDrXksfIZcPShbPMd08c%3D

Schlaug, G. N., A. Overy, K. Winner, E. (2005). Effects of music training on the child's brain and cognitive development. In G. K. S. L. L. M. M. Avanzini (Ed.), (Vol. 1060, pp. 219-230). https://doi.org/10.1196/annals.1360.015

Schmidt, M., Benzing, V., Wallman-Jones, A., Mavilidi, M. F., Lubans, D. R., & Paas, F. (2019). Embodied learning in the classroom: Effects on primary school children's attention and foreign language vocabulary learning. Psychology of Sport and Exercise, 43, 45-54. https://doi.org/10.1016/j.psychsport.2018.12.017

Schmidt, M., Mavilidi, M. F., Singh, A., & Englert, C. (2020). Combining physical and cognitive training to improve kindergarten children's executive functions: A cluster randomized controlled trial [Article]. Contemporary Educational Psychology, 63, N.PAG-N.PAG. https://doi.org/10.1016/j.cedpsych.2020.101908

Schmidt, M. B., Valentin Kamer, Mario. (2016). Classroom-based physical activity breaks and children's attention: Cognitive engagement works! [Curriculum & Programs & Teaching Methods 3530]. FRONTIERS IN PSYCHOLOGY, 7. https://doi.org/http://dx.doi.org/10.3389/fpsyg.2016.01474

Schmidt, M. J., Katja Egger, Fabienne Roebers, Claudia M. Conzelmann, Achim. (2015). Cognitively Engaging Chronic Physical Activity, But Not Aerobic Exercise, Affects Executive Functions in Primary School Children: A Group-Randomized Controlled Trial. Journal of Sport & Exercise Psychology, 37(6), 575-591. https://doi.org/https://dx.doi.org/10.1123/jsep.2015-0069

Shaffer, R. J., Jacokes, L. E., Cassily, J. F., Greenspan, S. I., Tuchman, R. F., & Stemmer Jr, P. J. (2001). Effect of interactive metronome training on children with ADHD [Article]. The American journal of occupational therapy. : official publication of the American Occupational Therapy Association, 55(2), 155-162. https://www.embase.com/search/results?subaction=viewrecord&id=L33561495&from=export

Shamshiri, S. S., M. Hemayat Talab, R. Riahi, F. (2018). Comparison of three methods of intervention pharmacotherapy, cognitive-motion rehabilitation and the combination on components of attention of DD children [Article]. Minerva Psichiatrica, 59(1), 29-38. https://doi.org/10.23736/S0391-1772.17.01947-1

Shema-Shiratzky, S., Brozgol, M., Cornejo-Thumm, P., Geva-Dayan, K., Rotstein, M., Leitner, Y., Hausdorff, J. M., & Mirelman, A. (2019). Virtual reality training to enhance behavior and cognitive function among children with attention-deficit/hyperactivity disorder: brief report. Developmental Neurorehabilitation, 22(6), 431-436. https://doi.org/https://dx.doi.org/10.1080/17518423.2018.1476602

Shen, Y., Lin, Y. S., Liu, S. H., Fang, L. L., & Liu, G. (2019). Sustained Effect of Music Training on the Enhancement of Executive Function in Preschool Children. FRONTIERS IN PSYCHOLOGY, 10, Article 1910. https://doi.org/10.3389/fpsyg.2019.01910

Shen, Y., Zhao, Q., Huang, Y., Liu, G., & Fang, L. L. (2020). Promotion of Street-Dance Training on the Executive Function in Preschool Children. FRONTIERS IN PSYCHOLOGY, 11. https://doi.org/10.3389/fpsyg.2020.585598

Sjowall, D., Hertz, M., & Klingberg, T. (2017). No long-term effect of physical activity intervention on working memory or arithmetic in preadolescents [Developmental Psychology 2800]. FRONTIERS IN PSYCHOLOGY, 8. https://doi.org/http://dx.doi.org/10.3389/fpsyg.2017.01342

Skeja, E. (2014). The Impact of Cognitive Intervention Program and Music Therapy in Learning Disabilities https://www.sciencedirect.com/science/article/pii/S187704281406563X?via%3Dihub

Sportsman, E. L. (2011). Development of musicianship and executive functioning among children participating in a music program (Publication Number 3469309) [Ph.D., Michigan State University]. ProQuest Dissertations & Theses Global. Ann Arbor. http://search.proquest.com.libraryproxy.griffith.edu.au/dissertations-theses/development-musicianship-executive-functioning/docview/892713197/se-2?accountid=14543 http://hy8fy9jj4b.search.serialssolutions.com/directLink?&atitle=Development+of+musicianship+and+executive+functioning+among+children+participating+in+a+music+program&author=Sportsman%2C+Emily+Lauren&issn=&title=Development+of+musicianship+and+executive+functioning+among+children+participating+in+a+music+program&volume=&issue=&date=2011-01-01&spage=&id=doi:&sid=ProQ_ss&genre=article

Sullivan, C. (2017). The Effects of a Gross Motor Development Program on Executive Functions in Children With Intellectual Disabilities, Autism Spectrum Disorders, Attention Deficit-Hyperactivity Disorders, and Learning Disabilities https://login.libraryproxy.griffith.edu.au/login?url=https://www.proquest.com/docview/1914683789?accountid=14543&bdid=37598&_bd=xLWiYUMWokP0Nf54AqHpYA6gsYk%3D

Taetle, L. D. (1999). The effect of active and passive music instruction on the spatial ability of kindergarten children (Publication Number 9927519) [Ph.D., The University of Arizona]. ProQuest Dissertations & Theses Global. Ann Arbor. http://search.proquest.com.libraryproxy.griffith.edu.au/dissertations-theses/effect-active-passive-music-instruction-on/docview/304494974/se-2?accountid=14543 http://hy8fy9jj4b.search.serialssolutions.com/directLink?&atitle=The+effect+of+active+and+passive+music+instruction+on+the+spatial+ability+of+kindergarten+children&author=Taetle%2C+Laurie+Daniels&issn=&title=The+effect+of+active+and+passive+music+instruction+on+the+spatial+ability+of+kindergarten+children&volume=&issue=&date=1999-01-01&spage=&id=doi:&sid=ProQ_ss&genre=article

Tanir, A., & Erkut, O. (2018). Effect of Rhythmic Basketball Lessons on Visual Attention Ability and Lay-Up Skill in School Children Aged 9-10. Universal Journal of Educational Research, 6(9), 1857-1862. https://search.ebscohost.com/login.aspx?direct=true&db=eric&AN=EJ1189940&site=ehost-live&scope=site

Tarp, J., Domazet, S. L., Froberg, K., Hillman, C. H., Andersen, L. B., & Bugge, A. (2016). Effectiveness of a School-Based Physical Activity Intervention on Cognitive Performance in Danish Adolescents: lCoMotion-Learning, Cognition and Motion - A Cluster Randomized Controlled Trial [Clinical Trial; Journal Article; Multicenter Study; Randomized Controlled Trial]. PLoS ONE, 11(6), e0158087. https://doi.org/10.1371/journal.pone.0158087

Telles, S., Singh, N., Bhardwaj, A. K., Kumar, A., & Balkrishna, A. (2013). Effect of yoga or physical exercise on physical, cognitive and emotional measures in children: a randomized controlled trial [Journal: Article]. Child and adolescent psychiatry and mental health, 7(1). https://doi.org/10.1186/1753-2000-7-37

Torro-Biosca, R., Aparici-Minguez, F., Arnau-Molla, A. F., Ulate-Orozco, R. M., Cabrera-Quiros, D. A., & Romero-Naranjo, F. J. (2018, Jun 20-22

2018). PILOT STUDY INTO THE EXECUTIVE FUNCTIONS OF CHILDREN AGED 8-9 BAPNE METHOD.European Proceedings of Social and Behavioural Sciences [Viii international conference on intercultural education and international conference on transcultural health: The value of education and health for a global, transcultural world (eduhem 2018)]. Almeria, SPAIN.

Tottori, N., Morita, N., Ueta, K., & Fujita, S. (2019). Effects of High Intensity Interval Training on Executive Function in Children Aged 8-12 Years. International Journal of Environmental Research and Public Health, 16(21), Article 4127. https://doi.org/10.3390/ijerph16214127

Trinchitella, S. E. (2016). Immediate Effects of Exercise on Attention in Preschool Children (Publication Number 10138293) [Psy.D., Hofstra University]. ProQuest Dissertations & Theses Global. Ann Arbor. http://search.proquest.com.libraryproxy.griffith.edu.au/dissertations-theses/immediate-effects-exercise-on-attention-preschool/docview/1820079780/se-2?accountid=14543 http://hy8fy9jj4b.search.serialssolutions.com/directLink?&atitle=Immediate+Effects+of+Exercise+on+Attention+in+Preschool+Children&author=Trinchitella%2C+Sarah+E.&issn=&title=Immediate+Effects+of+Exercise+on+Attention+in+Preschool+Children&volume=&issue=&date=2016-01-01&spage=&id=doi:&sid=ProQ_ss&genre=article

Tsai, C.-L. (2009). The effectiveness of exercise intervention on inhibitory control in children with developmental coordination disorder: Using a visuospatial attention paradigm as a model [Article]. Research in Developmental Disabilities, 30(6), 1268-1280. https://doi.org/10.1016/j.ridd.2009.05.001

Tsai, C. L., Chang, Y. K., Chen, F. C., Hung, T. M., Pan, C. Y., & Wang, C. H. (2014). Effects of cardiorespiratory fitness enhancement on deficits in visuospatial working memory in children with developmental coordination disorder: a cognitive electrophysiological study [Journal Article; Randomized Controlled Trial; Research Support, Non‐U.S. Gov't]. ARCHIVES OF CLINICAL NEUROPSYCHOLOGY, 29(2), 173‐185. https://doi.org/10.1093/arclin/act081

Tse, A. C. Y., Anderson, D. I., Liu, V. H. L., & Tsui, S. S. L. (2021). Improving Executive Function of Children with Autism Spectrum Disorder through Cycling Skill Acquisition [Journal: Article]. Medicine and Science in Sports and Exercise, 53(7), 1417‐1424. https://doi.org/10.1249/MSS.0000000000002609

Tse, C. Y. A., Lee, H. P., Chan, K. S. K., Edgar, B. V., Wilkinson-Smith, A., & Lai, W. H. E. (2019). Examining the impact of physical activity on sleep quality and executive functions in children with autism spectrum disorder: a randomized controlled trial [Journal: Article in Press]. Autism. https://doi.org/10.1177/1362361318823910

Tseng, W. Y. R., G. Chen, C. H. Clemente, F. M. Bezerra, P. Crowley-McHattan, Z. J. Chen, Y. S. (2021). Effects of 8-Week FIFA 11+ for Kids Intervention on Physical Fitness and Attention in Elementary School Children [Article]. Journal of Physical Activity & Health, 18(6), 686-693. https://doi.org/10.1123/jpah.2020-0823

Ustun, F., & Tasgin, E. (2020). The Effect of Recreative Purpose Modern and Traditional Archery Education on Attention Parameters in Adolescents. Journal of Education and Learning, 9(1), 244-250. https://search.ebscohost.com/login.aspx?direct=true&db=eric&AN=EJ1244942&site=ehost-live&scope=site

Valentini, N. C., Pierosan, L., Rudisill, M. E., & Hastie, P. A. (2017). Mastery and exercise play interventions: motor skill development and verbal recall of children with and without disabilities [Article]. Physical Education & Sport Pedagogy, 22(4), 349-363. https://doi.org/10.1080/17408989.2016.1241223

Valentinovna, P. Y., Ivanovna, D. G., Leonidovna, L. V., & Sergeevich, F. A. (2019). The use of karate for the correction of mental processes in children of primary school age with hearing impairment. In V. V. S. S. G. Erlikh (Ed.), (Vol. 17, pp. 195-198). <Go to ISI>://WOS:000625435700050

van Cappellen - van Maldegem, S. J. M., van Abswoude, F., Krajenbrink, H., & Steenbergen, B. (2018). Motor learning in children with developmental coordination disorder: The role of focus of attention and working memory [Developmental Disorders & Autism 3250]. Human Movement Science, 62, 211-220. https://doi.org/http://dx.doi.org/10.1016/j.humov.2018.11.001

van den Berg, V., Saliasi, E., e Groot, R. H., Chinapaw, M. J., & Singh, A. S. (2019). Improving cognitive performance of 9-12 years old children: just dance? A randomized controlled trial. Frontiers in psychology vol 10 2019, artid 174, 10. https://www.cochranelibrary.com/central/doi/10.1002/central/CN-01911579/full

van der Niet, A. G., Smith, J., Oosterlaan, J., Scherder, E. J., Hartman, E., & Visscher, C. (2016). Effects of a Cognitively Demanding Aerobic Intervention During Recess on Children's Physical Fitness and Executive Functioning [Article]. Pediatric Exercise Science, 28(1), 64-70. https://doi.org/10.1123/pes.2015-0084

Van Stryp, O. (2021). The Effect of Active Brain-Breaks on In-School Physical Activity, Fundamental Movement Skills and Executive Functioning in Grade One Children https://login.libraryproxy.griffith.edu.au/login?url=https://www.proquest.com/docview/2607496354?accountid=14543&bdid=37598&_bd=KTKv5FnVtCBou8DVDXHTLp%2BmcoY%3D

Vazou, S., Klesel, B., Lakes, K. D., & Smiley, A. (2020). Rhythmic Physical Activity Intervention: Exploring Feasibility and Effectiveness in Improving Motor and Executive Function Skills in Children. FRONTIERS IN PSYCHOLOGY, 11, Article 556249. https://doi.org/10.3389/fpsyg.2020.556249

Vazou, S., Long, K., Lakes, K. D., & Whalen, N. L. (2021). "Walkabouts" Integrated Physical Activities from Preschool to Second Grade: Feasibility and Effect on Classroom Engagement. Child & Youth Care Forum, 50(1), 39-55. https://doi.org/10.1007/s10566-020-09563-4

Veldman, S. L. C. J., R. A. Stanley, R. M. Cliff, D. P. Vella, S. A. Howard, S. J. Parrish, A. M. Okely, A. D. (2020). Promoting Physical Activity and Executive Functions Among Children: a Cluster Randomized Controlled Trial of an After-School Program in Australia [Journal: Article in Press]. Journal of Physical Activity & Health, 1‐7. https://doi.org/10.1123/jpah.2019-0381

Verret, C. (2010). Condition physique, performance motrice, comportements et fonctions cognitives chez les enfants ayant un trouble du déficit de l'attention avec hyperactivité (Publication Number NR70437) [Ph.D., Universite de Montreal (Canada)]. ProQuest Dissertations & Theses Global. Ann Arbor. http://search.proquest.com.libraryproxy.griffith.edu.au/dissertations-theses/condition-physique-performance-motrice/docview/853730496/se-2?accountid=14543 http://hy8fy9jj4b.search.serialssolutions.com/directLink?&atitle=Condition+physique%2C+performance+motrice%2C+comportements+et+fonctions+cognitives+chez+les+enfants+ayant+un+trouble+du+d%C3%A9ficit+de+l%27attention+avec+hyperactivit%C3%A9&author=Verret%2C+Claudia&issn=&title=Condition+physique%2C+performance+motrice%2C+comportements+et+fonctions+cognitives+chez+les+enfants+ayant+un+trouble+du+d%C3%A9ficit+de+l%27attention+avec+hyperactivit%C3%A9&volume=&issue=&date=2010-01-01&spage=&id=doi:&sid=ProQ_ss&genre=article

von, R.-G., Irene Elisabeth. (1998). "Creative dance": Potentiality for enhancing psychomotor, cognitive, and social-affective functioning in seniors and young children (Publication Number NQ24361) [Ph.D., Simon Fraser University (Canada)]. ProQuest Dissertations & Theses Global. Ann Arbor. http://search.proquest.com.libraryproxy.griffith.edu.au/dissertations-theses/creative-dance-potentiality-enhancing-psychomotor/docview/304489486/se-2?accountid=14543 http://hy8fy9jj4b.search.serialssolutions.com/directLink?&atitle=%22Creative+dance%22%3A+Potentiality+for+enhancing+psychomotor%2C+cognitive%2C+and+social-affective+functioning+in+seniors+and+young+children&author=von+Rossberg-Gempton%2C+Irene+Elisabeth&issn=&title=%22Creative+dance%22%3A+Potentiality+for+enhancing+psychomotor%2C+cognitive%2C+and+social-affective+functioning+in+seniors+and+young+children&volume=&issue=&date=1998-01-01&spage=&id=doi:&sid=ProQ_ss&genre=article

Wallace, J. I. (1984). EFFECTS OF RELAXATION AND PHYSICAL CONDITIONING PROGRAMS UPON IMPULSIVITY IN THIRD- AND FOURTH-GRADE MALES (Publication Number 0555450) [Ph.D., University of Southern California]. ProQuest Dissertations & Theses Global. Ann Arbor. http://search.proquest.com.libraryproxy.griffith.edu.au/dissertations-theses/effects-relaxation-physical-conditioning-programs/docview/303327631/se-2?accountid=14543 http://hy8fy9jj4b.search.serialssolutions.com/directLink?&atitle=EFFECTS+OF+RELAXATION+AND+PHYSICAL+CONDITIONING+PROGRAMS+UPON+IMPULSIVITY+IN+THIRD-+AND+FOURTH-GRADE+MALES&author=WALLACE%2C+JAMES+IRWIN&issn=&title=EFFECTS+OF+RELAXATION+AND+PHYSICAL+CONDITIONING+PROGRAMS+UPON+IMPULSIVITY+IN+THIRD-+AND+FOURTH-GRADE+MALES&volume=&issue=&date=1984-01-01&spage=&id=doi:&sid=ProQ_ss&genre=article

Wang, J. G., Cai, K. L., Liu, Z. M., Herold, F., Zou, L. Y., Zhu, L. N., Xiong, X., & Chen, A. G. (2020). Effects of Mini-Basketball Training Program on Executive Functions and Core Symptoms among Preschool Children with Autism Spectrum Disorders. Brain Sciences, 10(5), Article 263. https://doi.org/10.3390/brainsci10050263

Watson-Grace, A., & Provident, I. (2020). Improving Selective Attention for All Students with Coordinative Bal-A-Vis-X Movement Breaks: A Pilot Study. Journal of Occupational Therapy, Schools & Early Intervention, 13(4), 420-442. https://search.ebscohost.com/login.aspx?direct=true&db=eric&AN=EJ1279103&site=ehost-live&scope=site http://dx.doi.org/10.1080/19411243.2020.1769000

Weerdmeester, J., Cima, M., Granic, I., Hashemian, Y., & Gotsis, M. (2016). A Feasibility Study on the Effectiveness of a Full-Body Videogame Intervention for Decreasing Attention Deficit Hyperactivity Disorder Symptoms [Journal Article; Randomized Controlled Trial]. Games for health journal, 5(4), 258‐269. https://doi.org/10.1089/g4h.2015.0103

Westendorp, M., Houwen, S., Hartman, E., Mombarg, R., Smith, J., & Visscher, C. (2014). Effect of a ball skill intervention on children's ball skills and cognitive functions [Journal Article; Randomized Controlled Trial]. Medicine and Science in Sports and Exercise, 46(2), 414‐422. https://doi.org/10.1249/MSS.0b013e3182a532b3

Wexler, B. E. (2019). Computer-presented and physical brain-training exercises for school children: Improving executive functions and learning [Educational Psychology 3500]. Early childhood development: Concepts, methodologies, tools, and applications., 1105-1123. https://doi.org/http://dx.doi.org/10.4018/978-1-5225-7507-8.ch055

Williams, K. E., & Berthelsen, D. (2019). Implementation of a rhythm and movement intervention to support self-regulation skills of preschool-aged children in disadvantaged communities. PSYCHOLOGY OF MUSIC, 47(6), 800-820, Article 0305735619861433. https://doi.org/10.1177/0305735619861433

Wilson, A. N., Olds, T., Lushington, K., Petkov, J., & Dollman, J. (2016). The impact of 10-minute activity breaks outside the classroom on male students' on-task behaviour and sustained attention: a randomised crossover design [Journal Article; Randomized Controlled Trial]. Acta paediatrica, 105(4), e181‐188. https://doi.org/10.1111/apa.13323

Wooledge, D. E. (1988). The effects of gross motor activities on asymmetrical motor performance and integrated style of information processing (Publication Number 8904473) [Ed.D., University of Missouri - Columbia]. ProQuest Dissertations & Theses Global. Ann Arbor. http://search.proquest.com.libraryproxy.griffith.edu.au/dissertations-theses/effects-gross-motor-activities-on-asymmetrical/docview/303569045/se-2?accountid=14543 http://hy8fy9jj4b.search.serialssolutions.com/directLink?&atitle=The+effects+of+gross+motor+activities+on+asymmetrical+motor+performance+and+integrated+style+of+information+processing&author=Wooledge%2C+Doris+Edith&issn=&title=The+effects+of+gross+motor+activities+on+asymmetrical+motor+performance+and+integrated+style+of+information+processing&volume=&issue=&date=1988-01-01&spage=&id=doi:&sid=ProQ_ss&genre=article

Xiang, M.-Q., Liao, J.-W., Huang, J.-H., Deng, H.-L., Wang, D., Xu, Z., & Hu, M. (2019). Effect of a combined exercise and dietary intervention on self-control in obese adolescents [Promotion & Maintenance of Health & Wellness 3365]. FRONTIERS IN PSYCHOLOGY, 10. https://doi.org/http://dx.doi.org/10.3389/fpsyg.2019.01385

Xiaoyan, J., Traverso, L., & Xiaosong, G. (2021). Examining the Effectiveness of Group Games in Enhancing Inhibitory Control in Preschoolers [Article]. Early Education & Development, 32(5), 741-763. https://doi.org/10.1080/10409289.2020.1802972

Xiong, S. Y., Li, X. X., & Tao, K. (2017). Effects of Structured Physical Activity Program on Chinese Young Children's Executive Functions and Perceived Physical Competence in a Day Care Center. BioMed Research International, 2017, Article 5635070. https://doi.org/10.1155/2017/5635070

Xiong, S. Y., Zhang, P., & Gao, Z. (2019). Effects of Exergaming on Preschoolers' Executive Functions and Perceived Competence: A Pilot Randomized Trial. Journal of Clinical Medicine, 8(4), Article 469. https://doi.org/10.3390/jcm8040469

Zach, S., Inglis, V., Fox, O., Berger, I., & Stahl, A. (2015). The effect of physical activity on spatial perception and attention in early childhood [Article]. Cognitive Development, 36, 31-39. https://doi.org/10.1016/j.cogdev.2015.08.003

Zafranas, N. (2003). The effects of piano-keyboard instruction on cognitive abilities of female and male kindergarten children (Publication Number U162764) [Ph.D., Brunel University (United Kingdom)]. ProQuest Dissertations & Theses Global. Ann Arbor. http://search.proquest.com.libraryproxy.griffith.edu.au/dissertations-theses/effects-piano-keyboard-instruction-on-cognitive/docview/301622274/se-2?accountid=14543 http://hy8fy9jj4b.search.serialssolutions.com/directLink?&atitle=The+effects+of+piano-keyboard+instruction+on+cognitive+abilities+of+female+and+male+kindergarten+children&author=Zafranas%2C+Nikolaos&issn=&title=The+effects+of+piano-keyboard+instruction+on+cognitive+abilities+of+female+and+male+kindergarten+children&volume=&issue=&date=2003-01-01&spage=&id=doi:&sid=ProQ_ss&genre=article

Zafranas, N. (2004). Piano keyboard training and the spatial-temporal development of young children attending kindergarten classes in Greece [Article]. Early Child Development & Care, 174(2), 199-211. https://doi.org/10.1080/0300443032000153534

Ziereis, S., & Jansen, P. (2015). Effects of physical activity on executive function and motor performance in children with ADHD [Journal Article; Randomized Controlled Trial]. Research in Developmental Disabilities, 38, 181‐191. https://doi.org/10.1016/j.ridd.2014.12.005

**C.2 Reference list of included parent studies in EGM – Updated Search (April 2025)**

Akil, M., Tokay, B., & Gungor, M. G. (2024). Cognitive health outcomes of fundamental motor skill applications in children through cooperative learning method. *BMC Psychology*, *12*(1), 522. <https://doi.org/10.1186/s40359-024-02006-y>

Alesi, M., Bianco, A., Luppina, G., Palma, A., & Pepi, A. (2016). Improving children's coordinative skills and executive functions: The effects of a football exercise program. *Perceptual and Motor Skills*, *122*(1), 27-46. <https://doi.org/10.1177/0031512515627527>

Alonso-Martinez, A. M., Legarra-Gorgonon, G., Garcia-Alonso, Y., Ramirez-Velez, R., Alonso-Martinez, L., Erice-Echegaray, B., & Izquierdo, M. (2023). Gamified family-based health exercise intervention to improve adherence to 24-h movement behaviors recommendations in children: "3, 2, 1 Move on Study". *Trials*, *24*(1), 531. <https://doi.org/10.1186/s13063-023-07494-8>

Amini, B., Hosseini, S. A., Pishyareh, E., Bakhshi, E., & Haghgoo, H. A. (2024). Designing an exercise protocol to improve impulsivity control in children with attention deficit hyperactivity disorder: A pilot study. *Journal of Rehabilitation*, *25*(Special Issue), 702-725. <https://doi.org/10.32598/RJ.25.specialissue.3833.1>

Anguera, J. A., Rowe, M. A., Volponi, J. J., Elkurdi, M., Jurigova, B., Simon, A. J., Anguera-Singla, R., Gallen, C. L., Gazzaley, A., & Marco, E. J. (2023). Enhancing attention in children using an integrated cognitive-physical videogame: A pilot study. *NPJ Digital Medicine*, *6*(1), 65. <https://doi.org/10.1038/s41746-023-00812-z>

Aoyama, S. (2024). Effects of soccer instruction on the executive functions and agility of children in early childhood. *PLoS ONE*, *19*(10), e0312265. <https://doi.org/10.1371/journal.pone.0312265>

Barnard, R. (2022). *The Influence of Short-Term Music Programming on Learning Executive Functioning Skills in Preschool Children* [Capella University]. <https://www.proquest.com/docview/2747912757?pq-origsite=gscholar&fromopenview=true&sourcetype=Dissertations%20&%20Theses>

Biino, V., Tinagli, V., Borioni, F., & Pesce, C. (2021). Cognitively enriched physical activity may foster motor competence and executive function as early as preschool age: A pilot trial. *Physical Education and Sport Pedagogy*, *28*(4), 425-443. <https://doi.org/10.1080/17408989.2021.1990249>

Boat, R., Cooper, S. B., Carlevaro, F., Magno, F., Bardaglio, G., Musella, G., & Magistro, D. (2022). 16 weeks of physically active mathematics and english language lessons improves cognitive function and gross motor skills in children aged 8-9 years. *Int J Environ Res Public Health*, *19*(24). <https://doi.org/10.3390/ijerph192416751>

Booth, J. N., Mitchell, I. A., Tomporowski, P. D., McCullick, B. A., Boyle, J. M. E., & Reilly, J. J. (2023). Evaluation of a pilot physical activity intervention for children with ADHD symptoms and reading difficulties. *Journal of Research in Special Educational Needs*, *24*(2), 254-268. <https://doi.org/10.1111/1471-3802.12628>

Borioni, F., Biino, V., Tinagli, V., & Pesce, C. (2022). Effects of baby swimming on motor and cognitive development: A pilot trial. *Percept Mot Skills*, *129*(4), 977-1000. <https://doi.org/10.1177/00315125221090203>

Bortz, G., Ilari, B., Di Giuseppe Germano, N., Jackowski, A. P., Cogo-Moreira, H., Lúcio, P. S. . (2024). Music education reduces emotional symptoms in children: A quasi-experimental study in the Guri Program in Brazil. *medRxiv*, *2024*(11). <https://doi.org/10.1101/2024.11.12.24317159>

Bugos, J. A., DeMarie, D., Stokes, C., & L, P. P. (2022). Multimodal music training enhances executive functions in children: Results of a randomized controlled trial. *Ann N Y Acad Sci*, *1516*(1), 95-105. <https://doi.org/10.1111/nyas.14857>

Buker, N., Karagoz, E., Sengul, Y. S., Guney, S. A., & Ozbek, A. (2024). Neurocognitive training enhances the outcomes of children with attention deficit hyperactivity disorder: A preliminary study. *Child Care Health Dev*, *50*(3), e13268. <https://doi.org/10.1111/cch.13268>

Capio, C. M., Mak, T. C. T., Tse, M. A., & Masters, R. S. W. (2018). Fundamental movement skills and balance of children with Down syndrome. *J Intellect Disabil Res*, *62*(3), 225-236. <https://doi.org/10.1111/jir.12458>

Capio, C. M., Mendoza, N. B., Jones, R. A., Masters, R. S. W., & Lee, K. (2024). The contributions of motor skill proficiency to cognitive and social development in early childhood. *Sci Rep*, *14*(1), 27956. <https://doi.org/10.1038/s41598-024-79538-1>

Casella, A., Ventura, E., & Di Russo, F. (2022). The Influence of a Specific Cognitive-Motor Training Protocol on Planning Abilities and Visual Search in Young Soccer Players. *Brain Sci*, *12*(12). <https://doi.org/10.3390/brainsci12121624>

Chang, S. H., Shie, J. J., & Yu, N. Y. (2022). Enhancing Executive Functions and Handwriting with a Concentrative Coordination Exercise in Children with ADHD: A Randomized Clinical Trial. *Percept Mot Skills*, *129*(4), 1014-1035. <https://doi.org/10.1177/00315125221098324>

Chen, H., Liang, Q., Wang, B., Liu, H., Dong, G., & Li, K. (2024). Sports game intervention aids executive function enhancement in children with autism - An fNIRS study. *Neurosci Lett*, *822*, 137647. <https://doi.org/10.1016/j.neulet.2024.137647>

Chou, C. C., Wang, C. H., McCullick, B., & Hsueh, M. C. (2023). Effects of Coordinative Exercise on Sustained Attention and Perceptual Discrimination in Elementary School Physical Education. *Res Q Exerc Sport*, *94*(4), 948-958. <https://doi.org/10.1080/02701367.2022.2085863>

Christ, T., Bostrom, K. J., Ohrmann, P., Britz, H., Wagner, H., & Bohn, C. (2024). The effects of a four-month skateboarding intervention on motor, cognitive, and symptom levels in children with ADHD. *Front Pediatr*, *12*, 1452851. <https://doi.org/10.3389/fped.2024.1452851>

Chung, J. O. K., Li, W. H. C., Leung, Y. D., Cheung, T. C. K., Chiu, S. Y., Pong, M. S. Y., & Chan, G. C. F. (2025). The feasibility, acceptability, and potential efficacy of a musical training program in promoting neuroplasticity among survivors of pediatric brain tumors: A cohort study. *Eur J Oncol Nurs*, *76*, 102851. <https://doi.org/10.1016/j.ejon.2025.102851>

Coates, C. (2021). *Assessing the Impact of a Music Therapy Program on Attention in Children with Autism using Behavioral and Neurophysiological Measures* Colorado State University]. <https://www.proquest.com/docview/2572989803?pq-origsite=gscholar&fromopenview=true&sourcetype=Dissertations%20&%20Theses>

Contreras-Osorio, F., Guzman-Guzman, I. P., Cerda-Vega, E., Chirosa-Rios, L., Ramirez-Campillo, R., & Campos-Jara, C. (2022). Effects of the Type of Sports Practice on the Executive Functions of Schoolchildren. *Int J Environ Res Public Health*, *19*(7). <https://doi.org/10.3390/ijerph19073886>

de Bruijn, A. G. M., van der Fels, I. M. J., Renken, R. J., Konigs, M., Meijer, A., Oosterlaan, J., Kostons, D., Visscher, C., Bosker, R. J., Smith, J., & Hartman, E. (2021). Differential effects of long-term aerobic versus cognitively-engaging physical activity on children's visuospatial working memory related brain activation: A cluster RCT. *Brain Cogn*, *155*, 105812. <https://doi.org/10.1016/j.bandc.2021.105812>

Deng, L., Wu, H., Ruan, H., Xu, D., Pang, S., & Shi, M. (2024). Effects of fancy rope-skipping on motor coordination and selective attention in children aged 7-9 years: a quasi-experimental study. *Front Psychol*, *15*, 1383397. <https://doi.org/10.3389/fpsyg.2024.1383397>

Dondena, C., Riva, V., Molteni, M., Musacchia, G., & Cantiani, C. (2021). Impact of Early Rhythmic Training on Language Acquisition and Electrophysiological Functioning Underlying Auditory Processing: Feasibility and Preliminary Findings in Typically Developing Infants. *Brain Sci*, *11*(11). <https://doi.org/10.3390/brainsci11111546>

El-Sayed Atwa, A. S., El-Maksoud, G. M. A., & Mabrouk Mahgoub, E. A. E.-M. (2023). Effect of a vestibular-stimulating training program on motor skills in conjunction with cognitive aptitude of young school-aged children. *Physiotherapy Quarterly*, *31*(3), 65-79. <https://doi.org/10.5114/pq.2023.126134>

Espinosa, V. P. (2024). *Effects of Music Instruction for 3-4-Year-Old Children on Cognitive Development and Spatial Skills* Louisiana State University and Agricultural & Mechanical College]. <https://www.proquest.com/docview/3110359469?pq-origsite=gscholar&fromopenview=true&sourcetype=Dissertations%20&%20Theses>

Faraji, S., Najafabadi, M. G., Zandi, H. G., Shaw, I. (2023). Effect of Aquatic Therapy on Motor Skill and Executive Function in Children With Autism Spectrum Disorder. *South African Journal for Research in Sport Physical Education and Recreation*, *45*(2), 17-27. <https://journals.co.za/doi/full/10.10520/ejc-sport_v45_n2_a2>

Fedewa, A., Mayo, M. R., Ahn, S., & Erwin, H. (2020). A School-Based Physical Activity Intervention for Young Children: Are There Effects on Attention and Behavior? *Journal of Applied School Psychology*, *37*(4), 391-414. <https://doi.org/10.1080/15377903.2020.1858380>

Fernandes, V. R., Scipião Ribeiro, M. L., Araújo, N. B., Mota, N. B., Ribeiro, S., Diamond, A., & Deslandes, A. C. (2022). Effects of Capoeira on children's executive functions: A randomized controlled trial. *Mental Health and Physical Activity*, *22*. <https://doi.org/10.1016/j.mhpa.2022.100451>

Frikha, M., & Alharbi, R. S. (2023). Optimizing Fine Motor Coordination, Selective Attention and Reaction Time in Children: Effect of Combined Accuracy Exercises and Visual Art Activities. *Children (Basel)*, *10*(5). <https://doi.org/10.3390/children10050786>

Ghasemian Moghadam, H., & Sohrabi, M. (2024). The Effect Of Basic Taekwondo Exercises On Executive Functions Of Children With Learning Disorder. *Research in Sport Management and Motor Behavior*, *14*(27), 202-223. <https://doi.org/10.61186/jrsm.14.27.202>

Ghent, J. (2023). *A Novel Jazz Music Curriculum for Young Children: Results of A Pilot Study* University of South Florida.]. <https://www.proquest.com/docview/2847265977?pq-origsite=gscholar&fromopenview=true&sourcetype=Dissertations%20&%20Theses>

Giordano, G., & Alesi, M. (2022). Does Physical Activity Improve Inhibition in Kindergarteners? A Pilot Study. *Percept Mot Skills*, *129*(4), 1001-1013. <https://doi.org/10.1177/00315125221109216>

Gleichmann, D. C., Pinner, J. F. L., Garcia, C., Hakeem, J. H., Kodituwakku, P., & Stephen, J. M. (2022). A Pilot Study Examining the Effects of Music Training on Attention in Children with Fetal Alcohol Spectrum Disorders (FASD). *Sensors (Basel)*, *22*(15). <https://doi.org/10.3390/s22155642>

Gokdere, F., Uylas, E., Catikkas, F., Gunay, E., Ceylan, H. I., & Ozgoren, M. (2025). Integrating Kata Training into School Education: Effects on Sustained Attention and Cognitive Performance in 8-9-Year-Old Children. *Children (Basel)*, *12*(2). <https://doi.org/10.3390/children12020208>

Hao, L., Fan, Y., Zhang, X., Rong, X., Sun, Y., & Liu, K. (2023). Functional physical training improves fitness and cognitive development in 4~5 years old children. *Front Psychol*, *14*, 1266216. <https://doi.org/10.3389/fpsyg.2023.1266216>

Hashemi, A., Khodaverdi, Z., & Zamani, M. H. (2022). Effect of Wii Fit training on visual perception and executive function in boys with developmental coordination disorders: A randomized controlled trial. *Res Dev Disabil*, *124*, 104196. <https://doi.org/10.1016/j.ridd.2022.104196>

Hashempour Alooche, M., Jamebozorgi, A., Akbarzadeh Baghban, A., Hejazi-Shirmard, M., & Dehghan Manshadi, M. A. (2025). Effect of physical activities on cognition and the ability to perform a dual task in children with autism spectrum disorder: a randomized clinical trial. *J Bodyw Mov Ther*, *43*, 416-423. <https://doi.org/10.1016/j.jbmt.2025.05.015>

Hirata, G. (2023). Improving children’s executive functions: evidence fromcapoeira. *Oxford Economic Papers*, *75*(2), 490-506. <https://doi.org/10.1093/oep/gpac027>

Huang, Z., Li, L., Lu, Y., Meng, J., & Wu, X. (2024). Effects of rope skipping exercise on working memory and cardiorespiratory fitness in children with attention deficit hyperactivity disorder. *Front Psychiatry*, *15*, 1381403. <https://doi.org/10.3389/fpsyt.2024.1381403>

Jackson, R., & Meng, Y. (2024). Cognitive outcomes of the at-home brain balance program. *Front Child Adolesc Psychiatry*, *3*, 1450695. <https://doi.org/10.3389/frcha.2024.1450695>

Ji, H., Wu, S., Won, J., Weng, S., Lee, S., Seo, S., & Park, J. J. (2023). The Effects of Exergaming on Attention in Children With Attention Deficit/Hyperactivity Disorder: Randomized Controlled Trial. *Jmir Serious Games*, *11*, e40438. <https://doi.org/10.2196/40438>

Jun, C. (2023). Impacts of Soccer on Executive Function in Boys with Adhd. *Revista Brasileira de Medicina do Esporte*, *29*. <https://doi.org/10.1590/1517-8692202329012022_0469>

Jylanki, P., Saakslahti, A., & Aunio, P. (2024). Intervention effects on low performing preschoolers' early mathematical skills: Adding fundamental motor skill practice as a supporting method. *Trends Neurosci Educ*, *35*, 100227. <https://doi.org/10.1016/j.tine.2024.100227>

Kakejani, H., Farsi, A., Abdoli, B., & Hassanlouei, H. (2024). The Game-Based Training Improves Fundamental Movement Skills in Children with Down Syndrome. *International Journal of Disability, Development and Education*, *72*(1), 77-91. <https://doi.org/10.1080/1034912x.2024.2344007>

Keye, S. A., Kinder, C. J., Ragab, S., Ouzidane, M., Rich, A., Richards, K. A., & Khan, N. A. (2023). Effects of a Summer Physical Activity Program on Fitness and Cognitive Function among Children from Low Socioeconomic Households. *J Cogn Enhanc*, 1-10. <https://doi.org/10.1007/s41465-023-00266-x>

Kocak, C. V., Canli, U., Basal, V., & Aldhahi, M. I. (2024). A Randomized Controlled Trial on the Effect of Regular Dart Training on Visual Perception and Attention Level in Pre-Adolescent Children. *Healthcare (Basel)*, *12*(22). <https://doi.org/10.3390/healthcare12222272>

Kolovelonis, A., Pesce, C., & Goudas, M. (2022). The Effects of a Cognitively Challenging Physical Activity Intervention on School Children's Executive Functions and Motivational Regulations. *Int J Environ Res Public Health*, *19*(19). <https://doi.org/10.3390/ijerph191912742>

Korcz, A., Krzysztoszek, J., Bojkowski, L., Koszalka-Silska, A., Khorkova, M., Gomolysek, A., & Bronikowski, M. (2024). The effects of the 'active before school' programme on the academic skills of 8-9-year-old children: a physically and cognitively engaging intervention. *Front Public Health*, *12*, 1402901. <https://doi.org/10.3389/fpubh.2024.1402901>

Kurnaz, M., & Altinkök, M. (2023). Exploring the impact of coordination-based movement education practices on fundamental motor movements and attention skills in 5-6-year-old children. *Journal of Physical Education and Sport*, *23*(10), 2567-2583. <https://doi.org/10.7752/jpes.2023.10295>

Lee, M. W., Yang, N. J., Mok, H. K., Yang, R. C., Chiu, Y. H., & Lin, L. C. (2024). Music and movement therapy improves quality of life and attention and associated electroencephalogram changes in patients with attention-deficit/hyperactivity disorder. *Pediatr Neonatol*, *65*(6), 581-587. <https://doi.org/10.1016/j.pedneo.2023.11.007>

Liu, Z. M., Chen, C. Q., Fan, X. L., Lin, C. C., & Ye, X. D. (2022). Usability and Effects of a Combined Physical and Cognitive Intervention Based on Active Video Games for Preschool Children. *Int J Environ Res Public Health*, *19*(12). <https://doi.org/10.3390/ijerph19127420>

Ludyga, S., Hanke, M., Leuenberger, R., Bruggisser, F., Puhse, U., Gerber, M., Lemola, S., Capone-Mori, A., Keutler, C., Brotzmann, M., & Weber, P. (2023). Martial Arts and Cognitive Control in Children with Attention-Deficit Hyperactivity Disorder and Children Born Very Preterm: A Combined Analysis of Two Randomized Controlled Trials. *Med Sci Sports Exerc*, *55*(5), 777-786. <https://doi.org/10.1249/MSS.0000000000003110>

Magistro, D., Cooper, S. B., Carlevaro, F., Marchetti, I., Magno, F., Bardaglio, G., & Musella, G. (2022). Two years of physically active mathematics lessons enhance cognitive function and gross motor skills in primary school children. *Psychology of Sport and Exercise*, *63*. <https://doi.org/10.1016/j.psychsport.2022.102254>

Masini, A., Marini, S., Ceciliani, A., Barone, G., Lanari, M., Gori, D., Bragonzoni, L., Toselli, S., Stagni, R., Bisi, M. C., Sansavini, A., Tessari, A., & Dallolio, L. (2023). The effects of an active breaks intervention on physical and cognitive performance: results from the I-MOVE study. *J Public Health (Oxf)*, *45*(4), 919-929. <https://doi.org/10.1093/pubmed/fdad102>

Mazzoli, E. S., J. Pesce, C. Teo, W. P, Rinehart N, May T, Barnett. (2021). Effects of classroom‐based active breaks on cognition, sitting and on‐task behaviour in children with intellectual disability: a pilot study. *Journal of Intellectual Disability Research*, *65*(5), 464-488. <https://doi.org/10.1111/jir.12826>

McGrath, K. (2024). Effects of cognitively engaging physical activity on preschool children's cognitive outcomes. *JOPERD: The Journal of Physical Education, Recreation & Dance*, *95*(2), 64-64. <https://doi.org/10.1080/07303084.2023.2292456>

McLellan, G., Arthur, R., Donnelly, S., Bakshi, A., Fairclough, S. J., Taylor, S. L., & Buchan, D. S. (2022). Feasibility and Acceptability of a Classroom-Based Active Breaks Intervention for 8-12-Year-Old Children. *Res Q Exerc Sport*, *93*(4), 813-824. <https://doi.org/10.1080/02701367.2021.1923627>

Méndez-Giménez, A. M.-G., & Pallasá-Manteca, M. (2023). Efecto de los descansos activos sobre procesos atencionales y la regulación motivacional en escolares. *Apunts Educación Física y Deportes*(151), 49-57. <https://doi.org/10.5672/apunts.2014-0983.es.(2023/1).151.05>

Miller, A. L., Palmer, K. K., Wang, L., Wang, C., Riley, H. O., McClelland, M. M., & Robinson, L. E. (2023). Mastery-oriented motor competence intervention improves behavioral but not cognitive self-regulation in head start preschoolers: Randomized controlled trial results. *Scand J Med Sci Sports*, *33*(5), 725-736. <https://doi.org/10.1111/sms.14294>

Ng-Knight, T., Gilligan-Lee, K. A., Massonnie, J., Gaspard, H., Gooch, D., Querstret, D., & Johnstone, N. (2022). Does Taekwondo improve children's self-regulation? If so, how? A randomized field experiment. *Dev Psychol*, *58*(3), 522-534. <https://doi.org/10.1037/dev0001307>

Nie, P., Wang, C., Rong, G., Du, B., Lu, J., Li, S., Putkinen, V., Tao, S., & Tervaniemi, M. (2021). Effects of Music Training on the Auditory Working Memory of Chinese-Speaking School-Aged Children: A Longitudinal Intervention Study. *Front Psychol*, *12*, 770425. <https://doi.org/10.3389/fpsyg.2021.770425>

Oliveira, D. N., Silva, E. C. M., Barboza, L. L. S., Thuany, M., Araujo, R. H. O., Silva, R. J. S., Gomes, T. N., Schmitz, H., Tejada, J., & Silva, D. R. (2023). Effects of two years of physically active lessons on cognitive indicators in children. *Sci Rep*, *13*(1), 8774. <https://doi.org/10.1038/s41598-023-35644-0>

Parsamajd, F., & Teymori, S. (2024). Karate Kata training: A promising intervention for behavioral problems in elementary school children. *J Exp Child Psychol*, *248*, 106058. <https://doi.org/10.1016/j.jecp.2024.106058>

Pinelli, M., Portrat, S., Dessus, P., Joet, G., Eichenlaub, J. B., Brugniaux, J., Palluel, E. (2025). What a shot! Effect of biathlon and badminton on attention of preteens in middle schools: a longitudinal study. *International Journal of Sport and Exercise Psychology*, 1-22. <https://doi.org/https://doi.org/10.1080/1612197X.2025.2477144>

Plaza, P., Tapia, O. A., & Mello, J. B. . (2024). Effects of slackline training during active breaks and free-time activities break at school on children's attention: a comparison of two-intervention methods. . *Retos: nuevas tendencias en educación física, deporte y recreación*, *56*, 909-916. <https://recyt.fecyt.es/index.php/retos/index>

Price-Mohr, R., & Price, C. (2021). Learning to Play the Piano Whilst Reading Music: Short-Term School-Based Piano Instruction Improves Memory and Word Recognition in Children. *International Journal of Early Childhood*, *53*(3), 333-344. <https://doi.org/10.1007/s13158-021-00297-5>

Qiu, H., & Liang, X. (2024). Change in Sleep Latency as a Mediator of the Effect of Physical Activity Intervention on Executive Functions Among Children with ADHD: A Secondary Analysis from a Randomized Controlled Trial. *J Autism Dev Disord*, *54*(8), 3069-3077. <https://doi.org/10.1007/s10803-023-06018-2>

Rahe, M., Schaefer, J., Schürmann, L., & Jansen, P. (2024). Influence of Boxing Training on Self-Concept and Mental Rotation Performance in Children. *Journal of Cognitive Enhancement*. <https://doi.org/10.1007/s41465-024-00297-y>

Richard Williams, N., Hurt-Thaut, C., Brian, J., Tremblay, L., Pranjic, M., Teich, J., Tan, M., Kowaleski, J., & Thaut, M. (2024). Improved motor skills in autistic children after three weeks of neurologic music therapy via telehealth: a pilot study. *Front Psychol*, *15*, 1355942. <https://doi.org/10.3389/fpsyg.2024.1355942>

Rodriguez-Negro, J., Pesola, J. A., & Yanci, J. (2022). Can different physical education programs produce specific developments in psychological responses and cognitive functions? An ecological intervention in school-age children. *Br J Educ Psychol*, *92*(4), 1687-1698. <https://doi.org/10.1111/bjep.12533>

Rodríguez-Negro, J., & Yanci, J. (2021). Effects of two different physical education instructional models on creativity, attention and impulse control among primary school students. *Educational Psychology*, *42*(6), 787-799. <https://doi.org/10.1080/01443410.2021.1988059>

Rosenstreich, E. S., E. Sharir, T. (2022). The Effects of Mindful Movement Intervention on Academic and Cognitive Abilities Among Kindergarten Children. *Early Childhood Education Journal*. <https://doi.org/10.1007/s10643-020-01150-5>

Sabaghi, A., Ebrahimi, B., Yousofvand, N., & Hoseini, R. (2025). Comparative effects of moderate-intensity continuous training and high-intensity interval training on ADHD symptoms and behavioral inhibition in children. *Eur J Pediatr*, *184*(2), 183. <https://doi.org/10.1007/s00431-025-06022-x>

Sendil, A. M., Canli, U., Sheeha, B. B., Alkhamees, N. H., Batrakoulis, A., & Al-Mhanna, S. B. (2024). The effects of structured coordinative exercise protocol on physical fitness, motor competence and inhibitory control in preschool children. *Sci Rep*, *14*(1), 28462. <https://doi.org/10.1038/s41598-024-79811-3>

Shahna, M., Hejazi Dinan, P., & Ahmadpour, A. (2024). The Effect of Fundamental Motor Skill Training by “Teaching Game for Understanding” Approach on Motor and Cognitive Skills in Children With Attention-Deficit/Hyperactivity Disorder. *Scientific Journal of Rehabilitation Medicine*, *13*(02), 392-405. <https://doi.org/10.32598/sjrm.13.2.3225>

Slosar, L., de Bruin, E. D., Fontes, E. B., Plevnik, M., Pisot, R., Simunic, B., & Marusic, U. (2021). Additional Exergames to Regular Tennis Training Improves Cognitive-Motor Functions of Children but May Temporarily Affect Tennis Technique: A Single-Blind Randomized Controlled Trial. *Front Psychol*, *12*, 611382. <https://doi.org/10.3389/fpsyg.2021.611382>

Sperling, J., Lee, V. K., Schmid, L., Gray, M., & Kim, J. Y. (2023). A randomized study of El Sistema-inspired youth music education programming: Evidence addressing executive function and social–emotional learning. *Psychology of Aesthetics, Creativity, and the Arts*. <https://doi.org/10.1037/aca0000596>

Su, W. C., Srinivasan, S., & Bhat, A. N. (2025). Effects of Movement and Sedentary Play interventions on executive functioning and their relationships with sensory, repetitive, and negative behaviors of children with ASD - a pilot RCT. *Disabil Rehabil*, 1-9. <https://doi.org/10.1080/09638288.2025.2465600>

Sun, F., Fang, Y., Ho, Y. F., Chow, G. C., Yang, Y., Huang, K., Yu, C. C., Liu, D., Wong, S. H., Siu, P. M., & Cooper, S. B. (2024). Effectiveness of a game-based high-intensity interval training on executive function and other health indicators of children with ADHD: A three-arm partially-blinded randomized controlled trial. *J Exerc Sci Fit*, *22*(4), 408-416. <https://doi.org/10.1016/j.jesf.2024.09.001>

Suppalarkbunlue, W., Chutabhakdikul, N., Lertladaluck, K., & Moriguchi, Y. (2022). Promoting Inhibitory Control in Preschool Children Through Music-Movement Activities in the Classroom. *Journal of Research in Childhood Education*, *37*(2), 275-291. <https://doi.org/10.1080/02568543.2022.2111482>

Tajari, S. N., Gholami, S., Rostami, R., Trabelsi, K., & Taheri, M. (2023). The effect of perceptual-motor exercise on temporal dynamics of cognitive inhibition control in children with developmental coordination disorder. *Mental Health and Physical Activity*, *24*. <https://doi.org/10.1016/j.mhpa.2022.100495>

Takehara, K., Togoobaatar, G., Kikuchi, A., Lkhagvasuren, G., Lkhagvasuren, A., Aoki, A., Takemune, F., Shagdar, B., Suwabe, K., Mikami, M., Mori, R., Soya, H. (2021). Exercise intervention for academic achievement among children: a randomized controlled trial. *Pediatrics*, *148*(5). <https://doi.org/https://doi.org/10.1542/peds.2021-052808>

Teicher, M. H., Bolger, E., Hafezi, P., Garcia, L. C. H., McGreenery, C. E., Weiser, L., Ohashi, K., & Khan, A. (2023). Open assessment of the therapeutic and rate-dependent effects of brain balance center(R) and interactive metronome(R) exercises on children with attention deficit hyperactivity disorder. *Psychiatry Res*, *319*, 114973. <https://doi.org/10.1016/j.psychres.2022.114973>

Tocci, N., Scibinetti, P., Mazzoli, E., Mavilidi, M. F., Masci, I., Schmidt, M., & Pesce, C. (2022). Giving Ideas Some Legs or Legs Some Ideas? Children's Motor Creativity Is Enhanced by Physical Activity Enrichment: Direct and Mediated Paths. *Front Psychol*, *13*, 806065. <https://doi.org/10.3389/fpsyg.2022.806065>

Torabi, F., & Abasi Hormozi, S. (2024). Comparison of the Impact of Body Percussion Exercises on Executive and Balance Performance in Intellectually Disabled and Healthy Girls of Ramhormoz City. *International Journal of Sport Studies for Health*, *7*(1), 45-53. <https://doi.org/10.61838/kman.intjssh.7.1.6>

Tseng, Y. H., Chao, H. H., & Hung, C. L. (2022). Effect of a Strategic Physical Activity Program on Cognitive Flexibility among Children with Internet Addiction: A Pilot Study. *Children (Basel)*, *9*(6). <https://doi.org/10.3390/children9060798>

Urena Ortin, N., Madinabeitia Cabrera, I., & Alarcon Lopez, F. (2024). Effect of the ActivaMotricidad Program on Improvements in Executive Functions and Interpersonal Relationships in Early Childhood Education. *J Funct Morphol Kinesiol*, *9*(4). <https://doi.org/10.3390/jfmk9040231>

Vasilopoulos, F., & Dumontheil, I. (2025). Effect of a creative dance-based physical education intervention on primary school children’s creativity and self-regulation: A pilot study. *Psychology of Aesthetics, Creativity, and the Arts*. <https://doi.org/10.1037/aca0000732>

Vazou, S., & Mavilidi, M. F. (2021). Cognitively Engaging Physical Activity for Targeting Motor, Cognitive, Social, and Emotional Skills in the Preschool Classroom: The Move for Thought preK-K Program. *Front Psychol*, *12*, 729272. <https://doi.org/10.3389/fpsyg.2021.729272>

Wang, H., Ge, W., Zhu, C., Sun, Y., & Wei, S. (2022). How pom cheerleading improves the executive function of preschool children: the mediating role of speed and agility. *BMC Psychol*, *10*(1), 234. <https://doi.org/10.1186/s40359-022-00944-z>

Wang, X., & Li, H. (2025). Effects of Different Traditional Chinese Mind-Body Exercises on Learning Abilities, Executive Functions, and Brain Connectivity in Children with Learning Difficulties. *Behav Sci (Basel)*, *15*(3). <https://doi.org/10.3390/bs15030303>

Williams, K. E., Bentley, L. A., Savage, S., Eager, R., & Nielson, C. (2023). Rhythm and movement delivered by teachers supports self-regulation skills of preschool-aged children in disadvantaged communities: A clustered RCT. *Early Childhood Research Quarterly*, *65*, 115-128. <https://doi.org/10.1016/j.ecresq.2023.05.008>

Zanto, T. P., Giannakopoulou, A., Gallen, C. L., Ostrand, A. E., Younger, J. W., Anguera-Singla, R., Anguera, J. A., & Gazzaley, A. (2024). Digital rhythm training improves reading fluency in children. *Dev Sci*, *27*(3), e13473. <https://doi.org/10.1111/desc.13473>

Zask, A., Pattinson, M., Ashton, D., Ahmadi, M., Trost, S., Irvine, S., Stafford, L., Delbaere, K., & Adams, J. (2023). The effects of active classroom breaks on moderate to vigorous physical activity, behaviour and performance in a Northern NSW primary school: A quasi-experimental study. *Health Promot J Austr*, *34*(4), 799-808. <https://doi.org/10.1002/hpja.688>

Zhang, J., Lu, J., Sun, Y., & Li, J. (2024). Recreational gymnastics exercise of moderate intensity enhances executive function in Chinese preschoolers: A randomized controlled trial. *Psych J*, *13*(6), 954-965. <https://doi.org/10.1002/pchj.786>

Zhang, J. Y., Shen, Q. Q., Wang, D. L., Hou, J. M., Xia, T., Qiu, S., Wang, X. Y., Zhou, S. B., Yang, W. W., Heng, S. Y., Lu, C. C., Cui, L., & Yin, H. C. (2022). Physical activity intervention promotes working memory and motor competence in preschool children. *Front Public Health*, *10*, 984887. <https://doi.org/10.3389/fpubh.2022.984887>

Zhang, M., Garnier, H., Qian, G., & Li, S. (2023). Effect of 11 Weeks of Physical Exercise on Physical Fitness and Executive Functions in Children. *Children (Basel)*, *10*(3). <https://doi.org/10.3390/children10030485>

Zhang, Y., Tian, H., Tao, Y., Li, Y., Wang, D., & Qin, L. (2025). A study on the effects of three game intervention programs on executive functions of preschool autistic children. *Int J Dev Disabil*, *71*(1), 168-178. <https://doi.org/10.1080/20473869.2023.2215606>

Zhao, P., Chen, K., Zhu, G., Li, H., Chen, S., Hu, J., Huang, L., Liu, X., & Guo, L. (2024). Effects of aquatic exercise intervention on executive function and brain-derived neurotrophic factor of children with autism spectrum disorder. *Res Dev Disabil*, *150*, 104759. <https://doi.org/10.1016/j.ridd.2024.104759>

Zhong, X., Wang, C., Xu, M., Yuan, X., & Jiang, C. (2024). Physical training improves inhibitory control in children aged 7-12 years: An fNIRS study. *Behav Brain Res*, *463*, 114902. <https://doi.org/10.1016/j.bbr.2024.114902>

Zinelabidine, K., Elghoul, Y., Jouira, G., & Sahli, S. (2022). The Effect of an 8-Week Aerobic Dance Program on Executive Function in Children. *Percept Mot Skills*, *129*(1), 153-175. <https://doi.org/10.1177/00315125211058001>
