# Supplementary material for: A Systematic Review and Evidence Gap Map Evaluation of Rhythmic and/or Complex Movement Interventions and Child Cognitive Outcomes
Source: Clin Child Fam Psychol Rev. 2025 Oct 11;28(4):912–29. doi: 10.1007/s10567-025-00547-1 (PMC12660398; doi:10.1007/s10567-025-00547-1)
Supplement: Supplementary file 4 — Supplementary file4 (DOCX 51 KB) [file 10567_2025_547_MOESM4_ESM.docx]

**Supplementary Materials D**

**D.1 Ongoing or Incomplete Studies**

Actrn (2021). The Best Start: the effect of teacher-child interactions on young children’s oral language and self-regulation http://www.who.int/trialsearch/Trial2.aspx?TrialID=ACTRN12621000845831

Actrn (2017). Effectiveness of GAME (Goals Activity Motor Enrichment) for infants at high risk of cerebral palsy http://www.who.int/trialsearch/Trial2.aspx?TrialID=ACTRN12617000006347

Actrn (2018). A Physical Activity Program in School-Aged Children with Attention Deficit Hyperactivity Disorder http://www.who.int/trialsearch/Trial2.aspx?TrialID=ACTRN12618001822279

Actrn (2019). A brief intervention to increase child physical activity levels in childcare: the Everybody Energise trial http://www.who.int/trialsearch/Trial2.aspx?TrialID=ACTRN12619000042145

Actrn (2021). The Kick-Smart Homework program: a randomised feasibility trial evaluating the feasibility and efficacy of a primary-school based martial arts homework program integrating mathematics and physical fitness https://trialsearch.who.int/Trial2.aspx?TrialID=ACTRN12621000522819

Alon, D., Sousa, C. V., Baranowski, T., Barreira, T. V., Cabrera-Perez, R., Chiu, K., ... & Lu, A. S. (2020). The impact of narratives and active video games on long-term moderate-to-vigorous physical activity: A randomized controlled trial protocol. Contemporary clinical trials, 96, 106087.

Arenas, D., Bodi-Torralba, M., Oliver, A., Cantallops, J., Ponseti, F. J., Palou-Sampol, P., Collado, J. A., Fl&oacute;rez, I., Galvez-Pol, A., Terrasa, J. L., et al. (2024). Effects of active breaks on educational achievement in children with and without ADHD: study protocol and rationale of the Break4Brain project Frontiers in psychology, 15, 1451731

Armstrong, B., Trude, A. C., Johnson, C., Castelo, R. J., Zemanick, A., Haber-Sage, S., ... & Black, M. M. (2019). CHAMP: A cluster randomized-control trial to prevent obesity in child care centers. Contemporary clinical trials, 86, 105849.

Chi, Ctr Trc (2010). Effect of Physical Activity on Cognitive Function in Children with Attention-Deficit Hyperactivity Disorder http://www.who.int/trialsearch/Trial2.aspx?TrialID=ChiCTR-TRC-10001127

ChiCtr (2022). Experimental study on the effect of skipping exercise on executive function and EEG characteristics of autistic children https://trialsearch.who.int/Trial2.aspx?TrialID=ChiCTR2200067127

ChiCtr (2024). A Randomized Controlled Trial of Physical Activity Intervention on Neurocognitive Performance, Mental Health, and Behavioural Outcomes of Children with Attention Deficit Hyperactivity Disorder https://trialsearch.who.int/Trial2.aspx?TrialID=ChiCTR2400080109

ChiCtr (2021). Experimental study of Cheerleading exercise intervention on improving executive function of deaf mute children http://www.who.int/trialsearch/Trial2.aspx?TrialID=ChiCTR2100043

Domínguez-Muñoz, A., Carlos-Vivas, J., Barrios-Fernandez, S., Adsuar, J. C., Morenas-Martín, J., Garcia-Gordillo, M. A., & Domínguez-Muñoz, F. J. (2021). Pedagogical proposal of tele-exercise based on “square stepping exercise” in preschoolers: study protocol. International Journal of Environmental Research and Public Health, 18(16), 8649.

Irct138904204264N (2010). Physical education effects in autistic children http://www.who.int/trialsearch/Trial2.aspx?TrialID=IRCT138904204264N3

Irct20150519022323N (2017). The effect of Perceptual motor Exercises along with music and Vitamin D3 Supplementation in children with autism spectrum disorder http://www.who.int/trialsearch/Trial2.aspx?TrialID=IRCT20150519022323N2

Irct2016031627075N (2016). The effect of exercise training program on children with Attention Deficit / Hyperactivity http://www.who.int/trialsearch/Trial2.aspx?TrialID=IRCT2016031627075N1

Irct2016031727092N (2017). The effect of aerobic exercise on learning disorder treatment http://www.who.int/trialsearch/Trial2.aspx?TrialID=IRCT2016031727092N1

Irct20181222042071N (2019). The effect of active video games on cognitive and motor skills in children with developmental coordination disorder http://www.who.int/trialsearch/Trial2.aspx?TrialID=IRCT20181222042071N1

Irct20200125046251N (2020). The effect of perceptual-motor training and mindfulness in hyperactive children http://www.who.int/trialsearch/Trial2.aspx?TrialID=IRCT20200125046251N1

Irct20220718055489N (2023). The effectiveness of perceptual motor exercises compared to motor activity with video modeling on motor development and executive function of children with learning disorders https://trialsearch.who.int/Trial2.aspx?TrialID=IRCT20220718055489N1

Irct20231118060101N (2023). The Intervention of Music-Based Education on Executive Functions in High-Functioning Children with Autism https://trialsearch.who.int/Trial2.aspx?TrialID=IRCT20231118060101N1

Irct20240624062244N (2024). The effectiveness of family-oriented rhythmic movement therapy in managing the behavior of restless and hyperactive children https://trialsearch.who.int/Trial2.aspx?TrialID=IRCT20240624062244N1

Irct20240712062404N (2024). the effect of square stepping exercise on the motor and cognitive factors of autism spectrum children https://trialsearch.who.int/Trial2.aspx?TrialID=IRCT20240712062404N1

Isrctn (2019). Moving Maths &acirc;&euro;&rdquo; the effects of physically active math lessons http://www.who.int/trialsearch/Trial2.aspx?TrialID=ISRCTN71844310

James, C. E., Tingaud, M., Laera, G., Guedj, C., Zuber, S., Palazzo, R. Diambrini, Vukovic, S., Richiardi, J., Kliegel, M., Marie, D. (2024). Cognitive enrichment through art: a randomized controlled trial on the efect of music or visual arts group practice on cognitive and brain development of young children BMC Complementary Medicine & Therapies, 24(1), 1-22

Jeppesen, L. S., Damsgaard, L., Stolpe, M. N., Melcher, J. N. S., Wienecke, J., Nielsen, G., Smedegaard, S., Henriksen, A. H., Hansen, R. A., Hillman, C. H., et al. (2024). Study protocol for the ACTIVE SCHOOL study investigating two different strategies of physical activity to improve academic performance in Schoolchildren BMC pediatrics, 24(1), 174

Kao, S. C., Fu, H. L., Wang, C. H., Chou, C. C. (2024). Maintained cognitive gains after 10 weeks of cognitively engaging exercise via reducing body fat in overweight children International Journal of Sport and Exercise Psychology.

Mazzetti, C., Gonzales Damatac, C., Sprooten, E., Ter Huurne, N., Buitelaar, J. K., & Jensen, O. (2022). Dorsal‐to‐ventral imbalance in the superior longitudinal fasciculus mediates methylphenidate’s effect on beta oscillations in ADHD. Psychophysiology, 59(5), e14008.

Morgan, C., Badawi, N., Boyd, R. N., Spittle, A. J., Dale, R. C., Kirby, A., ... & GAME study team. (2023). Harnessing neuroplasticity to improve motor performance in infants with cerebral palsy: a study protocol for the GAME randomised controlled trial. BMJ open, 13(3), e070649.

Nct (2021). Balance Auditory Vision Training (Bal-A-Vis-X) and Aerobic Training in Children With Autism Spectrum Disorder https://clinicaltrials.gov/show/NCT05013528

Nct (2023). Feasibility and Fidelity of a Gross Motor-based Physical Activity Intervention on Cognition in Preschool-age Children https://clinicaltrials.gov/ct2/show/NCT05949866

Nct (2024). Comparison Between Plyometrics and Calisthenics on Executive Function in School Going Children https://clinicaltrials.gov/ct2/show/NCT06770192

Nct (2024). Coordination-based Exercise Intervention in Preschool Children https://clinicaltrials.gov/ct2/show/NCT06405854

Nct (2024). Effect of a School Intervention With Physical Activity on Executive Functions https://clinicaltrials.gov/ct2/show/NCT06496503

Nct (2024). Physical Activity Intervention Effects on Executive Function, Physical Activity and Sedentary Behaviour in Children https://clinicaltrials.gov/ct2/show/NCT06376864

Nct (2024). The Effects of 12-Week High-intensity Interval Training With Mindfulness-based Recovery on Executive Function, Fitness, and Mindfulness in Children https://clinicaltrials.gov/ct2/show/NCT06258941

Nct (2024). The Purpose of This Research is to Investigate the Effects of Aerobic Exercise on Executive Function in Obese Children https://clinicaltrials.gov/ct2/show/NCT06510920

Nct (2019). Impact of Physical Activity on Cognitive Outcomes in Youth With Pediatric-Onset Multiple Sclerosis (POMS) https://clinicaltrials.gov/show/NCT03933020

Nct (2019). Implementation and Evaluation of a Before School Physical Activity Program in Revere, MA https://clinicaltrials.gov/show/NCT03805295

Nct (2019). Karate or Kung Fu? https://clinicaltrials.gov/show/NCT04120896

Nct (2020). Home-Based Exergaming Intervention https://clinicaltrials.gov/show/NCT04540523

Nct (2020). Exercise in Children Attention Deficit Hyperactivity Disorder https://clinicaltrials.gov/show/NCT04279652

Ntr (2016). What are the effects of 10 minutes of exercise breaks during the school curriculum on the cognitive and academic performance in children http://www.who.int/trialsearch/Trial2.aspx?TrialID=NTR5993

Odelia van, Stryp, Duncan, Michael J., Africa, Eileen (2024). The Effect of Active Brain-Breaks on Fundamental Movement Skills and Executive Functioning of Grade One Children in Cape Town, South Africa Early Child Development and Care, 194(1), 102-117

Qi, S. I., Yinge, Chen, Ao, N. I. E., Qifei, Chen, Lefang, X. U., Cai, Huang (2024). Experimental Study of Effectiveness of Gymnastic Games Intervention on Promoting Preschoolers&#039; self-control and Motor Coordination Journal of Tianjin University of Sport / Tianjin Tiyu Xueyuan Xuebao, 39(1), 108-114

RBR (2020). Effect of martial arts on cognition, physical abilities and school performance in students http://www.who.int/trialsearch/Trial2.aspx?TrialID=RBR-6k7pkv

Robinson, L. E., Palmer, K. K., Wang, L., Scott-Andrews, K. Q., Chinn, K. M., Sur, I., Wengrovius, C., Meng, E., Veldman, S. L. C., Miller, A. L. (2023). Protocol for a cluster randomized clinical trial of a mastery-climate motor skills intervention, Children&#039;s Health Activity and Motor Program (CHAMP), on self-regulation in preschoolers PloS one, 18(3), e0282199

Sepehri Bonab, H., Ebrahimi Sani, S., Behzadnia, B. (2024). The Impact of Virtual Reality Intervention on Emotion Regulation and Executive Functions in Autistic Children Games for health journal

Solis-Urra, P., Olivares-Arancibia, J., Suarez-Cadenas, E., Sanchez-Martinez, J., Rodríguez-Rodríguez, F., Ortega, F. B., ... & Cristi-Montero, C. (2019). Study protocol and rationale of the “Cogni-action project” a cross-sectional and randomized controlled trial about physical activity, brain health, cognition, and educational achievement in schoolchildren. BMC pediatrics, 19(1), 260.

Tctr (2023). Effectiveness of music training on executive function and attention in middle school-aged children with ADHD : a randomized controlled study https://trialsearch.who.int/Trial2.aspx?TrialID=TCTR20230109006

Tctr (2023). The effect of cognitively engaging movement games on perceptual discrimination, physical fitness, body fat, and physical activity time in overweight children https://trialsearch.who.int/Trial2.aspx?TrialID=TCTR20230202007

Tctr (2017). Effectiveness of Computerized Working Memory Training Versus Tae Kwon Do in Treatment of Working Memory Problem: a Randomized Controlled Trial http://www.who.int/trialsearch/Trial2.aspx?TrialID=TCTR20170712001

Tctr (2018). Movement game activity enhances executive functions in overweight children: a randomized controlled trial http://www.who.int/trialsearch/Trial2.aspx?TrialID=TCTR20180414002

Umin (2016). Effects of combination exercises on electroencephalography and frontal lobe executive function measures in children with ADHD https://trialsearch.who.int/Trial2.aspx?TrialID=JPRN-UMIN000023215

Varigonda, A. L. (2022). 3.24 Exergaming Using Obie&reg; and its Effect in Improving Executive Function Deficits in Children With Fetal Alcohol Spectrum Disorder and Comorbid ADHD: An Exploratory Study Journal of the American Academy of Child and Adolescent Psychiatry, 61(10), S235

Yanqin, W. U., Quan, F. U. (2024). Effects of Physical Activity on Children&#039;s Attention and Cognitive Flexibility Journal of Tianjin University of Sport / Tianjin Tiyu Xueyuan Xuebao, 39(2), 233-240

**D.2 Studies Awaiting Classification (LOTE or Inaccessible)**

Acebes-de Pablo, A., Carabias-Galindo, D. (2016). Music Therapy as an Integrative Tool for Pupils with Attention-Deficit Hyperactivity Disorder (ADHD) in the Music Classrooms at Elementary Schools REVISTA ELECTRONICA DE LEEME, #volume#(38), 1-16

Alves, F. S. (2016). QUALITATIVE EVALUATION EXERCISES WITH RHYTHM, BODY EXPRESSION AND DANCE IN PHYSICAL EDUCATION TRAINING MOVIMENTO, 22(1), 75-88

Aryabkina, I., Tenyukova, G., Medvedeva, I., Khrisanova, E., Lepeshkina, L. (2019). SOCIALIZATION OF YOUNG SCHOOL-AGE CHILDREN BY MEANS OF MUSICAL FOLKLORE AMAZONIA INVESTIGA, 8(21), 389-398

Balci, G., Atasavun Uysal, S., Esen, T. Ç (2018). The influence of the sensory profile and the capacity of physical activity on the community participation of the children with attention deficit and hiperactivity disorder Fizyoterapi Rehabilitasyon, 29(2), S31-S32

Banevičiūtė, Birutė (2007). Šokio gebėjimų raiškos ypatumai ankstyvos paauglystės metais. (Lithuanian) THE CHARACTERSITICS OF DANCE SKILLS IN EARLY ADOLESCENCE. (English), 19(#issue#), 82-92

Ben√≠tez, Mar√≠a Ang√©lica, Ver√≥nika Diaz, Abrahan, Shifres, Favio, Justel, Nadia (2024). The Effect of Active and Receptive Musical Training on Emotional Memory in Preschoolers Psykhe, 33(2), n/a

Benitez, M. A., Abrahan, V. M. D., Justel, N. R. (2017). Benefits of music training in child development: a systematic review REVISTA INTERNACIONAL DE EDUCACION MUSICAL, 5(#issue#), 61-69

Benitez, M., Abrahan, V. D., Sarli, L., Bossio, M., Justel, N. (2018). Music classes enhance memory in preschoolers CUADERNOS DE NEUROPSICOLOGIA-PANAMERICAN JOURNAL OF NEUROPSYCHOLOGY, 12(2), #Pages#

Blickle, E., Van Wiknen, H. (2009). [Relationships between physical activity and cognitive functions in preschool and primary school age. Importance for the physical therapy under close observation of the concentration effect] Pt Zeitschrift fur Physiotherapeuten, 61(6), 514-520

Buber, A., Yilanli, M., Basay, O., Celik, Z., Tuncer, O. F., Avunduk, S., Topbasoglu, T., Celik, E., Herken, H. (2016). School based combined exercise and computer training program may improve working memory in ADHD children Klinik Psikofarmakoloji Bulteni, 26(#issue#), S87

Cardeal, C. M., Pereira, L. A., Silva, P. F., França, N. M. (2013). Efeito de um programa escolar de estimulação motora sobre desempenho da função executiva e atenção em crianças Motricidade, 9(3), 47-59

Chirosa Ríos, Luis Javier, Hernández Mendo, Antonio, López Walle, Jeanette Magnolia, Reigal Garrido, Rafael E., Ruiz de Mier, Rocío Juárez, Martín Martínez, Inmaculada (2016). Effects of a small sided games program on executive function in a sample of adolescent girls. / Efectos de un programa de juegos reducidos sobre la función ejecutiva en una muestra de chicas adolescentes Retos: Nuevas Perspectivas de Educación Física, Deporte y Recreación, 30(#issue#), 1-1

Čoh, Milan (2020). MOTOR AND INTELLECTUAL DEVELOPMENT IN CHILDREN: A REVIEW. / MOTORIČKI I INTELEKTUALNI RAZVOJ DECE: PREGLED Facta Universitatis: Series Physical Education & Sport, 18(3), 515-523

da Silva Cunha, José Henrique, Câmara Gradim, Luma Carolina, Denúbila Costa, Jacqueline, Ferreira Andrade, Patrícia, Pompeu de Oliveira, Natasha, Pinto, Ana Cláudia (2015). A experiência da Terapia Ocupacional com contação de histórias em uma instituição educacional Cadernos de Terapia Ocupacional da UFSCar, 23(1), 221-225

De Freitas, Jhonny De Vasconcelos, Venegas, Marilex Pérez (2016). ConcentrArte: una propuesta de intervención para niños venezolanos diagnosticados con Trastorno de Déficit de Atención/ConcentrArte: an intervention proposal for Venezuelan children diagnosed with Attention Deficit Disorder Arteterapia, 11(#issue#), 293-307

Ericsson, Ingegerd Renate Hillevi (2003). Motorik, koncentrationsförmåga och skolprestationer: En interventionsstudie i skolår 1–3 #journal#, #volume#(#issue#), 238

Fomina, N. A., Maksimova, S. Y., Propisnova, E. P. (2016). Child's intellectual development during music motor activities Teoria i Praktika Fiziceskoj Kul'tury, 10(#issue#), 1-5

Günay, Fatma, Baydaş, Özlem, Karakuş, Türkan, Göktaş, Yüksel (2014). İlköğretim Öğrencilerinin 3B Sanal Dünyada Kış Sporlarını Öğrenmeye Yönelik Algıları. (Turkish) Perceptions of Elementary Students Towards Learning Wintersports in 3D Virtual Worlds. (English), 33(2), 664-675

Haffner, J., Roos, J., Goldstein, N., Parzer, P., Resch, F. (2006). The effectiveness of body-oriented methods of therapy in the treatment of attention-deficit hyperactivity disorder (ADHD): results of a controlled pilot study Zeitschrift fur Kinder- und Jugendpsychiatrie und Psychotherapie, 34(1), 37‐47

Hendry, J., Kerr, R. (1983). Communication through physical activity for learning disabled children. / La communication par l' activite physique pour des enfants en retard scolaire Perceptual & Motor Skills, 56(1), 155-158

Henn Brandl, Carmem Elisa, Brandl Neto, Inácio (2015). A IMPORTÂNCIA DO PROFESSOR DE EDUCAÇÃO FÍSICA NOS ANOS INICIAIS DO ENSINO FUNDAMENTAL Caderno de Educação Física e Esporte, 13(2), 1-13

Ison, Mirta Susana (2011). Intervention program for the improvement of attention abilities in Argentinean school children International Journal of Psychological Research, 4(2), 72-79

Jareckaitė, Stanislava (2008). Aukštesniqųqų klasių mokinių muzikinis-estetinis ugdymas chorine veikla. (Lithuanian) ACTIVITY PECULIARITIES OF MUSICAL-AESTHETICAL TRAINING OF CHORAL SINGING OF HIGHER CLASSES STUDENTS. (English), 21(#issue#), 117-127

Kiselev, S. (2019). Motor sequencing training has positive effect on motor and executive functions in children with ADHD Journal of the neurological sciences, 405(#issue#), 10

Mato-Vazquez, D., Chao-Fernandez, R., Chao-Fernandez, A. (2019). EFFECTS OF TEACHING MATHEMATICS THROUGH MUSIC ACTIVITIES REVISTA LATINOAMERICANA DE INVESTIGACION EN MATEMATICA EDUCATIVA-RELIME, 22(2), 163-184

Miranda da Silva, Evanir, dos Santos da Silva, Tassiane Araújo, de Souza Balk, Rodrigo, Ricardo Lopes, Robson, Caldeira Santos, Christian, Lara, Simone, Graup, Susane (2017). Avaliação do alinhamento postural e extensibilidade muscular pela escala SAROMM em crianças com paralisia cerebral após fisioterapia aquática Fisioterapia Brasil, 18(6), 719-726

Muzaffar, H., Nickols-Richardson, S. M. (2016). PAWS (Peer-education About Weight Steadiness) club: rationale and design for a randomized controlled study FASEB journal, 30(#issue#), #Pages#

Ouellet, Sylvie, Poliquin, Nicole (2012). La musicothérapie au service du développement de l'enfant ayant un trouble déficitaire de l'attention avec hyperactivité (TDAH)/ Music Therapy as Support in the Development of a Child with Attention Deficit Hyperactivity Disorder (ADHD) Canadian Journal of Music Therapy, 18(1), 79-90

Pizarro-Pino, D., Vilugron, G. A. F., Lagos-Hernandez, R. (2019). Cognitive and Motor Development Program for Selective and Sustained Attention in Children with ADHD REVISTA EDUCACION, 43(2), 411-425

Puyjarinet, F., Jeannin-Fuzier, A., Blain, C., Fournier, C., Metivier, M. (2020). Psychomotor therapy and attention deficit/hyperactivity disorder: Evaluation of a rhythm-based therapeutic program Neuropsychiatrie de l'Enfance et de l'Adolescence, 68(1), 22-28

Rabeyron, T., Saumon, O., Dozsa, N., Carasco, E., Bonnot, O. (2019). USING MUSIC THERAPY TO TREAT AUTISM SPECTRUM DISORDER IN CHILDREN: EVALUATION, PROCESS, AND MODELING PSYCHIATRIE DE L ENFANT, 62(1), 147-171

Reloba-Martínez, Sergio, Reigal-Garrido, Rafael Enrique, Hernández-Mendo, Antonio, Martínez-López, Emilio José, Martín-Tamayo, Ignacio, Chirosa-Ríos, Luis Javier (2017). Efectos del ejercicio físico extracurricular vigoroso sobre la atención de escolares / Effects of after-school, high-intensity physical activity programme, on levels of attention of school children Revista de Psicología del Deporte, 26(2), 29-36

Rothlisberger, M., Michel, E. (2009). Development and Evaluation of a Motor Coordination Training for Children in Special-needs Classes PRAXIS DER KINDERPSYCHOLOGIE UND KINDERPSYCHIATRIE, 58(3), 215-230

Rothmann, K., Hillmer, J. M., Hosser, D. (2014). Evaluation of the Musical Concentration Training with Pepe (MusiKo mit Pepe) for children with attention deficits ZEITSCHRIFT FUR KINDER-UND JUGENDPSYCHIATRIE UND PSYCHOTHERAPIE, 42(5), 325-335

Scheffner, E., Vorwerk, W., Vorwerk, U. (2017). Musical abilities in children with an auditory processing disorder LARYNGO-RHINO-OTOLOGIE, 96(8), 528-535

Vieira de Sá, Mariana, Lara, Simone, Graup, Susane, De Souza Balk, Rodrigo, Sasso, Renata Rosa (2018). Análise do desenvolvimento motor e da atenção de crianças submetidas a um programa de intervenção psicomotora ConScientiae Saude, 17(2), 187-195

Volzhentseva, I. (2017). DEVELOPMENT OF EXPRESSIVE-EMOTIONAL SIDES OF THE PRESCHOOL CHILD'S SPEECH BY MEANS OF ONTOPSYCHOLOGICAL MUSIC THERAPY PSYCHOLINGUISTICS, 22(1), 26-42

Zumaquero, Victoria Gallego, Mendo, Antonio HernÁNdez, Garrido, Rafael Enrique Reigal, De Mier, RocÍO JuÁRez Ruiz (2015). Efectos de la actividad física sobre el funcionamiento cognitivo en preadolescentes. / Effects of Physical Activity on Cognitive Functioning in a Sample of Preadolescent Children Apunts: Educació Física i Esports, #volume#(121), 20-27

Вовченко, Ольга (2016). ОСОБЛИВОСТІ ВИКОРИСТАННЯ МУЗИЧНО-РИТМІЧНИХ ЗАНЯТЬ ЯК ЗАСОБУ КОРЕКЦІЇ ПСИХОФІЗИЧНОГО РОЗВИТКУ ДІТЕЙ ІЗ ПОРУШЕННЯМИ СЛУХУ (Ukrainian) Features of use the musical-rhythmic lesson how method for correcting psychophysical development of children with hearing impairments. (English), 78(2), 26-33

Иванова, Нина (2016). УЧЕБНО-ПОЗНАВАТЕЛНАТА ЕВРИСТИЧНА ДЕЙНОСТ КАТО ФУНДАМЕНТ ЗА РАЗВИВАНЕ НА РЕФЛЕКСИВНИТЕ СПОСОБНОСТИ НА УЧЕНИЦИТЕ (Bulgarian) EDUCATIONAL-COGNITIVE HEURISTIC ACTIVITY AS A FOUNDATION FOR DEVELOPMENT OF STUDENT REFLEXIVE ABILITIES. (English), 24(5), 539-551

כרמל, לוי, מיכל, אלבוים-גביזון (2019). האם ההשפעה החיובית של הקראטה על מדדים מוטוריים, קוגניטיביים וחברתיים אצל ילדים טיפוסיים יכולה להוות רציונל לשימוש בקראטה ככלי טיפולי בילדים עם הפרעה התפתחותית בקואורדינציה? Journal of the Israeli Physical Therapy Society (JIPTS), 21(2), 40-53

امیر, دانا, روناک, رضائی (2022). اثربخشی آموزش تمرینات تعادلی بر تعادل ایستا و توجه انتخابی کودکان مبتلا به اختلال نقص توجه/ بیشفعال Shenakht Journal of Psychology & Psychiatry, 9(1), 151-163

امیر, دانا, روناک, رضائی, امیر, شمس (2021). تأثیر مداخله بازیهای فعال و تمرینات اگزرگیم بر کارکردهای اجرایی کودکان اوتیسم با عملکرد باا Shenakht Journal of Psychology & Psychiatry, 8(5), 113-125

درضيه, مرتسينى, علدونا صايرى, كاخكى, مندزه, ءربى, ذركس, جلالى (2021). ١ ساله با اختلال نارسايى 'نوجه ابيش ♦ تأثير تمرينات 'نعادلى بر 'نوجه با بدار .بسران ٧ ن*ا فعالى شهر كرمان The Effect of balance exercises on the sustained attention in boys between 7-10 years with Attention Deficit / Hyperactivity Disorder (ADHD) in Kerman., 1400(2), 100-112

سيد حسن حسيني, كل, مهدي, شهبازي, شهزاد, طهماسبي, فضل اله, باقرزاده (2022). تأثیر بازی های حرکتی و رایانه ای بر توجه و روابط اجتماعی دانش آموزان پسر 21 سال - مبتلا به اختلال نقص توجه/ بیشفعالی Shenakht Journal of Psychology & Psychiatry, 9(1), 1-15

فايزه كمالى, نؤاد, هحمدرضا صادقيان, شاهى, رزا رهاوى, عزآبادى (2023). نائير 'نمربنات حركتى وشناختى بر عملكرد حؤكتى و نوجه كودكان داراى اختلال هماهنكى وشدى The Effect of Motor and Cognitive Exercises on Motor Function and Attention among Children with Developmental Coordination Disorder., 8(2), 21-36

مهسا, استیلایی, ژاله, باقرلی, صالح, رفیعی, بهنام, ملکی (2024). تأثیر مداخله بازیهای حرکتی واقعیت مجازی بر کاهش علائم نقص توجه و تکانشگری در کودکان مبتلا به اختلال نقص توجه بیش فعالی Shenakht Journal of Psychology & Psychiatry, 11(1), 1-11

هانیه قاسمیان, مق, حسن, محمدزاده (2024). تمرینات بدنی هدف مدار بر کارکردهای شناختی کودکان با اختلال یادگیری The Effect of Goal-Oriented Physical Exercises on Cognitive Functions of Children with Learning Disorder., 16(3), 5-21

刘智妹, 蔡可龙, 朱丽娜, 熊轩, 董晓晓, 庞丽婷, 陈爱国 (2023). 运动干预对孤独症伴有智力低下儿童执行功能及默认网络功能连接的影响 Effects of Exercise Intervention on Executive Function and Default Network Functional Connectivity in Children with Autism Associated with Mental Retardation., 35(5), 493-502

簡馨瑩 (2020). 「記憶－抑制控制」活動融入語文教學對幼兒 在執行功能與口語理解表現的效果研究. (Chinese) Effects of Memory-Inhibitory Control Activity with Embedded Repeated Read-aloud Programs on Executive Function and Oral Comprehension Ability of Preschoolers. (English), 65(4), 275-304

赵梅玲 (2020). 两种训练干预方案对学龄前儿童体质与不同认知任务的影响. (Chinese) The Effects of Two Kinds of Training Intervention Programs on Physical Fitness and Different Cognitive Tasks of Preschool Children. (English), 43(5), 89-97

龐佳 (2015). 自閉症聽障兒童共享式注意力的手語舞蹈治療個案研究. (Chinese) The Single Subject Research on an Hearing-impaired Autistic Children's Joint Attention through Sign Dance Therapy. (English), #volume#(257), 88-104
